# Synthesis and Biological Evaluation of Novel *Dispiro*-Indolinones with Anticancer Activity

**DOI:** 10.3390/molecules28031325

**Published:** 2023-01-30

**Authors:** Yan A. Ivanenkov, Maxim E. Kukushkin, Anastasia A. Beloglazkina, Radik R. Shafikov, Alexander A. Barashkin, Andrey A. Ayginin, Marina S. Serebryakova, Alexander G. Majouga, Dmitry A. Skvortsov, Viktor A. Tafeenko, Elena K. Beloglazkina

**Affiliations:** 1 Chemistry Department, Moscow State University, Leninskie Gory 1/3, 119991 Moscow, Russia; 2 The Federal State Unitary Enterprise Dukhov Automatics Research Institute (VNIIA), 22. ul. Sushchevskaya, 127055 Moscow, Russia; 3 Shemyakin-Ovchinnikov Institute of Bioorganic Chemistry RAS, GSP-7, Ulitsa Mklukho-Maklaya 16/10, 17997 Moscow, Russia; 4 A. N. Belozersky Research Institute of Physico-Chemical Biology MSU, Leninskye Gory, House 1, Building 40, 119992 Moscow, Russia; 5 College of New Materials and Nanotechnologies, National University of Science and Technology MISiS, 119049 Moscow, Russia

**Keywords:** *dispiro*-indolinones, MDM2, p53, PPI, molecular docking, cytotoxicity, in vivo trials, anticancer activity

## Abstract

Novel variously substituted thiohydantoin-based *dispiro*-indolinones were prepared using a regio- and diastereoselective synthetic route from 5-arylidene-2-thiohydantoins, isatines, and sarcosine. The obtained molecules were subsequently evaluated in vitro against the cancer cell lines LNCaP, PC3, HCT^wt^, and HCT^(−/−)^. Several compounds demonstrated a relatively high cytotoxic activity vs. LNCaP cells (IC_50_ = 1.2–3.5 µM) and a reasonable selectivity index (SI = 3–10). Confocal microscopy revealed that the conjugate of propargyl-substituted *dispiro*-indolinone with the fluorescent dye Sulfo-Cy5-azide was mainly localized in the cytoplasm of HEK293 cells. P388-inoculated mice and HCT116-xenograft BALB/c nude mice were used to evaluate the anticancer activity of compound **29** in vivo. Particularly, the TGRI value for the P388 model was 93% at the final control timepoint. No mortality was registered among the population up to day 31 of the study. In the HCT116 xenograft model, the compound (170 mg/kg, i.p., o.d., 10 days) provided a T/C ratio close to 60% on day 8 after the treatment was completed. The therapeutic index—estimated as LD_50_/ED_50_—for compound **29** in mice was ≥2.5. Molecular docking studies were carried out to predict the possible binding modes of the examined molecules towards MDM2 as the feasible biological target. However, such a mechanism was not confirmed by Western blot data and, apparently, the synthesized compounds have a different mechanism of cytotoxic action.

## 1. Introduction

The transcription factor p53—an important tumor suppressor protein—is deeply implicated in a variety of vital intracellular events, especially in apoptosis, DNA repair, and cell-cycle regulation [1,2]. It plays a crucial role in the suppression of tumor growth and progression [1,2,3]. Although rodents with knockout of the *TP53* gene live normally, they are prone to the development of various types of cancer [4]. It has been established that, in more than half of the available model tumor cells, this protein is frequently mutated or silenced/deleted. This results in diminished apoptotic capacity and, as a consequence, provokes rapid tumor evolution [5]. This protein has been considered as an attractive biological target due to its unique ability to effectively eradicate many tumors. The functions of p53^wt^ in p53-“insensitive” cancer cells are also thought to be inadequate because of abnormalities in its regulation or perturbed signaling.

The activity of p53 can be blocked by the human murine double minute 2 (MDM2, also known as HDM2) oncoprotein [6]. In contrast to p53, the depletion of MDM2 in mice ultimately leads to lethal outcomes [7]. MDM2 binds the p53 transactivation domain with high specificity, thereby inhibiting its transcriptional activity and DNA binding, promoting the transport of p53 out of the nucleus, along with subsequent proteasome-mediated degradation [8]. However, p53 enhances the expression of the MDM2 protein via an autoregulatory negative feedback loop [9]. 

Momand and colleagues demonstrated that, among 4000 tumor samples of 28 different cancer types, an enhanced amplification of the MDM2 gene occurred in 7% of human cancers [10]. In addition to the abnormal level of MDM2 gene amplification, its overexpression was found to be caused by different mechanisms, including polymorphism (SNP309) in the TP53 gene promoter sequence, as well as enhanced transcription or translation [11,12,13,14]. In any case, an abundant level of MDM2 in cancer cells is directly associated with poor clinical prognosis, as well as with weaker responses to routine anticancer treatment. The blockade of MDM2/p53 protein–protein interaction (PPI) restores the normal tumor-suppressor function of p53. 

Considering the above points, MDM2 gene overexpression has been considered as an oncogenic driver in a variety of cancers [15]; therefore, this protein has been described as a promising biological target. The development of novel non-peptide small-molecule therapeutics that are able to block the hydrophobically driven MDM2–p53 PPI is now recognized as an attractive therapeutic option [16,17,18]. Several review articles have comprehensively summarized the recent progress achieved in the drug design and development of novel MDM2 inhibitors [1,17,19,20,21,22,23]. Almost all of the currently reported MDM2–p53 blockers fit the common topological pharmacophore that reflects the interaction of three key p53 residues with the conservative MDM2 PPI pocket [24]. These hotspots are assembled by Phe19, Trp23, and Leu26 and—in spite of other large, shallow, and often nondescript PPIs—they form a relatively deep cleft that is quite appropriate for targeting by small-molecule compounds. Hence, these residues can be regarded as valuable 3D probes for the design of novel MDM2–p53 PPI inhibitors. They share a “trident-like” topology that corresponds to *i*, *i + 4,* and *i + 7α*-helix PPI types. Excellent examples of *α*-helix mimetics can be found among these compounds.

**Figure 1:**
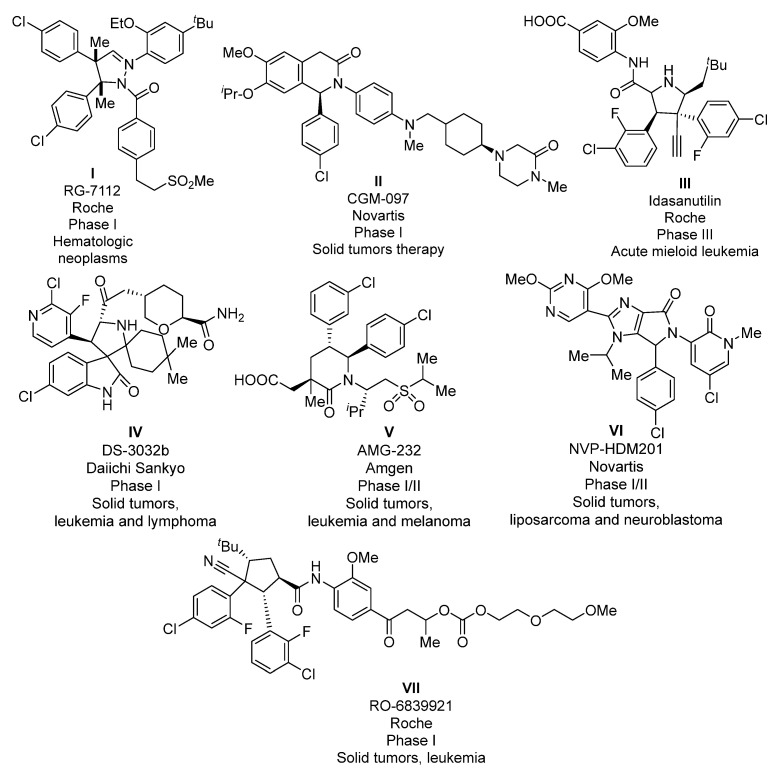
Small-molecule MDM2 inhibitors investigated in different clinical trials.

So far, more than 20 different chemotypes have been demonstrated to block this interaction [25,26,27,28]. These include nutlins [29], *spiro*- [30] and *dispiro*-oxindoles [31], benzodiazepinediones [32], *iso*-indolinones [33], chalcones [34], piperidinones [35], pyrrolidones [36], pyrrolopyrimidines [37], chromenotriazolopyrimidines [38], and xanthones [39], as well as quinolinols, chlorofusins, norbornanes, sulfonamides, terphenyls, and piperazine-4-phenyl derivatives (Figure 1). Among them, nutlins, *spiro*-oxindoles (e.g., MI series), and benzodiazepinediones (e.g., AM-8553) have demonstrated superior activity and are of clinical significance. 

Nutlins—*cis*-diphenyl-substituted imidazoline-containing compounds—were identified as the first class of highly potent, selective, non-peptide, orally active inhibitors of the MDM2–p53 PPI [30]. Five of the most advanced molecules from this series were developed by Roche, including nutlin-3a and RG-7112 (Figure 1, compound **I**). For instance, nutlin-3a strongly binds to MDM2 (*K*_i_ = 0.2 nM, FRET assay [40], IC_50_ value below 100 nM), and it has been extensively evaluated in different preclinical studies as an orally active anticancer therapeutic with minor side effects (e.g., weight loss) and no visible signs of serious toxicity [30,41]. The compound effectively inhibited the growth of many tumor cells—particularly LNCaP and HCT116, showing CC_50_ values of 1.50 µM (MTT assay) [42] and 4.63 ± 0.780 µM (dye assay, sulforhodamine B) [43,44], respectively. Nutlin-3a also blocked the growth and progression of PC3 cells, with a CC_50_ value of 6.37 ± 1.00 µM (dye assay with sulforhodamine B) [45]. However, no clinical trials have been reported for the compound, because of several reasons. It has been replaced by more active analogues with an optimized ADME profile. 

The “nutlin story” has led to the development of RG-7112 [21] and RO-5503781 [45]—two compounds produced by Roche, containing this scaffold. RG-7112 was undergoing phase I clinical trials against advanced solid tumors [46] and leukemia [47]. Under MTT tests, this compound demonstrated a relatively high cytotoxicity towards different cancer cell lines, including LNCaP and HCT116, with CC_50_ values of 0.18 µM [48] and 0.54 ± 0.12µM [49,50], respectively. However, no recent developments have been reported on this research. 

Idasanutlin (RO-5503781) was evaluated in a phase III clinical trial for the treatment of patients diagnosed with relapsed or refractory acute myeloid leukemia in combination with cytarabine (a DNA polymerase inhibitor) [51]. The drug was also investigated in early clinical trials (phase I/II studies) for the treatment of relapsed multiple myeloma in combination with ixazomib citrate (a proteasome inhibitor) and dexamethasone (a synthetic adrenocortical steroid), as well as against solid tumors, non-Hodgkin lymphoma (phase I studies), essential thrombocythemia, and polycythemia vera. Among MDM2 inhibitors in clinics, the chemical structures of compounds **I**–**VII** (Figure 1) have been disclosed; these examples clearly demonstrate the paramount clinical potential of MDM2 inhibitors as promising anticancer therapeutics.

**Figure 2:**
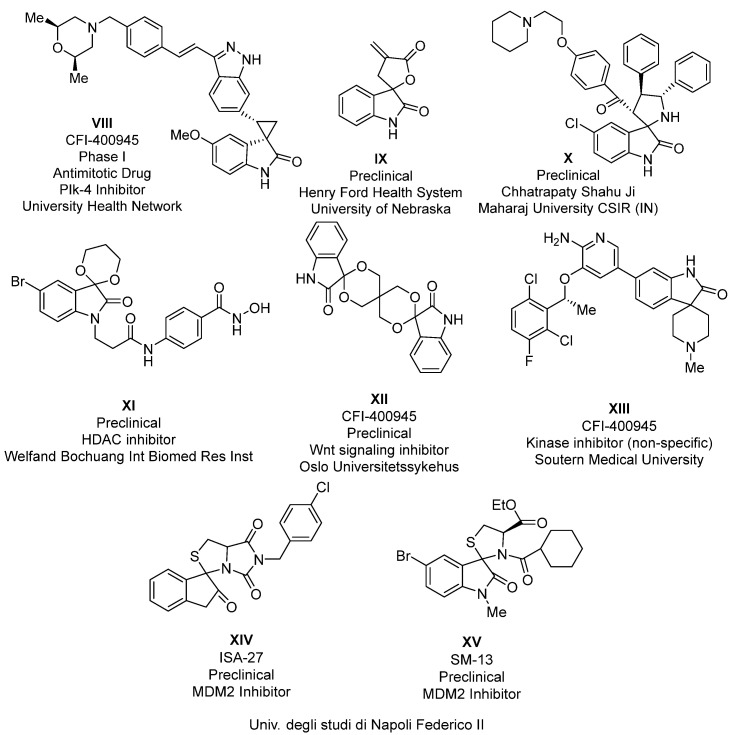
Representative examples of *spiro*-indolinones with anticancer activity.

*Spiro*-compounds have always been among the most attractive and privileged organic scaffolds in modern medicinal chemistry, due to their wide range of prominent physiological activities [52]. In particular, *spiro*-oxindoles and *spiro*-hydantoins or isosteric *spiro*-thiohydantoins have emerged as attractive synthetic templates because of their prevalence in a considerable number of natural-like products [53]. Alkaloids containing *spiro-*oxindole motifs were first isolated from plants of the *Apocynaceae* and *Rubiacae* families [54]. The basic structural feature of this type of compound is the *spiro* point at position 3 of the oxindole fragment. This joint can be formed by the attachment of heterocyclic motifs, thereby providing a considerable degree of diversity. As a result, *spiro*-oxindoles are reasonably regarded as appropriate templates for drug design and development. They can also be readily used as convenient starting points or intermediates in the synthesis of a wide range of structurally diverse natural-like products. A thorough search of the close structural analogues of the synthesized compounds (vide infra) revealed many *spiro*-indolinones with anticancer activity (compounds **VIII**–**XV**, Figure 2). Some compounds were reported as highly active MDM2–p53 PPI inhibitors.

Using an in silico structure-based de novo design approach, Wang and colleagues at the University of Michigan discovered the novel class of MDM2–p53 PPI inhibitors based on a *spiro*-oxindole scaffold (MI series) [55]. The compounds developed by Wang’s group possessed good PK properties as well as high binding affinity towards MDM2. Several of them, e.g., MI-43 and ISA-27, were evaluated in preclinical trials [56]. However, MI-43 was found to lack the desirable PK profile and was unsuitable for in vivo evaluation against models of human cancer. 

Subsequent chemical optimization of MI-43 led to MI-219 (*K*_i_ = 5 nM) as a novel analogue with improved in vivo bioavailability (at 50 mg/kg, oral bioavailability was 55–65% in mice), stability, and key PK features. MI-219 was also more effective than irinotecan at its maximum tolerated dose (MTD). Ascenta Therapeutics has purchased the license on MDM2 inhibitors of the MI series proposed by the groups from University of Michigan, and Sanofi-Aventis and Ascenta Therapeutics have signed an exclusive global collaboration and licensing agreement on a number of molecules that inhibit this PPI. 

Sanofi suspended the clinical development program for MI-219 and focused on the more potent analogue MI-773. The compound was evaluated in a phase I clinical trial against cancer. MI-888 is another potent inhibitor of p53–MDM2 PPI (*K*_i_ = 0.44 nM), which was also investigated in preclinical studies in Sanofi. The compound demonstrated a relatively high anticancer activity (IC_50_ ~90 nM against SJSA1 human osteosarcoma cells), achieving rapid, complete, and durable tumor regression in two types of xenograft models of human cancer upon oral administration [57]. However, in 2015, Sanofi discontinued the development of MI-773 and MI-888 due to financial reasons. 

Compound DS-3032b (IC_50_ = 0.183 µM against SJSA1 cells), by Daiichi Sankyo, is under active development in phase I clinical trials for the treatment of advanced solid tumors, lymphoma, leukemia, and multiple myeloma. The number of scientific publications and patents describing this scaffold has continued to increase over the past decade, especially in the field of anticancer drug therapy.

With regard to other chemotypes, many compounds have been disclosed with a high degree of diversity in their structure. For instance, MDM2–p53 inhibitors with IC_50_ values in the range of 24.1–181 μM we5860], morpholinone, and piperidinone derivatives [35,38,58,59,60,61,62], e.g., AM-6761 [63]. AM-7209 (IC_50_ = 1.6 nM against SJSA1 cells)—a potent and selective 4-amidobenzoic acid inhibitor of the MDM2–p53 PPI—is being evaluated in different preclinical trials [64]. AM-8553, also containing a piperidinone scaffold, was designed using a structure-based de novo strategy. The compound was revealed to have high activity both in vitro and in vivo, and it demonstrated oral bioavailability of 100% in rats [65].

**Figure 3:**
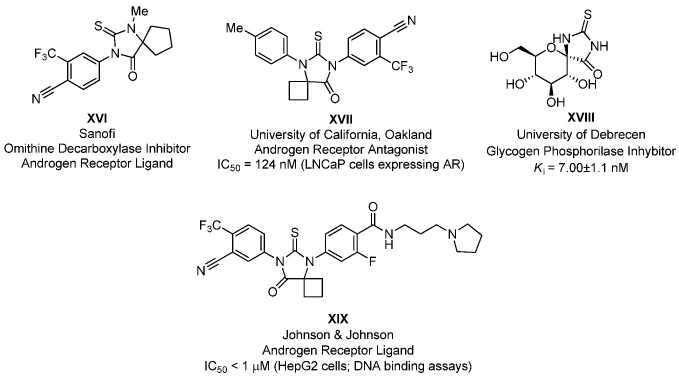
Examples of physiologically active *spiro*-thiohydantoins.

Unfortunately, *dispiro*-indolinones are described in the literature only as examples of aryl-substituted (3″R)-4,4-dimethyldispiro[cyclohexane-1,2′-pyrrolidine-3′,3″-indol]-2″(1″*H*)-ones by Daiichi Sankyo. These agents inhibited MDM2–p53 PPI with IC_50_ values in the range of 0.001–0.05 µM, resulting in effective blockage of human lung cancer NCI-H460 cell proliferation with wild-type p53 (GI_50_ < 0.1 μM, MTT assay). Several *spiro*-thiohydantoins also demonstrated high antitumor activity (compounds **VXII**–**XXI**, Figure 3) [35,66,67,68,69].

Hence, the coupling of two privileged scaffolds (i.e., *spiro*-oxindole and 2-thiohydantoin moieties) in the same molecule can provide compounds with a wide spectrum of physiological activity, including those with anticancer activity.

Previously, we reported a small series of *dispiro*-indolinone derivatives as possible inhibitors of p53–MDM2 PPI with anticancer activity in vitro [31]. Here, we describe a follow-up study on the synthesis and biological evaluation of a broader series of molecules containing the title scaffold, including in vivo trials.

## 2. Results and Discussion

### 2.1. Synthesis

There are a relatively small number of synthetic routes to *dispiro*-indolinones that have been reported to date. Ouyang and co-workers [70] reported a convenient synthetic pathway to substituted *dispiro*-oxindole derivatives (yields: 82–90%) via the three-component 1,3-dipolar cycloaddition of azomethine ylides with the dipolarophile 5-benzylideneimidazolidine-2,4-dione. An intermediate was generated in the reaction mixture by the decarboxylative condensation of isatin and an α-amino acid. A versatile synthesis of *dispiro*-oxindole-fused heterocycles (yields 78–89%) using a three-component reaction on the basis of domino 1,4-dipolar addition and the Diels–Alder reaction was also reported [71].

**Scheme 1:**
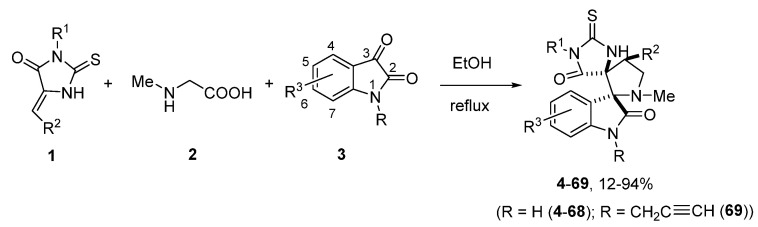
Synthesis of *dispiro*-indolinones **4**–**69**.

The compounds synthesized in our study represent substituted 2-thioxo-5*H*-dispiro[imidazolidine-4,3-pyrrolidine-2,3-indole]-2,5(1*H*)-diones, which were obtained based on the dipolar cycloaddition of azomethine ylides generated in situ by the decarboxylative condensation of isatin and sarcosine (*N*-methyl substituted glycine) through lactone decomposition with diaryl-substituted 2-thioxoimidazol-4-ones (Scheme 1 and Table 1) [31,72].

In accordance with the presented synthetic pathway, a series of compounds **4**–**74** was readily obtained using a two-step one-pot format. The following synthetic procedure turned out to be optimal (Scheme 1): the starting 5-arylmethylene-2-thiohydantoin **1** (1 equiv.) was synthesized as described in [31], sarcosine **2** (2 equiv.) and ethanol were loaded into the reaction flask, the mixture was brought with stirring to a boil, and then isatin **3** (2 equiv.) was added. The resulting mixture was refluxed for 5–8 h until a homogeneous solution was obtained and the initial 2-thiohydantoin was undetectable (according to TLC data).

**Figure 4:**
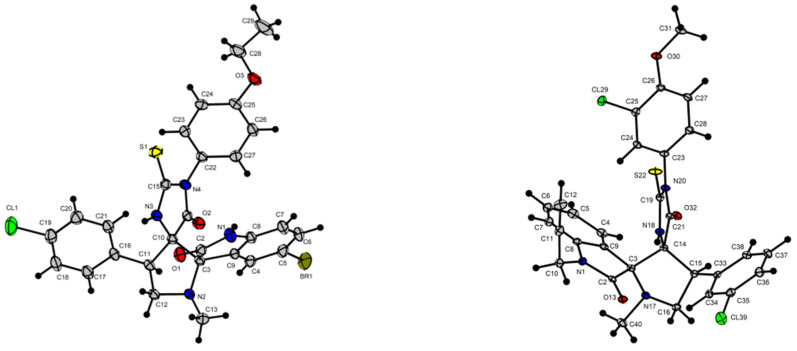
Molecular structures of *dipiro*-indolinones **29** (**left**) and **69** (**right**); atoms are displayed as thermal ellipsoids at a 30% probability level.

The desired diastereomerically pure *dispiro*-indolinones **4**–**69** (Scheme 1) were isolated with good yields (up to 89%, Table 1). The obtained compounds were characterized by NMR and HRMS spectra (see Supplementary Materials). The supposed mechanism of the performed reaction has been reported previously [73]. It includes the successive reactions of isatin with sarcosine that lead to iminium intermediates through the formation of lactone, and subsequent CO_2_ elimination resulted in azomethine ylide. This 1,3-dipole then regio- and diastereoselectively attacks the C=C double bond of 5-arylidenehydantoin. The formation of diastereomerically pure products with (S*,S*,R*)-configuration was proven on the basis of X-ray crystallographic analysis performed for the *dispiro*-indolinones **29** and **69** (Figure 4), as well as NMR spectral data for the reaction mixtures. According to the NMR spectra of the reaction mixtures, other diastereomers of compounds **4**–**69** were not formed under the reaction conditions, except for those shown in Scheme 1.

A typical ^1^H NMR spectrum of the resulting *dispiro*-indolinone is shown in Figure S1 in the Supplementary Materials, using the example of compound **27**. The characteristic signals are the singlets of the amide protons of the indolinone and imidazolone cycles in the region of ~10–11 ppm, the singlet of N-CH_3_ group protons at 2.1–2.2 ppm, and the signals of three protons at *sp^3^*-hybrid atoms of the central pyrrolidine ring in the region of 3.3–4.4 ppm, which are pseudotriplets with J at about 9.0 Hz. In some cases, the structure and stereochemistry of the obtained *dispiro*-compounds was further confirmed using 2D NMR spectroscopy data (see Figures S2–S4).

### 2.2. Molecular Docking Study

**Figure 5:**
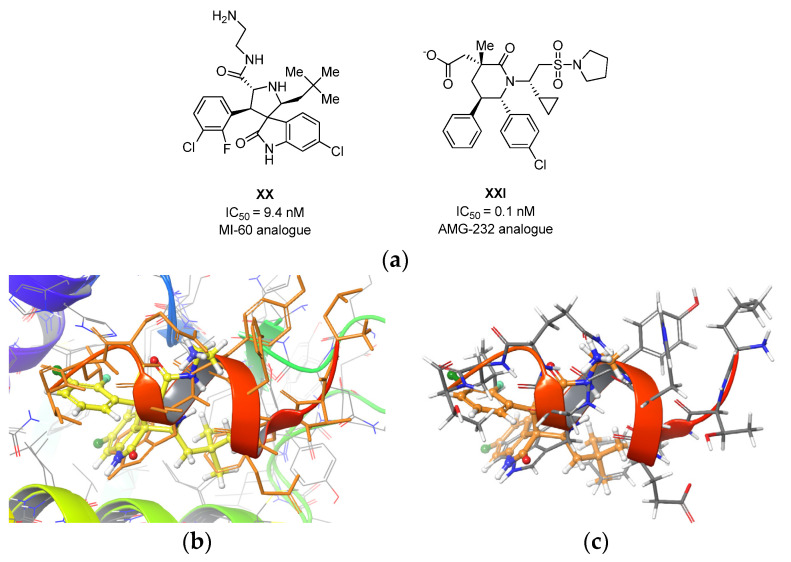
(**a**) Structures of the template molecules **XX** and **XXI** used for modeling; (**b**,**c**) the overlapping of compound **XX** (yellow) with the hotspots in the domain recognition element of the p53 peptide (orange); three key hydrophobic binding points and an α-helix mimetic core.

The first high-resolution *co-*crystal structure of MDM2 in complex with a fragment of p53 peptide responsible for binding (1YCR) was reported by Kussie and colleagues [74]. The PPI interface observed between these proteins is formed by the first ∼120 *N*-terminal amino acid (AA) residues of MDM2 and the first 30 *N*-terminal AAs of p53 (Figure 5) [75]. Since then, more than 50 crystallographic complexes have been published for small-molecule MDM2–p53 inhibitors with high diversity in structure, including *spiro*-indolinones. The analysis of the MDM2–p53 binding mode indicated that the p53 peptide adopts an α-helical conformation and interacts with MDM2 primarily via the hydrophobic triad of Phe19, Trp23, and Leu26. This anchoring “*trident*” of *i*, *i* + 4, and *i* + 7 PPI hotspots binds a medium-sized pocket in the structure of MDM2. This site has been used to design a number of different small-molecule MDM2 inhibitors that effectively prevent the MDM2–p53 interaction, thereby blocking tumor growth and progression. To elucidate a possible mechanism of action of the synthesized compounds against the MDM2 protein, we carried out molecular docking studies using MOE software [12]. The model was constructed based on three crystallographic complexes: a p53 peptide bound to MDM2 [76], as well as compounds **XX** and **XXI** [48,77,78]. Figure 5b depicts a 2.0 Å resolution structure of the stapled p53 peptide bound to the target protein. The *i*, *i* + 7 peptide is conformationally and proteolytically stabilized with all-hydrocarbon staples and remains quite capable of disrupting the described PPI via a tight binding with the conservative MDM2 pocket.

The binding site was reconstructed and compared with the binding mode disclosed for the recombinant p53 peptide [79]. Water molecules were excluded from the modeling procedure. A three-centered 3D pharmacophore model containing three key hydrophobic binding points (see above) was then constructed and used during the docking procedure as a rigid frame to identify and maintain the correct position of the compounds in accordance with the X-ray data. This hybrid model restricts the pool of hypothetical “active” conformations to the most appropriate and reliable ones. The reference compounds **XX** and **XXI** were then docked into the developed model, keeping the reported stereo configuration. The obtained results were well correlated with the X-ray structures published previously. For instance, the predicted conformation of compound **XX** was very similar to that revealed by crystallographic analysis, with an r.m.s.d. value of 1.05 Å. As shown in Figure 5b, *tert*-butyl and *o,m-*dihalophenyl moieties of compound **XX** fit into the Phe19 and Leu26 pockets, respectively. The latter interaction is maintained by a π−π stacking arrangement with His96; however, if this residue is protonated upon a pH value equal or below pKa, π–cation interaction is realized instead. It should be noted that several piperidinone-containing MDM2 inhibitors (for instance, compound **XXI**) interact with the Phe19 pocket through one chlorophenyl fragment, while the other phenyl moiety occupies the Leu26 sub-pocket, in contrast to compound **XX**. Thus, there are striking differences in binding mode observed for these two inhibitors [76]. On the other hand, compound **XXI,** despite bearing the same core, binds the MDM2 pocket in a similar manner to indolinone **XX** (Figure 5c).

**Figure 6:**
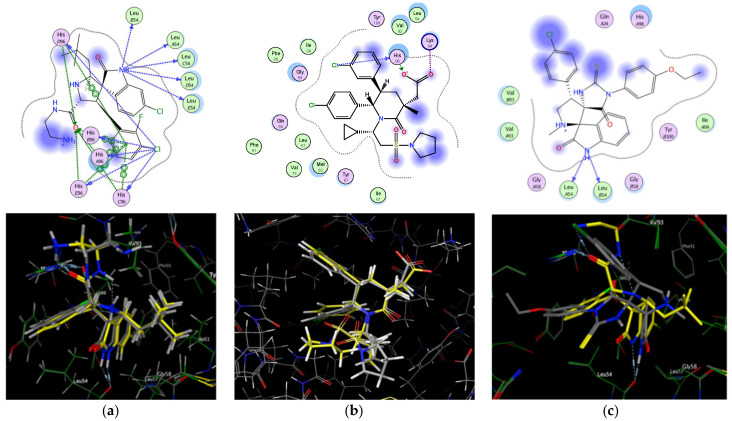
Compound **XX** (**a**), compound **XXI** (**b**), and compound **29** (**c**) docked in the MDM2 pocket. *Yellow*: X-ray data for compounds **XX** (**a**) (PDB code: 4JVR, resolution: 1.70 Å) and **XXI** (**b**) (PDB code: 4QOC, resolution: 1.70 Å); *grey**—***docking results. For (**a**): E^score^ = −8.49 kcal/mol; for (**b**): E^score^ = −7.62 kcal/mol; for (**c**): E^score^ = −5.31 (compound **26**); low E^score^ values correspond to more favorable binding.

We used the crystal structures of compound **29** and compound **74** solved from single-crystal X-ray diffraction (see Figure 4) with predefined geometry during the follow-up docking study. As shown in Figure 6c, compound **29** favorably fitted the pharmacophore hypothesis and was positioned within the binding site in a manner similar to that revealed for compound **XX**. The predicted binding conformation was similar to that published for the MDM2 inhibitors of the MI series. However, in contrast to the template molecule, compound **29** showed an almost twofold lower score value that, in turn, resulted in weaker p53 activation activity (see below). As in the case of morpholinone-based MDM2 inhibitors, we also obtained several alternative conformations with similar binding energy, where the methyl group of compound **29** is located in a deep cavity, similar to the reported 5-halogen-substituted indolinones. As expected, *N*-substituted indolinone **74** did not show anticancer activity and did not pass in silico evaluation, providing a relatively weak score value (E^score^ over −2 kcal/mol).

### 2.3. Biological Evaluation In Vitro

#### 2.3.1. Cytotoxicity

The synthesized compounds were tested for their ability to block the proliferation of the selected model cell lines: LNCaP (p53+), PC3 (p53-), HCT^wt^ (p53+), and HCT^−/−^ (p53-) cells. The assay was carried out using the standard MTT (3-(4,5-dimethylthiazol-2-yl)2,5-diphenyl tetrazolium bromide) assay based on the modified approach reported by Ferrari and colleagues [79]. LNCaP and HCT^wt^ cells expressed p53 (positive cells), while PC3 and HCT^−/−^ did not express p53 (negative). Differences in IC_50_ values against these cell lines were used to assess the selectivity of the compounds. The cytotoxic activity of the evaluated compounds is summarized in Table 2.

As shown in Table 2, the most active compounds from the series (compounds **13**, **18**, **29,** and **63**) exhibited IC_50_ values of ~1–3.5 µM against LNCaP cells. These molecules contain a halogen atom in position 5 of the isatin fragment (Br for compounds **13**, **29,** and **63,** or Cl for compound **18**). Some molecules demonstrated a good selectivity index for LNCaP–PC3 and/or HCT^wt^–HCT^−/−^ pairs (SI = 3 to 10). Compound **13** showed an SI value similar to that observed for nutlin-3a, with a proven mechanism of cytotoxic action as an inhibitor of the p53–MDM2 interaction.

#### 2.3.2. Intracellular Accumulation

**Scheme 2:**
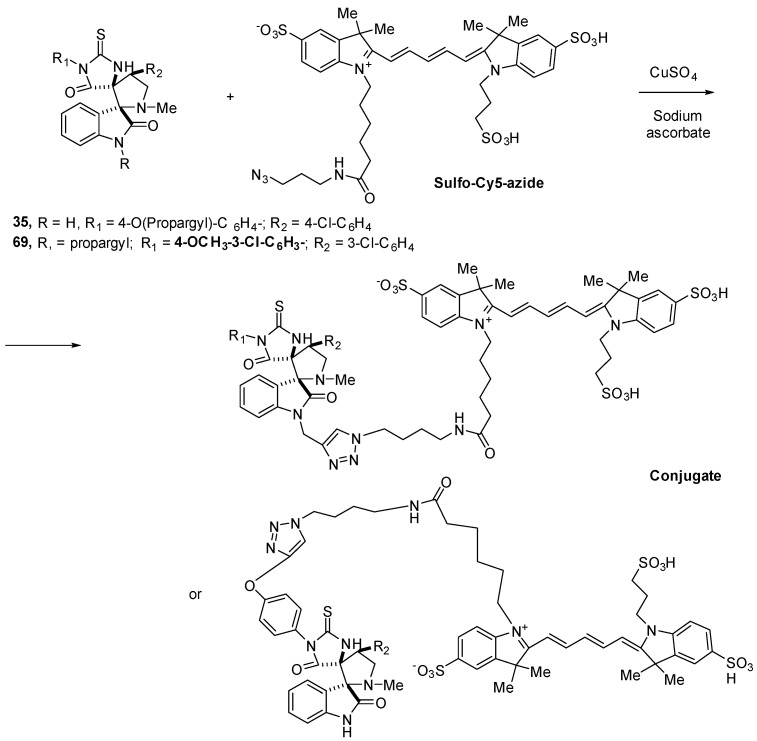
Click reaction between the fluorescent dye Sulfo-Cy5-azide and *dispiro*-indolinones **69** and **35**.

To investigate the intracellular localization, we synthesized propargyl-based compounds **69** and **35**, the latter of which is the propargylated analogue of one of the most cytotoxic *dispiro*-indolinone **29** (Scheme 2)**,** equipped with the fluorescent dye (Sulfo-Cy5-azide) via a click reaction inside the cell (no intrinsic fluorescence was observed for the obtained conjugate). Human embryonic kidney cells (HEK293) or prostate cancer cells (22RV1) were treated with *N*-propargyl derivatives **69** or **70** and then stained with Sulfo-Cy5-azide in the presence of CuSO_4_ and sodium ascorbate.

**Figure 7:**
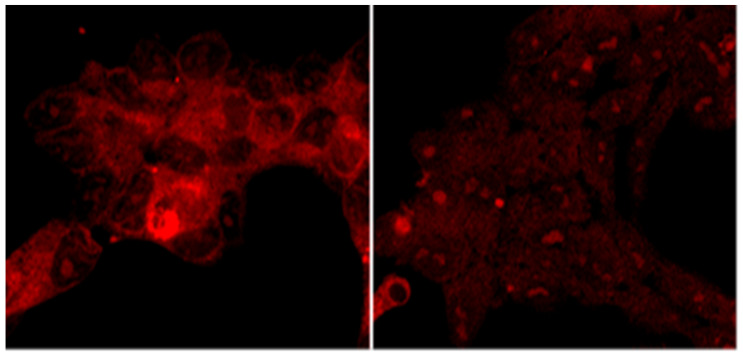
The localization of the compound **35**–Sulfo-Cy5-azide conjugate in 22RV1 cells ((**left**) stained by click reaction product; (**right**) Sulfo-Cy5-azide only).

Based on the obtained results, we can conclude that compound **69** mainly localizes in the cytoplasm. Compound **35** similarly does not have a significant distribution pattern in the cell, and it is spread over the cell, but it is somewhat more observed in the cytoplasm than in the nucleus and demonstrates increased cell membrane staining, which is consistent with high hydrophobicity. An example of the fluorescence of the labeled compound (conjugate) in microphotographs obtained by confocal microscopy is shown in Figure 7. 

#### 2.3.3. Western Blot, Mechanism of Cell Death, and 2D Electrophoresis

To verify the mechanism of cytotoxic action of the synthesized *dispiro*-indolinones, Western blot testing of *dispiro*-indolinone **29** was performed using the p53-expressing HCTwt and LNCaP cell lines. Both the p53 protein and the proteins involved in the p53-dependent process of apoptosis (i.e., BAX, NOXA, and PUMA) were determined. For comparison, similar testing was carried out for a cell line not expressing p53 (HCT^(−/−)^).

**Figure 8:**
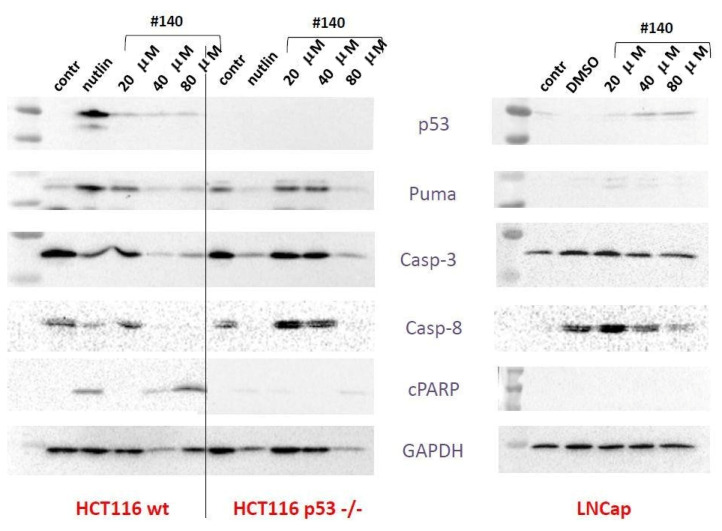
Western blot data showing the effects of compound **29** on the expression of the p53 and PUMA proteins, as well as caspase-3 and caspase-8—caspases involved in p53-dependent apoptosis in HCTwt, HCT^(−/−)^,and LNCaP cells. Nutlin-3a (20 µM) was used as a positive control.

According to the obtained data (Figure 8), with an increase in the concentration of compound **29**, a slight increase in the concentration of the p53 protein in the HCTwt cell culture was observed, which may include inhibition of the p53–MDM2 interaction by *dispiro*-indolinone. An increase in the expression of the pro-apoptotic proteins PUMA and CASPASE-3 was also detected. However, the treatment with nutlin-3a, for which the mechanism of action through inhibition of the p53–MDM2 interaction was confirmed, demonstrated a stronger increase in the expression of the p53 protein. Thus, p53 activation is not the main mechanism of **29’s** cytotoxic action.

The mechanism of cell death (i.e., necrosis/apoptosis) caused by one of the most cytotoxic compounds (**13**) was investigated by flow cytometry with staining by 7AAD/annexin V. HCT116p53−/− mainly showed necrosis/late apoptosis, HCT116wt predominantly showed early apoptosis, and PC3 also showed early apoptosis (Figure S5).

To further study the action of **29,** we investigated the changes in the protein landscape with 2D protein gel electrophoresis (Table 1 and Figure S6). As some proteins’ expressions patterns differ in HCT116wt and HCT116p53−/− cells after treatment, and 2D protein gel electrophoresis is semi-quantitative, we looked for associations of the pool of proteins whose expression was changed. There are no common edges in the known transcription regulatory networks in accordance with “http://www.licpathway.net/KnockTFv2 (accessed on 10 November 2022)”. The pool consisted of proteins, associated with (either) mitochondria and (or) protein folding (in accordance with Gene Ontology by string-db.org). Taken together, we may speculate that we saw the secondary effects as responses to stress and apoptosis, which masked the initial changes caused by **29**.

Taking into account its high cytotoxicity, sufficient selectivity, and ability to activate pro-apoptotic cascades according to the results of Western blotting, compound **29** was chosen for further biological studies in vivo.

### 2.4. Biological Evaluation In Vivo

All animal procedures were conducted in strict adherence to the European Convention for the Protection of Vertebrate Animals Used for Experimental and other Scientific Purposes (ETS 123), Strasbourg, 1986. To preliminary estimate the antitumor effect of the compound in vivo, transplant P388 and HCT116 xenograft mouse models were used. For intraperitoneal (i.p.) administration, compound **29** was dissolved with Pluronic^®^ F-127. The sample contained the active compound (17%) and the solubilizing agent (83%). The final 4.8% suspension was prepared at pH = 7.0 in sterile distilled water. For peroral (p.o.) administration, we used a 3.4% suspension of the tested compound in 1% starch-based thickener, which was prepared repeatedly ex tempore.

#### 2.4.1. P388 Model

Prior to the trials, the animals were split into different groups (five mice per group). One group was monitored as a control population, where the animals were kept untreated, while the other groups were subjected to the tested compound. The anticancer effect of the evaluated sample was assessed in model mice at the dose range of 295–1176 mg/kg. Four independent trials were carried out.

For the first evaluation, the sample was initially injected i.p. on day 2 of the study, and the treatment was subsequently continued during the following 9 days (total 10 d course). The typical doses were calculated considering the average body weight of the animals (20 g) and ranged between groups 1 and 3; group 4 was assigned as a control population. Thus, the first group (g1) was daily treated with the investigated sample at a dose of 295 mg/kg (cumulative dose was 2950 mg/kg), the second group was given 882 mg/kg (8820 mg/kg), and the third group was given 1176 mg/kg (11,760 mg/kg). To verify the obtained results, we performed the second evaluation using the following schedule: the first group was administered i.p. with 500 mg/kg (5000 mg/kg), the second group was given 750 mg/kg (7500 mg/kg), and the third group was given 1000 mg/kg (10,000 mg/kg). We also carried out the third trial with an appropriate dose of 1000 mg/kg. For p.o. administration (trial 4), we used a single dose (170 mg/kg) of the unmodified compound; the cumulative dose was 1700 mg/kg.

##### First Trial

**Figure 9:**
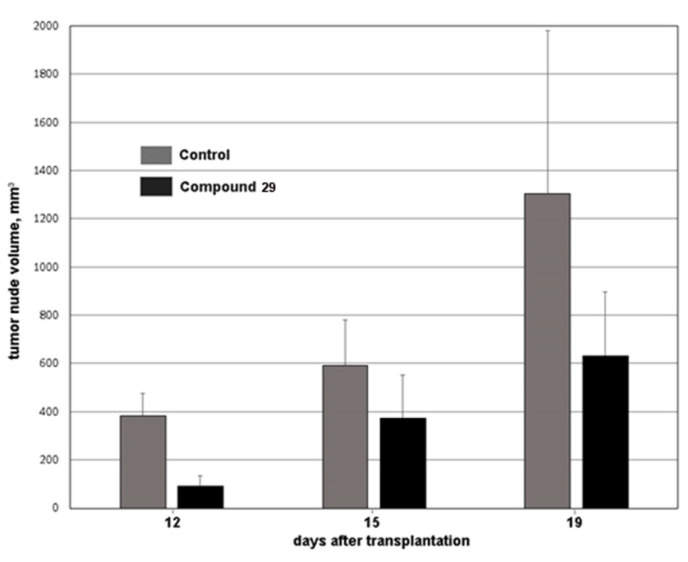
Changes in P388 volume upon the treatment with compound **29** (1176 mg/kg, o.d., 10 days, i.p.).

**Figure 10:**
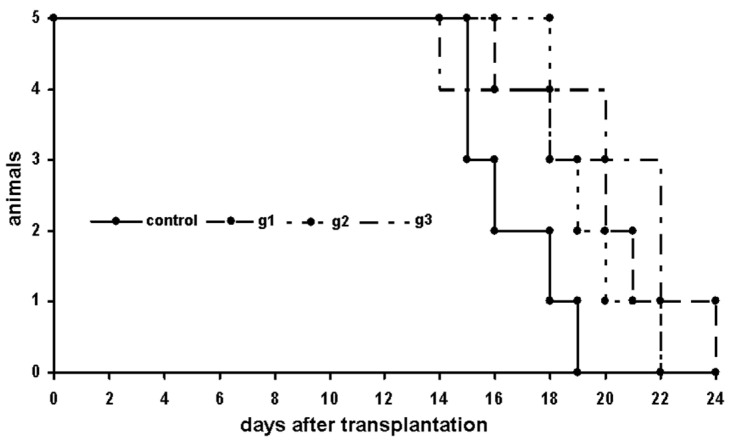
Lifespan observed among the P388-inoculated mice after the treatment (o.d., 10 days i.p.) with compound **29** at doses of 295 mg/kg (g1), 882 mg/kg (g2), and 1176 mg/kg (g3).

Within the control group (g4), it was shown that at three timepoints (days 12, 15, and 19 after inoculation) the cancer nodes of the P388 cells grew rapidly and normally, reaching 383 ± 93 mm^3^, 591 ± 189 mm^3^, and 1304 ± 676 mm^3^ in volume, respectively. The death outcome was registered on days 15–19 (Figure 9; average life span (ALS) = 16.6 ± 1.8 d). Two mice in the group were still alive on day 8 after the treatment period had ended. It was revealed that at doses of 295 mg/kg (g1) and 882 mg/kg (g2), the cancer growth rate was similar to that observed in the control group. The treatment resulted in a relatively good efficiency: TGRI (tumor growth rate inhibition) = 32–37% (*p* > 0.05) and T/C < 42%. The lethal outcomes were registered on days 16–24 and 18–22, respectively. Several mice stayed alive for more than 20 days; however, the gain in survival was statistically insignificant: ALS = 19.8 ± 3.0 d and 19.4 ± 1.7 d, respectively, and LSE (lifespan extension) = 17% and 19% (*p* > 0.05), respectively, vs. ALS = 16.6 ± 1.8 d within the control group. The maximum therapeutic effect was achieved with the daily dose of 1176 mg/kg (11,760 mg/kg, g3) 24 h after the treatment was discontinued (TGRI = 76%, T^test^ = 0.0002, *p* < 0.05). During the subsequent three days, this effect was reduced to TGRI = 40%, providing a relatively high dispersion value. Deaths were observed on days 14–22; on day 8 after the course was stopped, four mice were feeling well and stayed alive for more than 20 days. The administration of the tested sample at the highest dose of 1263 mg/kg (12,630 mg/kg), close to the MTD value, resulted in one mouse (bw = 19 g) dying on day 3 after the treatment was stopped. The obtained results are depicted in Figure 9 and Figure 10.

##### Second Trial

Since we did not obtain statistically significant results during the first trial due to an inappropriate dispersion at a relatively high dose (although the TGRI value reached 76% (*p* < 0.0002)), we used doses of 500 and 750 mg/kg o.d. for the second trial. At the higher dose, on day 2 after the treatment was completed, we obtained almost identical results, reaching TGRI = 68% (*p* = 0.002) vs. TGRI = 76% (*p* = 0.0002). Moreover, during the subsequent 3 days, the TGRI rate was kept at a statistically significant level of 62% (T^test^ = 0.02, *p* < 0.05). Lower doses did not provide any statistically significant results.

##### Third Trial

**Figure 11:**
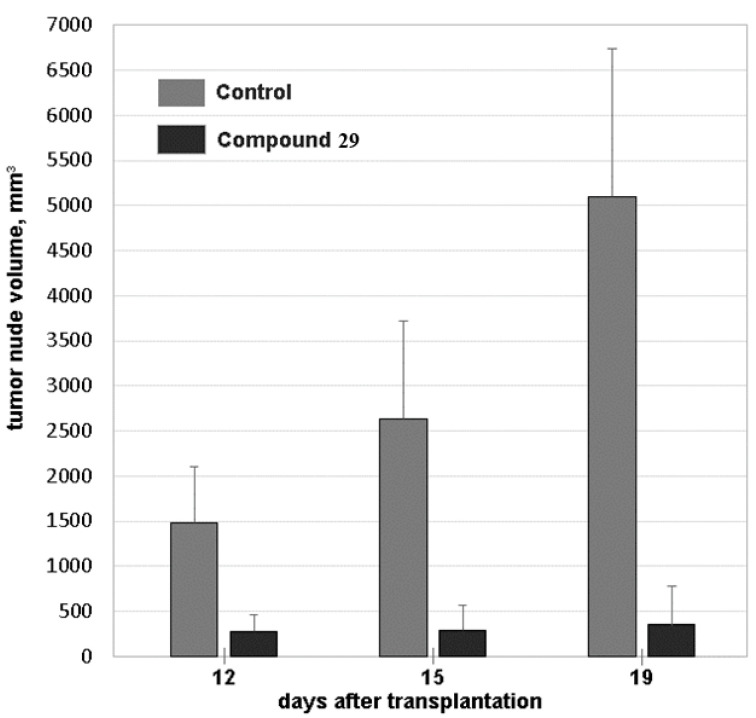
Changes in P388 nude volume upon the treatment with compound **29** (1000 mg/kg, o.d., 10 days, i.p.) as compared to the control group.

The trial was carried out with a dose of 1000 mg/kg o.d. (cumulative dose of 10,000 mg/kg) under the same conditions. On day 2 after the treatment was completed, we obtained superior results (Figure 11), reaching TGRI = 81.3% vs. TGRI = 76%. During the following 3 days, the TGRI value was increased up to 89% (T^test^ = 0.002), maintaining a statistically significant level. On day 8 after the treatment was discontinued, the TGRI value reached 93% (T^test^ = 0.001); the ALS index was also very high, at 27.6 ± 0.5 d (LSE = 33.33%). It should be noted that on day 31 of the study no mortality was observed among the treated population.

##### Fourth Trial

Unmodified compound **29** was also administered p.o. at a single dose of 170 mg/kg (o.d., 10 days). The obtained results are summarized in Table S2. No statistically significant reduction in tumor volume was registered, and no toxic effects were observed. Hence, we can conclude that at a dose of 170 mg/kg compound **29** is ineffective after p.o. administration.

As a result, the treatment of P388 mice with the tested substance at a dose of 1000 mg/kg (10,000 mg/kg, i.p., cumulative dose) led to a statistically relevant and reproducible outcome. The best anticancer effect considering the principal screening criteria (TGRI ≥ 50%) was achieved at the TGRI level of 93%; however, this dose was close to the MTD value. It should be noted that at a dose of 500 mg/kg the TGRI value was approx. 50%, whereas the administration of the sample at a dose of 1200 mg/kg resulted in lethal outcomes.

#### 2.4.2. HCT116 Model

**Figure 12:**
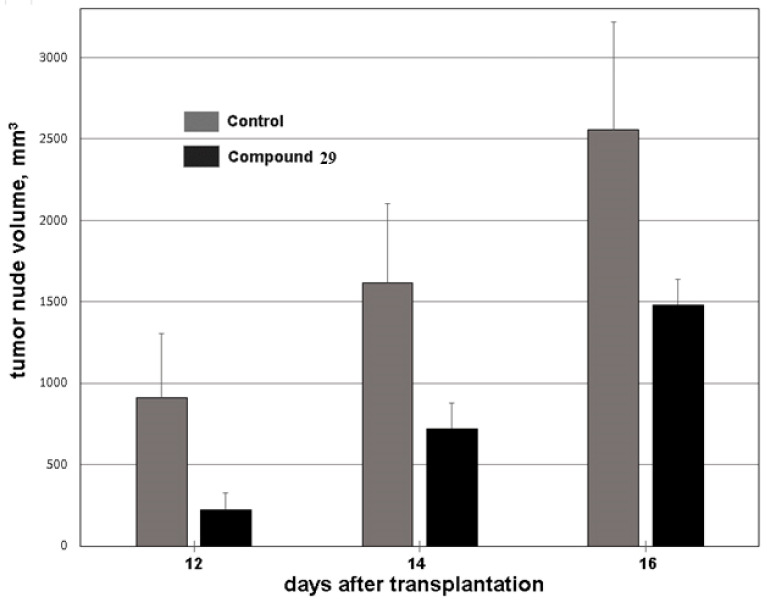
Changes in HCT116 tumor xenografts’ volume upon treatment with compound **29** (1000 mg/kg, o.d., 10 days, i.p.).

Considering the effective dose indicated previously (1000 mg/kg, o.d., 10 days), HCT116 xenograft BALB/c nude mice were treated i.p. with the tested sample using the same time and dose schedule. Tolerance and lifespan were not assessed because immunodeficient animals were used. Within the control group, HCT116 tumor xenografts grew normally, reaching 909 ± 396 mm^3^, 1616 ± 486 mm^3^, and 2554 ± 663 mm^3^ in average volume on days 12, 14, and 16, respectively. In the treated group, after the course was completed, statistically significant results were obtained at a T/C (treatment/control) level of 25% (*p* = 0.00005, after 24 h) vs. the P388 model (T/C = 133%, TGRI = 93%) at the final control timepoint (i.e., day 19 of the study). This effect was reduced to T/C = 44% (*p* = 0.0007, after 4 days) and T/C = 58% (*p* = 0.009, after day 8, Figure 12).

Based on the obtained results, it can be rationally concluded that compound **29** demonstrates significantly relevant anticancer activity at a daily dose of 1000 mg/kg (i.p., 10 days) in HCT116-bearing xenograft mice. 

#### 2.4.3. Acute Toxicity

This study aimed to estimate the toxicity of compound **29** administered intraperitoneally and by intravenous injection. The objectives of the study included the analysis of the clinical picture of intoxication, the analysis of the reversibility of the revealed toxic effects, and the identification of the most sensitive organs. Acute toxicity tests were performed on mature outbred mice (male and female). The design of the experiment is specified in Table S3 (Supplementary Materials).

To select the doses, the pilot experiments were carried out on a minimum number of animals. In a preliminary experiment, it was found that with intraperitoneal administration of compound **29** at a dose of 2500 mg/kg, there were no deaths among the animals. When administered in large doses during autopsy in the abdominal cavities of the mice, on the inner wall and membranes of the abdominal organs, remnants of the non-absorbed drug were found in the form of white layers, indicating incomplete absorption and insufficient solubility of the agent. Therefore, due to the low solubility of the test substance in aqueous media, it turned out to be impossible to obtain reliable experimental data with the introduction of compound **29** at doses > 2500 mg/kg. According to the data obtained from the trial experiment, in the main study—with intraperitoneal administration—a dose of 2500 mg/kg was used.

The observation period was 28 days. With intraperitoneal administration of compound **29** in mice, no deaths were registered. The surviving animals were subjected to euthanasia and necropsy on the 29th day of the experiment to study the effects of the test agent on their internal organs.

The clinical picture of intoxication with intraperitoneal administration of compound **29** was characterized by a depressed state, a decrease in motor activity, and the release of porphyrin. Symptoms of intoxication disappeared after 30–60 min. An assessment of the dynamics of body weight in animals that underwent intoxication did not show differences from the control mice. Autopsy of experimental animals on the 29th day after exposure to the drug revealed an increase in the liver, with signs of dystrophy (Table S4).

Thus, it can be concluded that intraperitoneal administration of *dispiro*-derivative **29** has a toxic effect on the bodies of mice. At the same time, deaths of the animals were not recorded when a dose of 2500 mg/kg was administered. Thus, the therapeutic index (estimated as LD_50_/ED_50_) for compound **29** in mice is at least 2.5. 

## 3. Materials and Methods

Commercially available chemicals were purchased from Sigma-Aldrich. The ^1^H NMR spectra of all synthesized compounds were recorded on Bruker Advance 400 spectrometer at 400 MHz in DMSO-*d*_6_. Chemical shifts (δ) are reported in parts per million (ppm) downfield from TMS and referenced from solvent references. Coupling constants are reported in *Hz*. The elemental compositions of the compounds determined by HRMS were matched within ± 0.4% of the calculated values. The reactions were followed by thin-layer chromatography (TLC) on Merck aluminum silica gel sheets that were visualized under a UV lamp. 

The data of compound **29** were collected by using a STOE diffractometer and a Pilatus100K detector, focusing mirror collimation Cu Kα (1.54086 Å) radiation, in rotation method mode. STOE X-AREA software was used for cell refinement and data reduction. Data collection and image processing were performed using X-Area 1.67 (STOE and Cie GmbH, Darmstadt, Germany, 2013). Intensity data were scaled with LANA (part of X-Area) in order to minimize differences in the intensities of symmetry-equivalent reflections (multi-scan method). 

X-ray investigations of compound **69** were performed using a SMART APEX DUO CCD diffractometer (Mo Kα radiation (0.71073 Å), graphite monochromator, φ- and ω-scan). Both structures were solved by direct methods and refined using full-matrix anisotropic approximation for F^2^_hkl_. The location of hydrogen atoms (CH) was predicted geometrically, and their positions were well adjusted using the “rider” model U_iso_(H) = 1.5 U_eq_(C) for methyl groups and 1.2 U_eq_(X) for the remaining H atoms. The location of hydrogen atoms (NH) was predicted using difference Fourier maps and was isotropically specified. All calculations were performed using SHELX software version 2009-9.13 [80]. CCDC-2217004 (compound **29**) and CCDC-2217008 (compound **69**) contain the supplementary crystallographic data for this paper. The data can be obtained free of charge from the Cambridge Crystallographic Data Centre via www.ccdc.cam.ac.uk/data_request/cif website (accessed on 10 January 2023).

### 3.1. General Procedure for the Synthesis of Compounds **4**–**69**

Isatin **3** (0.3 mmol) was added to a refluxing solution of 5-arylidene-2-thiohydantoin **1** (0.3 mmol) and sarcosine **2** (0.6 mmol) in ethanol (10–15 mL), and the resulting mixture was boiled for 6 h, monitoring the completion of the reaction by TLC. After cooling, the reaction mixture was poured into 100 mL of cold water, and the precipitate that formed was filtered off and recrystallized from ethanol. The synthesized compounds were characterized by NMR and HRMS spectra (see Supplementary Materials).

### 3.2. Molecular Docking

The crystal structures of small-molecule compounds in complex with MDM2 protein were obtained from the PDB collection [81]. The binding sites were reconstructed and prepared using MOE Software following the routine procedure. Several complexes were then superposed to analyze the conformational dynamics that occurred upon ligand binding. It was considered that water molecules did not significantly influence the docking results. The constructed model was then verified using some MDM2 inhibitors with different structures. Satisfactory results were obtained, providing predicted 3D conformations close to those disclosed by X-ray analysis. After the refinement procedure was completed, two compounds from our series characterized by RSA were docked into the developed model. To restrict the possible conformational space, we designed a 3-centered 3D pharmacophore model, which was integrated into the binding site. Docking was performed in a dynamic mode, where amino acids that organized the active binding site, as along with amino acids that were located in close proximity to the pocket, were also perturbed during the ligand positioning. Several possible active conformations with low energy scores were outputted from each docking simulation. An appropriate conformation was then assessed by a visual inspection and on the basis of energy score values and expert opinion.

### 3.3. Biological Evaluation

#### 3.3.1. In Vitro Assay

Model cell lines were purchased from the in stock collection of the N.N. Blokhin Russian Cancer Research Center (RAMS) [82]. Cells were accurately maintained in the recommended media, supplemented with 10% heat-inactivated FBS (Invitrogen) and 2 mmol/L L-glutamine (Invitrogen).

#### 3.3.2. MTT test

First, 4000 cells per well were plated out in 140 µL of DMEM (with 10% FBS) in 96-well plates and incubated in 5% CO_2_ for 24 h at 37 ˚C without treatment. Then, 15 µL of water–DMSO solutions (several final solutions were opalescent, one looked like a suspension, and the compounds were poorly soluble) of the tested substances were added to the cells (the DMSO concentrations in the media were 1% or less). The cells were treated for 48 h with 45–100 µM (eight dilutions) concentrations of the substances (each in triplicate) or doxorubicin (0.3 nM–600 nM, eight dilutions) as a control substance. After the incubation, 15 µL of MTT solution in PBS (5 mg/mL) was added, and the cells were incubated for 2 h, followed by removal of the media and addition of 120 µL of DMSO. The samples were then incubated for 15 min with shaking to completely solubilize the formazan. The amount of MTT reduced by cells to its blue formazan derivative was measured spectrophotometrically at 565 nm using a plate reader. Cell survival rates were determined by the comparison of optical density with the corresponding untreated control cell culture. Dose-dependent curves and the corresponding IC_50_ values were calculated using “GraphPad Prism 6” software (GraphPad Software, Inc., San Diego, CA, USA).

#### 3.3.3. Intracellular Accumulation

The studies were carried out according to the protocol described in [83].

#### 3.3.4. Two-Dimensional (2D) Protein Gel Electrophoresis

The 2D protein gel electrophoresis was carried out in accordance with Bio-Rad’s 2D Electrophoresis Guide (https://www.bio-rad.com/webroot/web/pdf/lsr/literature/Bulletin_2651.pdf, accessed on 10 October 2022); for details, see Table S1 (Supplementary Materials).

#### 3.3.5. In Vivo Cytotoxicity Assay

##### Animals

The P388 cell line was subcutaneously (s.c.) injected in BDF1 hybrid mice (21–26 g females, 29–34 g males) obtained from the nursery of the Russian Academy of Medical Sciences “Stolbovaya” [84]. DBA2 mice were used as donors of cancerous mass; a total of 50 animals were recruited. Twenty-three conventional, immunodeficient female mice (BALB/c nude, 20–23 g) were housed in RAMS and used as donors and recipients for HCT116 cells.

##### Compound Formulation

(a) For i.p. administration, the tested compound **29** was dissolved with Pluronic^®^ F-127. The sample contained the compound (17%) and the solubilizing agent (83%). The final 4.8% suspension was prepared at pH = 7.0 in sterile distilled water. (b) For p.o. (per os) administration, we used a 3.4% suspension of the tested compound in 1% starch-based thickener, which was prepared repeatedly ex tempore.

For the evaluation of the compound against leucosis in vivo, we used 5 and 8 passages of P388 culture. Inoculum containing 10^6^. of P388 cells immersed in 0.3 mL of nutrient medium 199 was introduced to the animals subcutaneously in the flank zone.

The first implantation of HCT116 tumor cells (1 × 10^6^ in a volume of 0.5 mL) was performed subcutaneously into two BALB/c nude mice. The first transplantation of the xenograft (50 mg) was performed after 30 days into 2 mice (the 3rd passage was used in vivo). An ampoule containing the tumor suspension (1 mL) obtained from a cryobank was defrosted ex tempore in a thermostat for 10 min at 37 °C. Then, in a laminar flow hood, the tumor suspension (0.5 mL, the first passage) was implanted subcutaneously into the flank area of two BALB/c nude mice (males). After a volume of 0.8–1.0 cm^3^ was achieved, the nudes were passaged using 4 donor mice to obtain the desired grade and mass of the transplants. The donor mice were euthanatized in a CO_2_ chamber, in full accordance with the guidance of the RSPCA and LASA (2015). Tumor nests were accurately isolated, placed in plastic Petri dishes, washed with nutrient medium 199, extracted from capsules, and weighed using an electronic balance. The tumor nests were mechanistically disintegrated to obtain 10% of the tumor samples, and then the nutrient medium was added. The inoculation was performed using individual sterile plastic syringes (1 mL) equipped with 18G suture needles. The transplantation was carried out bilaterally in the flank areas. Each mouse was inoculated with 0.5 mL (10%) of the tumor suspension (40 mg of the tumor mass for each animal). Prior to inoculation, the animals’ skin was treated with 70% alcohol. Between injections, the Petri dish containing the tumor suspension was tightly covered to avoid any contamination. Before injection, the suspension was vigorously stirred in a syringe. After inoculation, the needle was accurately removed to avoid the tumor escaping. The mice were kept under special conditions in an air-conditioned vivarium equipped with a water flask and a feedbox (Cage Type II—hood of open design, EHRET GNBA, Germany). Two mice were used as donors for tumor samples, while twenty-one mice were recruited to perform the trial. The mice were weighed using an electronic balance (MW-T series, USA, graduation mark 0.01 g). The selected mice were assigned to different groups (10 mice per group). One group was marked as a control series and was not treated with the tested compound. The therapeutic effect was assessed considering the standard tumor growth rate (TGRI ≥ 50%) in the case of subcutaneous administration or T/C ≤ 42% (treatment/control).

#### 3.3.6. Acute Toxicity

Outbred mice (males and females) were used in the experiments. Intraperitoneal injections were applied in the posterior third of the abdomen, slightly departing from the midline. The mice were injected with 1 mL of the solution. Compound **29** was dissolved in a low-molecular-weight polyvinylpyrrolidone (Mw = 8000 ± 200) saline infusion solution (hemodesum). The solution was prepared ex tempore. Doses were calculated for each animal separately, according to body weight data.

The obtained data were statistically processed using Microsoft Office Excel 2010 software. The group arithmetic mean (M) and standard error of the mean (m) were calculated. Statistical significance of the differences was assessed using Student’s *t*-test. The differences between these groups were considered significant at *p* < 0.05.

## 4. Conclusions

In this work, a series of novel small-molecule thiohydantoin-based compounds possessing *dispiro*-indolinone scaffolds were readily synthesized and evaluated in vitro and in vivo. Cell-based assays revealed several compounds as potent anticancer agents. The most cytotoxic compounds selectively inhibited the growth of androgen-dependent LNCaP cells, providing IC_50_ values of ~1.2–3.5 μM. Confocal microscopy showed that the product of the intracellular conjugation of propargyl-substituted *dispiro*-indolinone with the fluorescent dye Sulfo-Cy5-azide was mainly localized in the cytoplasm of HEK293 cells. Animal studies with P388-inoculated and xenograft BALB/c nude mice bearing HCT116 tumor cells demonstrated that, at the sample dose of 1000 mg/kg, TGRI > 90% was achieved after i.p. administration. 

On the basis of the performed molecular docking study, MDM2 may be regarded as a possible biological target for the evaluated compounds. However, Western blot data do not confirm this assumption and, apparently, the mechanism of cytotoxic action of the synthesized compounds is different from that of nutlin. Nevertheless, some obtained thiohydantoin-based *dispiro*-indolinones demonstrated significant cytotoxic effects in vitro and certain antitumor properties in vivo, making this class of compounds promising for further optimization and research. Thus, compound **29** demonstrates significant anticancer activity at a daily dose of 1000 mg/kg (i.p., 10 days) in HCT116-bearing xenograft mice, has a therapeutic index (LD_50_/ED_50_) of at least 2.5 in mice, and does not have a cumulative effect.

## Figures and Tables

**Table 1:** Structures and yields of compounds **4**–**69**.

№	R^1^	R^2^	R^3^	Yield, %
**4**	Ph-	Ph-	H	76
**5**	Ph-	Ph-	5-Br	72
**6**	Ph-	Ph-	5-NO_2_	63
**7**	Ph-	Ph-	7-COOH-	61
**8**	Ph-	2-Br-C_6_H_4_-	H	86
**9**	Ph-	2-Cl-C_6_H_4_-	H	87
**10**	Ph-	2-Cl-C_6_H_4_-	5-Br	89
**11**	Ph-	2-Cl-C_6_H_4_-	5-Cl	87
**12**	Ph-	4-Cl-C_6_H_4_-	5-Cl	69
**13**	Ph-	4-Cl-C_6_H_4_-	5-Br	78
**14**	Ph-	3-Cl-C_6_H_4_-	H	57
**15**	Ph-	3-Cl-C_6_H_4_-	5-Br	65
**16**	Ph-	3,4-Cl-C_6_H_3_-	5-Br	61
**17**	Ph-	2-Py-	5-Br	65
**18**	Ph-	2-Py-	5-Cl	53
**19**	Ph-	4-Py-	H-	87
**20**	Ph-	4-Py-	5-Br	89
**21**	Ph-	4-Py-	5-Cl	89
**22**	Ph-	3,4-Cl-C_6_H_3_-	5-Br	61
**23**	Ph-	3-Cl-C_6_H_4_-	5-Cl	68
**24**	Ph-	3-CHO-C_6_H_4_-	5-Cl-	71
**25**	Ph-	cyclo-C_6_H_11_-	H	36
**26**	Ph-	cyclo-C_6_H_11_-	5-Br	35
**27**	4-OC_2_H_5_-C_6_H_4_-	3,4-Cl-C_6_H_3_-	H-	79
**28**	4-OC_2_H_5_-C_6_H_4_-	4-Cl-C_6_H_4_-	H-	62
**29**	4-OC_2_H_5_-C_6_H_4_-	4-Cl-C_6_H_4_-	5-Br	89
**30**	4-OC_2_H_5_-C_6_H_4_-	4-F-C_6_H_4_-	H	72
**31**	4-OC_2_H_5_-C_6_H_4_-	4-F-C_6_H_4_-	5-Br	68
**32**	4-OCH_3_-C_6_H_4_-	3-Cl-C_6_H_4_-	H	59
**33**	4-OCH_3_-C_6_H_4_-	3-Cl-C_6_H_4_-	5-Br	70
**34**	4-OC_2_H_5_-C_6_H_4_-	4-OPropargyl- C_6_H_4_-	5-Br	59
**35**	4-OPropargyl-C_6_H_4_-	4-Cl-C_6_H_4_-	5-Br	94
**36**	4-Cl-C_6_H_4_-	2-Cl-C_6_H_4_-	5-Cl	83
**37**	4-Cl-C_6_H_4_-	4-Cl-C_6_H_4_-	5-Cl	85
**38**	4-Cl-C_6_H_4_-	4-F-C_6_H_4_-	H	85
**39**	4-Cl-C_6_H_4_-	3,4-(OCH_3_)_2_-C_6_H_3_-	5-Cl	81
**40**	4-F-C_6_H_4_-	4-Cl-C_6_H_4_-	5-Br	68
**41**	3-Cl-4-F-C_6_H_3_-	4-OCH_3_-C_6_H_4_-	H	69
**42**	3-Cl-4-F-C_6_H_3_-	4-Cl-C_6_H_4_-	5-Cl	73
**43**	3-Cl-4-F-C_6_H_3_-	3-Cl-C_6_H_4_-	5-Cl	79
**44**	3-Cl-4-F-C_6_H_3_-	3,4-(OCH_3_)_2_-C_6_H_3_-	H	68
**45**	3-Cl-4-F-C_6_H_3_-	3,4-(OCH_3_)_2_-C_6_H_3_-	5-Br	41
**46**	3-Cl-4-F-C_6_H_3_-	4-C_2_H_5_-C_6_H_4_-	H	49
**47**	3-Cl-4-F-C_6_H_3_-	4-C_2_H_5_-C_6_H_4_-	Br	48
**48**	3-Cl-4-F-C_6_H_3_-	4-OCH_3_-C_6_H_4_-	Br	68
**49**	3-Cl-4-F-C_6_H_3_-	4-O(cyclo-C_5_H_9_)-C_6_H_4_-	Br	34
**50**	4-OH-C_6_H_4_-	4-Cl-C_6_H_4_-	H	94
**51**	4-OH-C_6_H_4_-	4-Cl-C_6_H_4_-	5-Br	91
**52**	4-OH-C_6_H_4_-	4-Cl-C_6_H_4_-	5-Cl	88
**53**	4-OH-C_6_H_4_-	2-Cl-C_6_H_4_-	H	75
**54**	4-OH-C_6_H_4_-	2-Cl-C_6_H_4_-	5-Br	76
**55**	4-OH-C_6_H_4_-	2-Cl-C_6_H_4_-	5-Cl	75
**56**	4-OH-C_6_H_4_-	3,4-OCH_3_-C_6_H_3_-	5-Br	69
**57**	4-OH-C_6_H_4_-	3,4-OCH_3_-C_6_H_3_-	5-Cl	53
**58**	4-OCH_3_-3-Cl-C_6_H_3_-	3-Cl-C_6_H_4_-	H	54
**59**	4-OCH_3_-3-Cl-C_6_H_3_-	3-Cl-C_6_H_4_-	5-Br	51
**60**	4-F-C_6_H_4_-	4-OC_2_H_5_-C_6_H_4_-	H	38
**61**	4-F-C_6_H_4_-	4-OC_2_H_5_-C_6_H_4_-	5-Br	36
**62**	CH_3_CHPh-	2-Cl-C_6_H_4_-	5-Br	89
**63**	CH_3_CHPh-	3-Cl-C_6_H_4_-	5-Br	53
**64**	CH_3_CHPh-	2-Cl-C_6_H_4_-	5-Cl	83
**65**	CH_3_CHPh-	4-Cl-C_6_H_4_-	5-Br	65
**66**	CH_3_CHPh-	4-Cl-C_6_H_4_-	5-Cl	62
**67**	Allyl-	2-Py	H	12
**68**	Allyl-	3-Cl-C_6_H_4_-	H	12
**69**	4-OCH_3_-3-Cl-C_6_H_3_-	3-Cl-C_6_H_4_-	1-CH_2_CCH	36

**Table 2:** Cytotoxicity (IC_50_, µM) of compounds **4**–**69**. The data for the most cytotoxic compounds are highlighted in background color.

Compound	Cell Line			
	p53 pos	53 neg	p53 pos	53 neg
	LNCaP	PC3	HCT^wt^	HCT^−/−^
**4**	>100	>100	>100	>100
**5**	2.2 ± 0.8	4.6 ± 2.5	10.5 ± 2.3	12.5 ± 3.2
**6**	>100	>100	>100	>100
**7**	>100	>100	>100	>100
**8**	>100	>100	>100	>100
**9**	>100	>100	21.7 ± 5.8	23.1 ± 6.6
**10**	>100	>100	-	-
**11**	>100	>100	>100	>100
**12**	>100	>100	>100	>100
**13**	1.2 ± 0.6	11.3 ± 1.2	3.5 ± 1.2	>100
**14**	>100	>100	-	-
**15**	>100	>100	>100	>100
**16**	>100	>100	7.6 ± 1.6	8.8 ± 3.5
**17**	>100	>100	5.6 ± 2.3	7.6 ± 3.2
**18**	2.1 ± 0.3	8.2 ± 3.5	60.1 ± 8.6	>100
**19**	>100	>100	>100	>100
**20**	>100	>100	>100	>100
**21**	>100	>100	50.5 ± 10.2	>100
**22**	>100	>100	20.0 ± 8.9	50.0 ± 10.4
**23**	>100	>100	50.7 ± 8.9	50.7 ± 12.6
**24**	>100	>100	>100	>100
**25**	>100	>100	-	-
**26**	>100	>100	-	-
**27**	>100	>100	>100	>100
**28**	>100	>100	>100	>100
**29**	3.5 ± 1.9	9.8 ± 3.7	14.1 ± 4.2	70.5 ± 15.2
**30**	>100	>100	>100	>100
**31**	>100	>100	>100	>100
**32**	>100	>100	>100	>100
**33**	>100	>100	>100	>100
**34**	>100	>100	>100	>100
**35**	>100	>100	>100	>100
**36**	>100	>100	>100	>100
**37**	>100	>100	>100	>100
**38**	>100	>100	>100	>100
**39**	>100	>100	>100	>100
**40**	>100	>100	>100	>100
**41**	>100	>100	>100	>100
**42**	>100	>100	>100	>100
**43**	3.2 ± 1.6	5.7 ± 2.6	9.3 ± 4.6	9.5 ± 4.9
**44**	8.6 ± 1.8	10.2 ± 2.3	35.0 ± 16.2	>100
**45**	>100	>100	-	-
**46**	>100	>100	6.3 ± 2.5	6.5 ± 3.6
**47**	>100	>100	>100	>100
**48**	>100	>100	>100	>100
**49**	>100	>100	-	-
**50**	18.0 ± 4.7	4.65 ± 2.3	6.9 ± 3.1	>100
**51**	>100	>100	>100	>100
**52**	>100	>100	>100	>100
**53**	>100	>100	>100	>100
**54**	9.5 ± 2.5	20.0 ± 8.4	>100	>100
**55**	7.0 ± 1.0	15.1 ± 1.2	>100	>100
**56**	9.0 ± 2.0	30.0 ± 10.5	>100	>100
**57**	17.0 ± 1.0	20.0 ± 6.9	>100	>100
**58**	>100	>100	-	-
**59**	>50	>50	-	-
**60**	>100	>100	-	-
**61**	>100	>100	-	-
**62**	>100	>100	>100	>100
**63**	3.4 ± 1.5	8.8 ± 3.1	8.0 ± 2.5	>100
**64**	>100	>100	24.0 ± 6.7	>100
**65**	>100	>100	>100	>100
**66**	>100	>100	>100	>100
**67**	>50	>50	-	-
**68**	>50	>50	-	-
**69**	>100	>100	>100	>100
**Nutlin-3a**	2.7 ± 0.9	28.9 ± 3.7	4.5 ± 1.3	>100

## Data Availability

Not applicable.

## Supplementary Materials

The following supporting information can be downloaded at: https://www.mdpi.com/article/10.3390/molecules28031325/s1, General procedure for the synthesis of compounds, NMR spectra, Figure S1: ^1^H NMR spectrum of dispiro-indolinone 27, Figure S2: ^1^H-^1^H COSY NMR spectrum of dispiro-indolinone 27, Figure S3: ^1^H-^13^C HSQC spectrum of dispiro-indolinone 27, Figure S4: ^1^H-^13^C HMBC spectrum of dispiro-indolinone 27, Figure S5: The mechanism of cell death (necrosis/apoptosis) caused by compound 12 by flow cytometry staining with 7AAD/AnnexinV, Figure S6: Representative protein gels after 2D electrophoresis, Table S1: Proteins with changed level, determined from 2D-difference gel electrophoresis, Table S2: Anticancer effect of compound 29 achieved after p.o. dose of 170 mg/kg (o.d., 10 days) in P388-inoculated mice, Table S3: Acute toxicity study design for compound 29, Table S4: Dynamics of body weight of mice in the study of acute toxicity of compound 29, Table S5: Average group indicators of the relative mass of the internal organs of mice in the study of acute toxicity of the compound **29**. References [85,86] are cited in Supplementary Materials.
