# Supplementary material for: Synthesis and Biological Evaluation of Novel Dispiro-Indolinones with Anticancer Activity
Source: Molecules. 2023 Jan 30;28(3):1325. doi: 10.3390/molecules28031325 (PMC9919490; doi:10.3390/molecules28031325)
Supplement: Supplementary file 1 [file molecules-28-01325-s001.zip › molecules-2059554-supplementary.pdf]

Synthesis and Biological Evaluation of Novel Dispiroindolinones with Anticancer Activity

Yan A. Ivanenkov[1,2], Maxim E. Kukushkin[1], Anastasia A. Beloglazkina[1], Radik R. Shafikov [1], Alexander A. Barashkin[1], Andrey A. Ayginin[1], Marina S. Serebrykova[1], Alexander G. Majouga[3], Dmitry A Skvortsov[1], Viktor A. Tafeenko[1] and Elena K. Beloglazkina[1*]

[1] Moscow State University, Chemistry Dept., 119991, Moscow, Leninskie gory, building 1/3, GSP-1, Russia; yaiivanenkov@gmail.com (Y.A.I.); lemmingg@mail.ru (M.E.K.), anastas-beloglaz@mail.ru (An.A.B.); iltarn@mail.ru (R.R.S.); aleksandr.baraskin@gmail.com (Al.A.B.); ayginin75@gmail.com (A.A.A); mserebr@mail.ru (M.S.S.); majouga@org.chem.msu.ru (A.G.M.); skvorratd@mail.ru (D.A.S.); tafeenko-victor@yandex.ru (V.A.T.); beloglazki@mail.ru (E.K.B.)
[2] The Federal State Unitary Enterprise Dukhov Automatics Research Institute (VNIIA), 22, ul. Sushchevskaya, Moscow 127055, Russia, mail: Moscow Post Office, PO box 918, Moscow 101000, Russia, phone: +7 (499) 978-7803, fax: +7 (499) 978-0903, 978-0578; yaiivanenkov@gmail.com (Y.A.I.)
[3] National University of Science and Technology MISiS, Moscow, 119049, Russian Federation; majouga@org.chem.msu.ru (A.G.M.)
* Correspondence: beloglazki@mail.ru

Supplementary Information

Commercially available chemicals were purchased from Sigma Aldrich. ^{1}H NMR spectra of all synthesized compounds were recorded on Bruker Advance 400 spectrometer at 400 MHz in DMSO-d_6. Chemical shifts (δ) are reported in parts per million (ppm) downfield from TMS and referenced from solvent references. Coupling constants are reported in *Hz*.

5-Arylidene-2-thiohydantoins were synthesized using the procedures described in [1] and [2]. Sarcosine **2** and isatines **3** are commercially available.

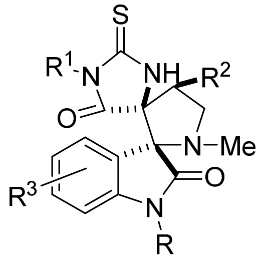

General procedure for the synthesis of compounds 4-74

Isatin **3** (0.3 mmol) was added to a refluxing solution of 5-arylidene-2-thiohydantoin **1** (0.3 mmol) and sarcosine **2** (0.6 mmol) in ethanol (10–15 mL) and the resulting mixture was boiled for ~6 hours, monitoring the completion of the reaction by TLC. After cooling, the reaction mixture was poured into 100 ml of cold water, the precipitate that formed was filtered off and recrystallized from ethanol.

1-phenyl-4'-phenyl-1'-methyl-2-thioxodispiro[imidazolidine-4,3'-pyrrolidine-2',3''-indoline]-2'',5-dione (4). Yield 76%, white solid. M.p. 211-212 ºC. ^{1}H NMR (400 MHz, DMSO-d6, δ, ppm):

10.65 (s, 1H, NH-indolinone), 10.50 (s, 1H, NH-imidazolidine), 7.27-7.11 (m, 13H, aryl), 7.05 (s, 1H), 4.15 (m, 1H, pyrrolidine), 4.01 (m, 1H, pyrrolidine), 3.32 (m, 1H, pyrrolidine), 2.15 (s, 3H, NCH_3). HRMS (ESI, m/z): calculated 455.1528 ($C_{26}H_{22}N_4O_2S$, $[M+H]^+$), found 455.1530.

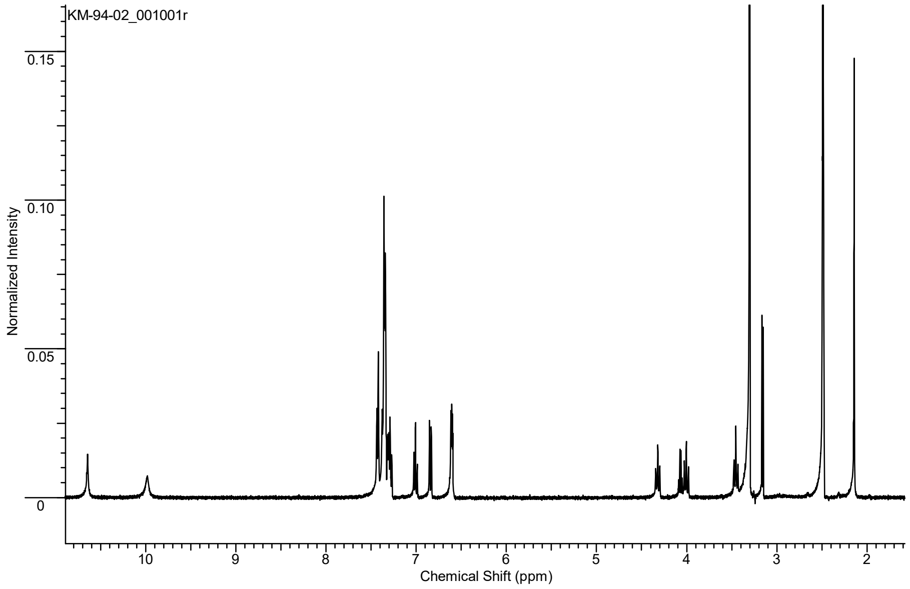

Figure S1. ^{1}H NMR spectra of compound **4**.

1-phenyl-4'-phenyl-1'-methyl-5'''-bromo-2-thioxodispiro[imidazolidine-4,3'-pyrrolidine-2',3'-indoline]-2'',5-dione (5). Yield 72%, white solid. M.p. 153-155 °C .^{1}H NMR (400 MHz, DMSO-d6, δ, ppm): 10.79 (s, 1H, NH-indolinone), 10.32 (s, 1H, NH-imidazolidine), 7.54-7.27 (m, 10H, aryl), 6.82 (d, J=7.4 Hz, 1H, aryl), 6.66 (m, 2H, aryl), 4.32 (t, J=9.1 Hz, 1H, pyrrolidine), 3.98 (t, J=9.1 Hz, 1H, pyrrolidine), 3.49 (t, J=9.1 Hz, 1H, pyrrolidine), 2.18 (s, 3H, NCH_3). HRMS (ESI, m/z): calculated 535.0622 ($C_{26}H_{21}BrN_4O_2S$, $[M+H]^+$), found 535.0626.

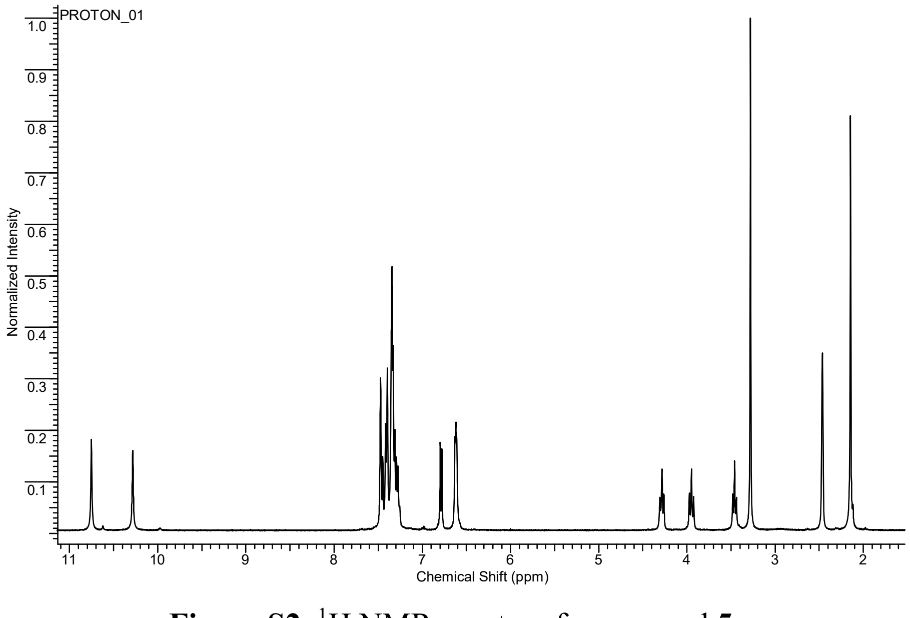

Figure S2. ^{1}H NMR spectra of compound **5**.

1-phenyl-4'-phenyl-1'-methyl-5'''-nitro-2-thiooxodispiro[imidazolidine-4,3'-pyrrolidine-2',3''-indoline]-2'',5-dione (6). Yield 63%, white solid. M.p. 245-248 °C. ^{1}H NMR (400 MHz, DMSO-d6, δ, ppm): 11.36 (s, 1H, NH-indolinone), 10.63 (s, 1H, NH-imidazolidine), 8.27 (d, J=6.4 Hz, 1H, aryl), 8.19 (d, J=7.1 Hz, 1H, aryl), 7.45-7.28 (m, 8H, aryl), 7.04 (d, J=8.7 Hz, 1H, aryl), 6.65-6.58 (m, 2H, aryl), 4.35 (t, J=9.1 Hz, 1H, pyrrolidine), 4.00 (t, J=9.1 Hz, 1H, pyrrolidine), 3.55 (t, J=9.1 Hz, 1H, pyrrolidine), 2.21 (s, 3H, NCH_3). HRMS (ESI, m/z): calculated 500.1387 ($C_{26}H_{21}N_5O_4S$, $[M+H]^+$), found 500.1390.

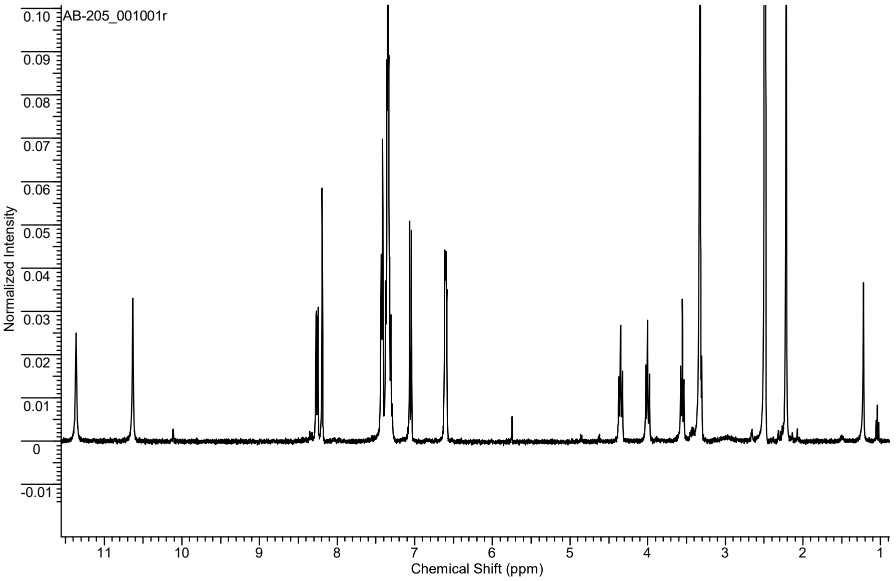

Figure S3. ^{1}H NMR spectra of compound **6**.

1-phenyl-4'-phenyl-1'-methyl-7'''-carboxy-2-thiooxodispiro[imidazolidine-4,3'-pyrrolidine-2',3''-indoline]-2'',5- dione (7). Yield 61%, white solid. M.p. 226-227 °C. ^{1}H NMR (400 MHz, DMSO-d6, δ, ppm): 10.52 (s, 1H, NH-indolinone), 10.42 (s, 1H, NH-imidazolidine), 7.96 (d, J=7.2 Hz, 1H, aryl), 7.82 (t, J=8.1 Hz, 2H, aryl), 7.45-7.24 (m, 7H, aryl), 7.05-6.93 (m, 2H, aryl), 6.59 (d, J=3.8 Hz, 1H, Ph), 4.33 (t, J=8.8 Hz, 1H, pyrrolidine), 4.02 (t, J=8.8 Hz, 1H, pyrrolidine), 3.46 (t, J=8.8 Hz, 1H, pyrrolidine), 2.14 (s, 3H, NCH_3). HRMS (ESI, m/z): calculated 499.1435 ($C_{27}H_{23}N_4O_4S$, $[M+H]^+$), found 499.1438.

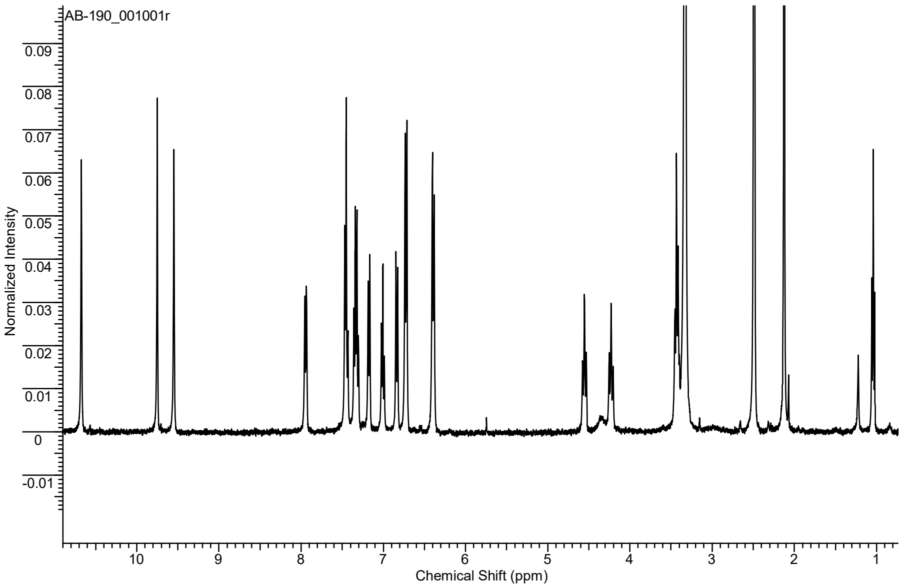

Figure S4. ^{1}H NMR spectra of compound **7**.

1-phenyl-4'-(2-bromophenyl)-1'-methyl-2-thioxodispiro[imidazolidine-4,3'-pyrrolidine-2',3''-indoline]-2'',5-dione (8). Yield 86%, white solid. M.p. 229-230 °C. ^{1}H NMR (400 MHz, DMSO-d6, δ, ppm): 10.71 (s, 1H, NH-indolinone), 9.74 (s, NH-imidazolidine), 8.05 (dd, J_1=1.2 Hz, J_2=6.4 Hz, 1H, aryl), 7.65 (dd, J_1=1.1 Hz, J_2=6.9 Hz, 1H, aryl), 7.51 (t, J=7.6 Hz, 1H, aryl), 7.44-7.38 (m, 3H, aryl), 7.35 (dt, J_1=1.1 Hz, J_2=7.7 Hz, 1H, aryl), 7.28 (dt, J_1=1.1 Hz, J_2=7.5 Hz, 1H, aryl), 7.20 (d, J=7.0 Hz, 1H, aryl), 7.03 (dt, J_1=0.9 Hz, J_2=7.6 Hz, 1H , aryl), 6.88 (d, J=7.6 Hz, 1H, aryl), 6.71-6.65 (m, 2H, aryl), 4.61 (t, J=8.6 Hz, 1H, pyrrolidine), 4.20 (t , J=8.6 Hz, 1H, pyrrolidine), 3.49 (t, J=8.6 Hz, 1H, pyrrolidine), 2.14 (s, 3H, NCH_3). HRMS (ESI, m/z): calculated 533.0641 ($C_{26}H_{21}BrN_4O_2S$, $[M+H]^+$), found 533.0644.

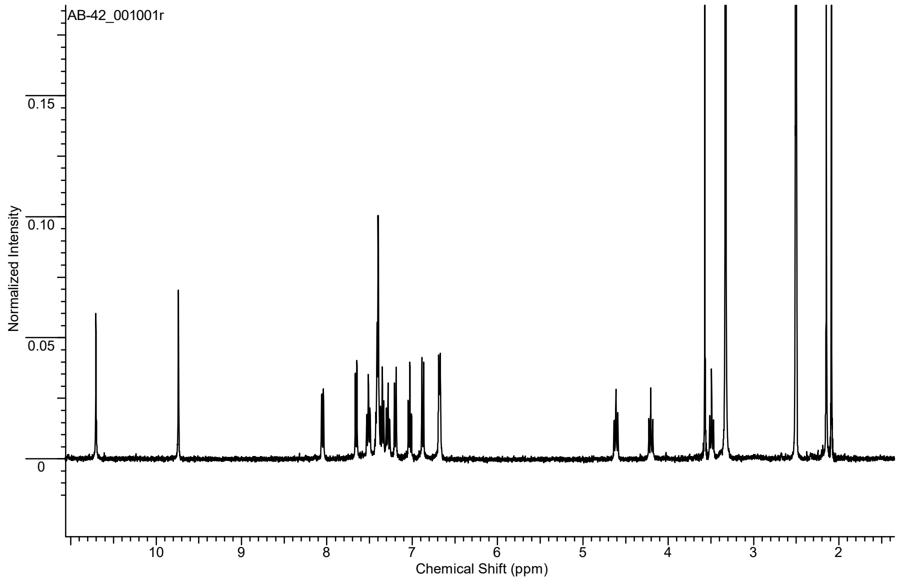

Figure S5. ^{1}H NMR spectra of compound **8**.

1-phenyl-4'-(2-chlorophenyl)-1'-methyl-2-thioxodispiro[imidazolidine-4,3'-pyrrolidine-2',3"-indoline]-2",5-dione (9). Yield 87%, white solid. M.p. 242-244 °C. ^{1}H NMR (400 MHz, DMSO-d6, δ, ppm): 10.70 (s, 1H, NH-indolinone), 9.74 (s, 1H, NH-imidazolidine), 7.97 (dd, J_1=0.9 Hz, J_2=6.5 Hz, 1H, aryl), 7.51-7.32 (m, 9H, aryl), 7.21 (d, J=7.3 Hz, 1H), 7.03 (dt, J_1=0.9 Hz, J_2=7.7 Hz, 1H, aryl), 6.87 (d, J=7.7 Hz, 1H, aryl), 4.60 (t, J=9.1 Hz, 1H, pyrrolidine), 4.27 (t, J=9.1 Hz, 1H, pyrrolidine), 3.47 (t, J=9.1 Hz, 1H, pyrrolidine), 2.15 (s, 3H, NCH_3). HRMS (ESI, m/z): calculated 489.1147 ($C_{26}H_{22}ClN_4O_2S$, $[M+H]^+$), found 489.1150.

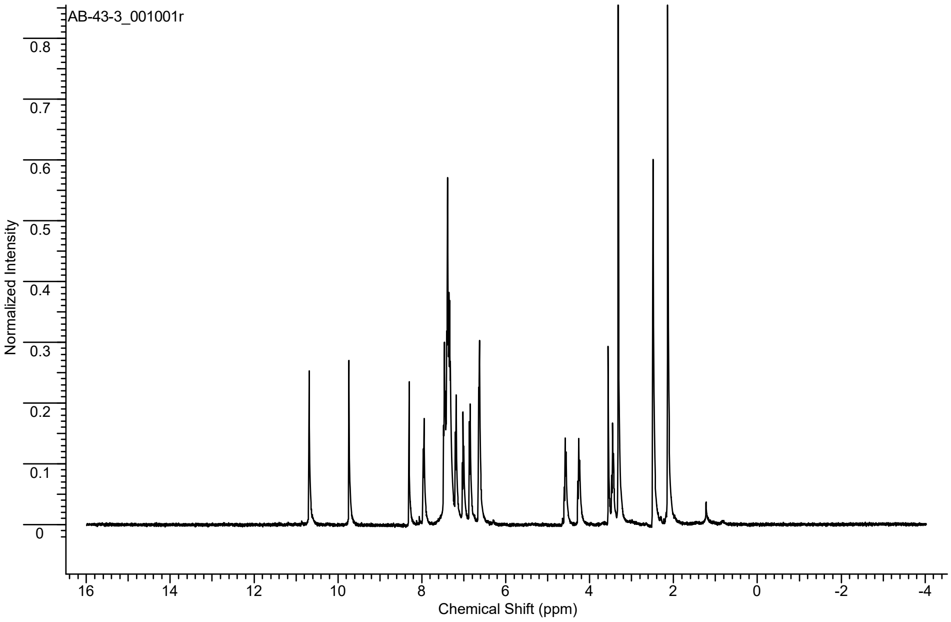

Figure S6. ^{1}H NMR spectra of compound **9**.

1-phenyl-4'-(2-chlorophenyl)-1'-methyl-5'''-bromo-2-thiooxodispiro[imidazolidine-4,3'-pyrrolidine-2',3"- indoline]-2",5-dione (10). Yield 89%, white solid. M.p. 209-210 °C. ^{1}H NMR (400 MHz, DMSO-d6, δ, ppm):10.86 (s, 1H, NH-indolinone), 10.02 (s, 1H, NH-imidazolidine), 7.95 (d, J=7.4 Hz, 1H, aryl), 7.56 (dd, J_1=2.0 Hz, J_2=8.2 Hz, 1H, aryl), 7.51-7.41 (m, 4H, aryl), 7.37 (td, J_1=1.0 Hz, J_2=7.8 Hz, 1H, aryl), 7.27 (d, J=2.0 Hz, 1H, aryl), 6.84 (d, J=8.2 Hz, 1H, aryl), 6.72-6.66 (m, 2H, aryl), 4.56 (t, J=8.9 Hz, 1H, pyrrolidine), 4.25 (t, J=9.1 Hz, 1H, pyrrolidine), 3.48 (t, J=8.5 Hz, 1H, pyrrolidine), 2.17 (s, 3H, NCH_3). HRMS (ESI, m/z): calculated 569.0219 ($C_{26}H_{20}BrClN_4O_2S$ $[M+H]^+$), found 569.0228.

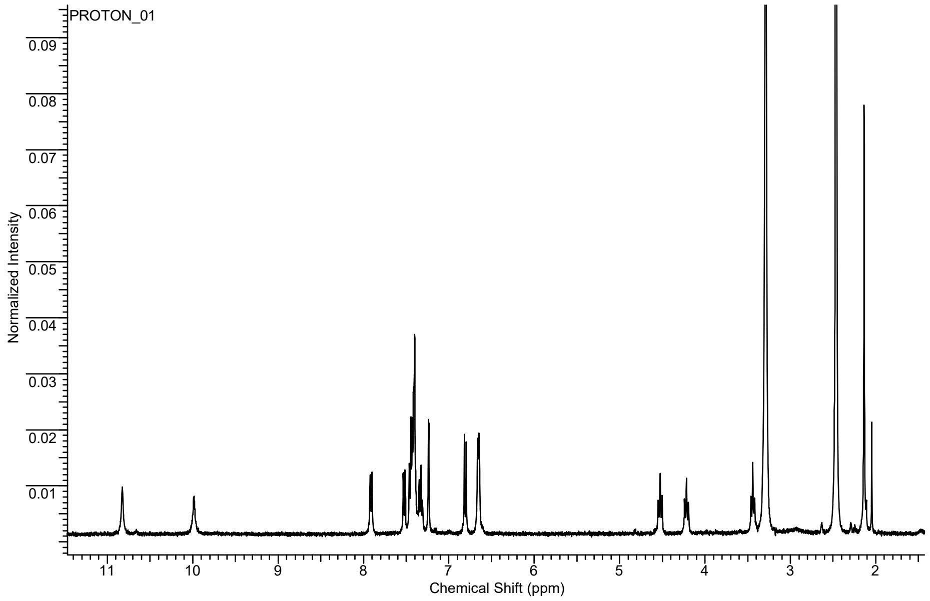

Figure S7. ^{1}H NMR spectra of compound **10**.

1-phenyl-4'-(2-chlorophenyl)-1'-methyl-5'''-chloro-2-thioxodispiro[imidazolidine-4,3'-pyrrolidine-2',3''-indoline]-2'',5-dione (11). Yield 87%, white solid. M.p. 201-202 ºC. ^{1}H NMR (400 MHz, DMSO-d6, δ, ppm):10.85 (s, 1H, NH-indolinone), 9.97 (s, 1H, NH-imidazolidine), 7.95 (d, J=7.7 Hz, 1H, aryl), 7.54-7.40 (m, 6H, aryl), 7.37 (m, 1H, aryl), 7.15 (s, 1H, aryl), 6.89 (d, J =7.7 Hz, 1H, aryl), 6.71-6.65 (m, 2H, aryl), 4.58 (t, J=9.1 Hz, 1H, pyrrolidine), 4.25 (t, J=9.1 Hz, 1H, pyrrolidine), 3.48 (t, J=8.2 Hz, 1H, pyrrolidine), 2.17 (s, 3H, NCH_3). HRMS (ESI, m/z): calculated 522.0744 ($C_{26}H_{20}Cl_2N_4O_2S$, $[M+H]^+$), found 522.0749.

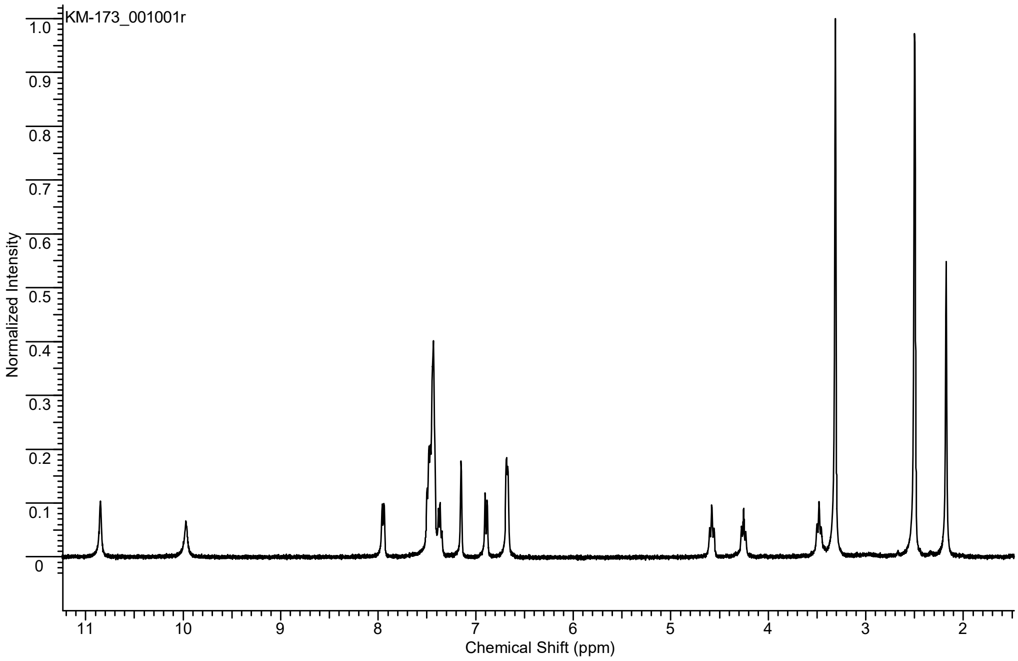

Figure S8. ^{1}H NMR spectra of compound **11**.

1-phenyl-4'-(4-chlorophenyl)-1'-methyl-2-thioxodispiro[imidazolidine-4,3'-pyrrolidine-2',3"-indoline]-2",5-dione (12). Yield 69%, white solid. M.p. 210-211 ºC. ^{1}H NMR (400 MHz, DMSO-d6, δ, ppm):10.80 (s, 1H, NH-indolinone), 10.45 (s, 1H, NH-imidazolidine), 7.48 (d, J=8.3 Hz, 2H, aryl), 7.45-7.34 (m, 7H, aryl), 6.87 (d, J =8.2 Hz, 1H, aryl), 6.71-6.64 (m, 2H, aryl), 4.34 (t, J=9.1 Hz, 1H, pyrrolidine), 3.94 (t, J=9.4 Hz, 1H, pyrrolidine), 3.50 (t, J=8.9 Hz, 1H, pyrrolidine), 2.17 (s, 3H, NCH_3). HRMS (ESI, m/z): calculated 522.0744 ($C_{26}H_{20}Cl_2N_4O_2S$, $[M+H]^+$), found 522.0751.

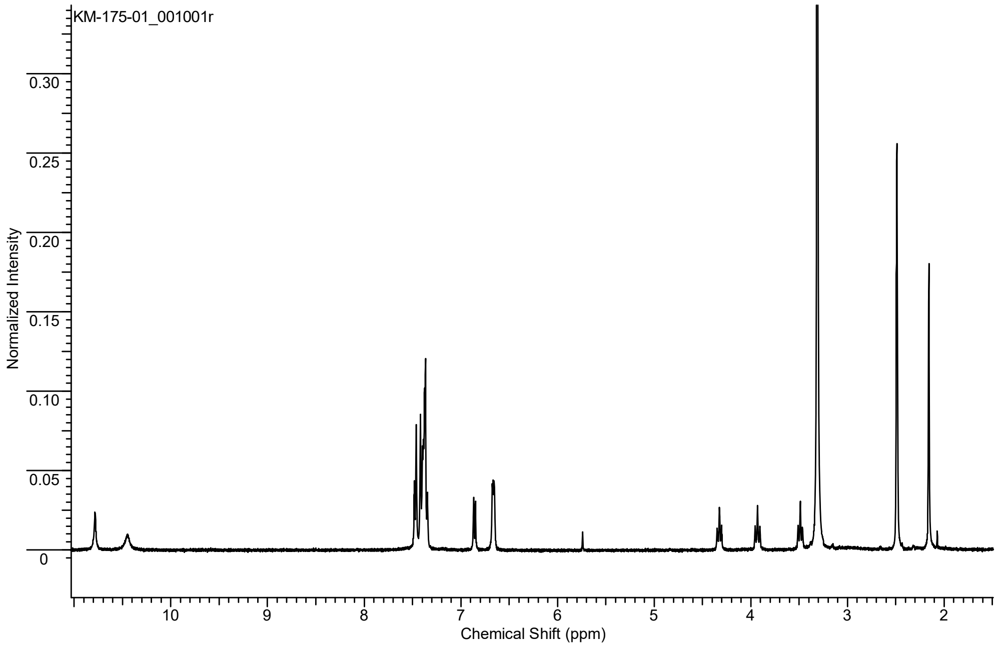

Figure S9. ^{1}H NMR spectra of compound **12**.

1-phenyl-4'-(4-chlorophenyl)-1'-methyl-5'''-bromo-2-thiooxodispiro[imidazolidine-4,3'-pyrrolidine-2',3"-indoline]-2 ",5-dione (13). Yield 78%, white solid. M.p. 251-252 ºC. ^{1}H NMR (400 MHz, DMSO-d6, δ, ppm): 10.80 (s, 1H, NH-indolinone), 10.35 (s, 1H, NH-imidazolidine), 7.54-7.31 (m, 10H, aryl), 6.83 (d, J=8.6 Hz, 1H, aryl), 6.68-6.64 (m, 2H, aryl), 4.32 (t, J=9.2 Hz, 1H, pyrrolidine), 3.99 (t, J=9.2 Hz, 1H, pyrrolidine), 3.50 (t, J=9.2 Hz , 1H, pyrrolidine), 2.19 (s, 3H, NCH_3). HRMS (ESI, m/z): calculated 566.0244 ($C_{26}H_{21}BrClN_4O_2S$, $[M+H]^+$), found 566.0246.

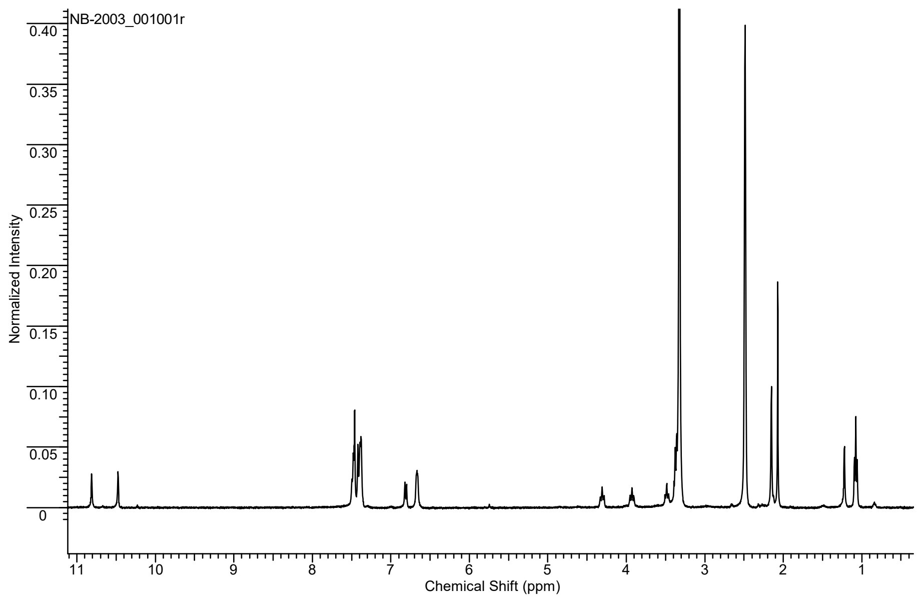

Figure S10. ^{1}H NMR spectra of compound **13**.

1-phenyl-4'-(3-chlorophenyl)-1'-methyl-2-thiooxodispiro[imidazolidine-4,3'-pyrrolidine-2',3"-indoline]-2",5-dione (14). Yield 57%, white solid. M.p. 253-255 °C. ^{1}H NMR (400 MHz, DMSO-d6, δ, ppm): 10.66 (s, 1H, NH-indolinone), 10.29 (s, 1H, NH-imidazolidine), 7.55 (s, 1H, aryl), 7.41-7.35 (m, 6H, aryl), 7.34-7.28 (m, 2H, aryl), 7.01 (td, J_1=0.7 Hz, J_2=7.5 Hz, 1H, aryl), 6.86 (d, J=7.6 Hz, 1H, aryl), 6.67-6.62 (m, 2H, aryl), 4.33 (t, J=9.0 Hz, 1H, pyrrolidine), 3.98 (t, J=9.4 Hz, 1H, pyrrolidine), 3.47 (t, J=8.6 Hz, 1H, pyrrolidine), 2.14 (s, 3H, NCH_3). HRMS (ESI, m/z): calculated 489.1147 ($C_{26}H_{21}ClN_4O_2S$ $[M+H]^+$), found 489.1139.

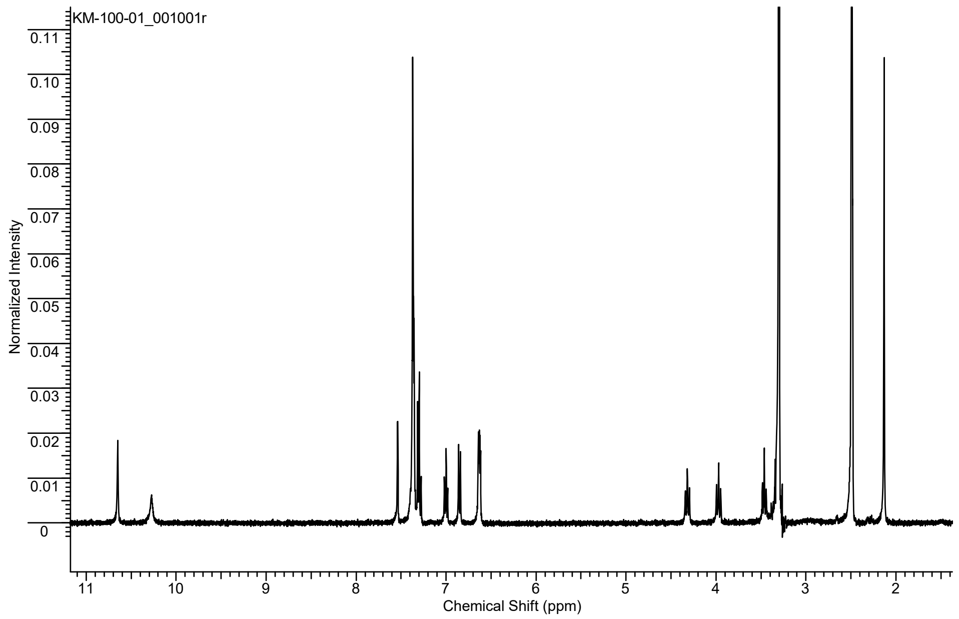

Figure S11. ^{1}H NMR spectra of compound **14**.

1-phenyl-4’-(3-chlorophenyl)-1’-methyl-5'''-bromo-2-thiooxodispiro[imidazolidine-4,3’-pyrrolidine-2’,3”-indoline]-2”,5-dione (15). Yield 65%, white solid. M.p. 221-222 ºC. ^{1}H NMR (400 MHz, $CDCl_3$, δ, ppm): 9.69 (s, 1H, NH-indolinone), 9.06 (s, 1H, NH-imidazolidine), 7.67 (s, 1H, aryl), 7.64 (s, 1H, aryl), 7.48-7.41 (m, 4H, aryl), 7.41-7.35 (m, 4H, aryl), 6.83-6.77 (m, 2H, aryl), 6.66 (d, J=8.2 Hz, 1H, aryl), 7.49-7.41 (m, 2H, aryl), 4.50-4.40 (m, 2H, pyrrolidine), 3.60 (m, 1H, pyrrolidine), 2.15 (s, 3H, NCH_3). HRMS (ESI, m/z): calculated 569.0219 ($C_{26}H_{20}BrClN_4O_2S$ $[M+H]^+$), found 569.0231.

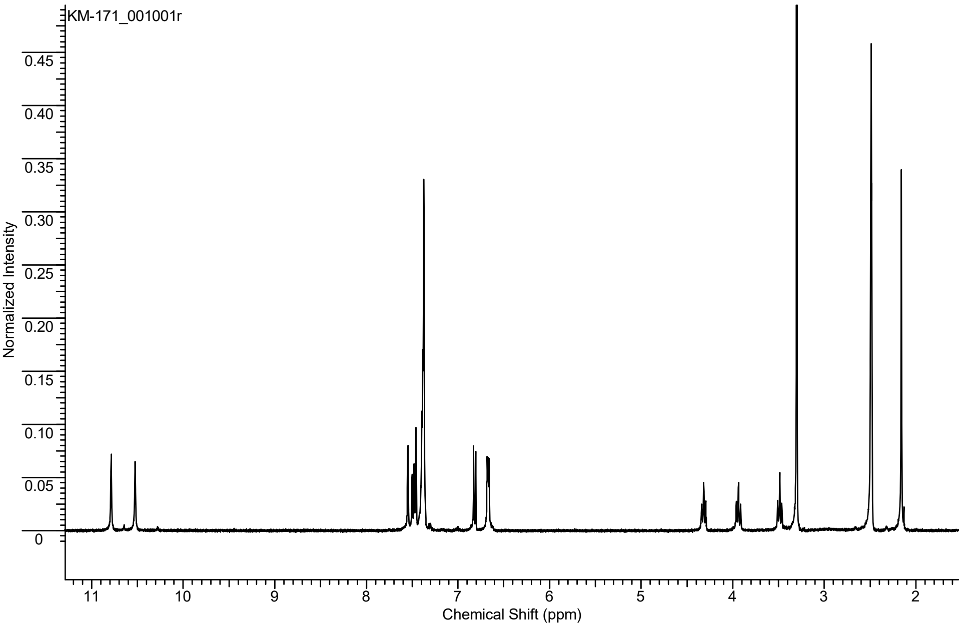

Figure S12. ^{1}H NMR spectra of compound **15**.

1-phenyl-4'-(3,4-dichlorophenyl)-1'-methyl-5'''-bromo-2-thiooxodispiro[imidazolidine-4,3’-pyrrolidine-2',3''-indoline]-2”,5-dione (16). Yield 61%, white solid. M.p. 215-216 ºC. ^{1}H NMR (400 MHz, DMSO-d6, δ, ppm): 10.82 (s, 1H, NH-indolinone), 10.64 (s, 1H, NH-imidazolidine), 7.80 (s, 1H, aryl), 7.61 (d, J=8.2 Hz, 1H, aryl), 7.46-7.34 (m, 5H, aryl), 7.32 (d, J=2.0 Hz, 1H, aryl), 6.88 (d, J=8.2, 1H, aryl), 6.73-6.66 (m, 2H, aryl), 4.34 (t, J=9.0 Hz, 1H, pyrrolidine), 3.92 (t, J=9.0 Hz, 1H, pyrrolidine), 3.51 (t, J =9.0 Hz, 1H, pyrrolidine), 2.16 (s, 3H, NCH_3). HRMS (ESI, m/z): calculated 600.9862 ($C_{26}H_{19}BrCl_2N_4O_2S$, $[M+H]^+$), found 600.9869.

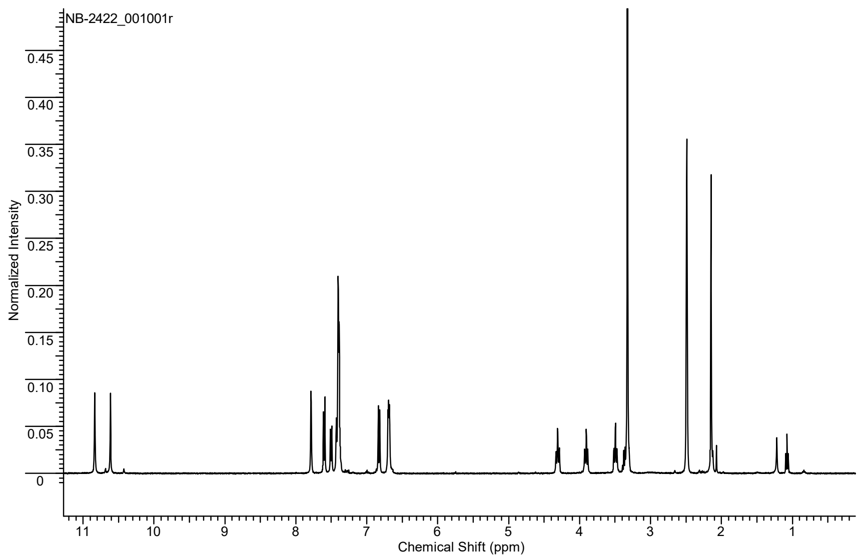

Figure S13. ^{1}H NMR spectra of compound **16**.

1-phenyl-4'-(2-pyridinyl)-1'-methyl-5'''-bromo-2-thiooxodispiro[imidazolidine-4,3'-pyrrolidine-2',3''-indoline]-2'',5-dione (17) [2]. Yield 65%, white solid. M.p. 208-209 ºC. ^{1}H NMR (400 MHz, DMSO-d6, δ, ppm): 10.72 (s, 1H, NH-indolinone), 10.22 (s, 1H, NH-imidazolidine), 8.50 (s, 1H, aryl), 7.83 (t, J=7.0 Hz, 1H, aryl), 7.46-7.40 (m, 6H, aryl), 7.29 (bs, 1H, aryl), 6.91 (d, J=7.0 Hz, 2H, aryl), 6.78 (d, J=8.0 Hz, 1H, aryl), 4.54 (t, J=9.6 Hz, 1H, pyrrolidine), 4.05 (t, J=9.6 Hz, 1H, pyrrolidine), 3.61 (t, J=9.6 Hz, 1H, pyrrolidine), 2.20 (s, 3H, NCH_3). HRMS (ESI, m/z): calculated 533.0471 ($C_{25}H_{20}BrN_5O_2S$, $[M+H]^+$), found 533.0475.

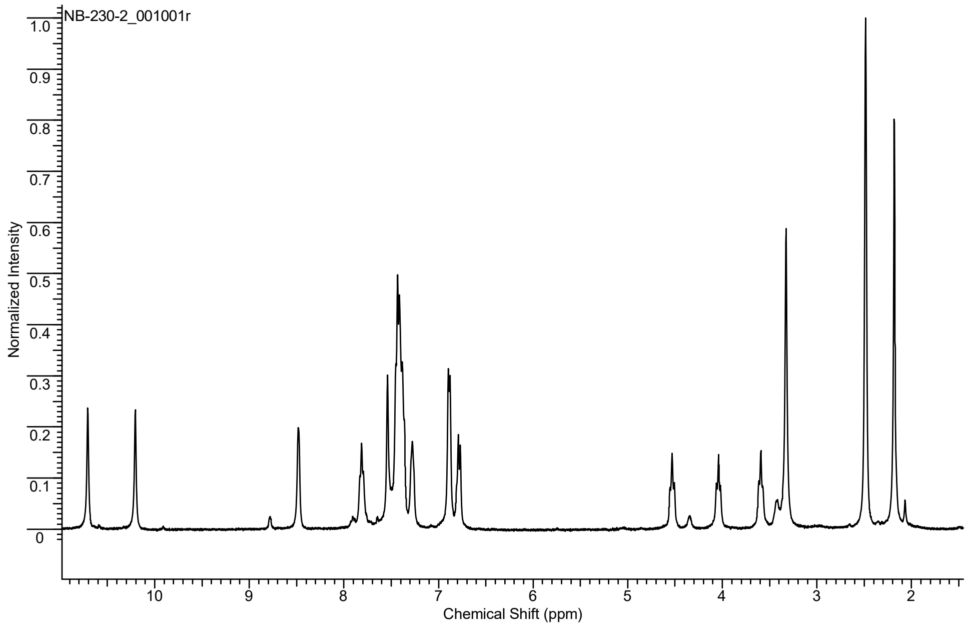

Figure S14. ^{1}H NMR spectra of compound **17**.

1-phenyl-4'-(2-pyridinyl)-1'-methyl-5'''-chloro-2-thiooxodispiro[imidazolidine-4,3'-pyrrolidine-2',3''-indoline]-2'',5-dione (18). Yield 53%, white solid. M.p. 215-217 ºC. ^{1}H NMR (400

MHz, DMSO-d6, δ, ppm): 10.71 (s, 1H, NH-indolinone), 10.21 (s, 1H, NH-imidazolidine), 8.50 (s, 1H, aryl), 7.83 (t, J=7.4 Hz, 1H, aryl), 7.45-7.37 (m, 6H, aryl), 7.34-7.29 (m, 2H, aryl), 6.90 (d, J=6.9 Hz, 1H, aryl), 6.84 (d, J=8.0 Hz, 1H, aryl), 4.55 (t, J=8.9 Hz, 1H, pyrrolidine), 4.06 (t, J=8.9 Hz, 1H, pyrrolidine), 3.61 (t, J=8.9 Hz, 1H, pyrrolidine), 2.20 (s, 3H, NCH_3).

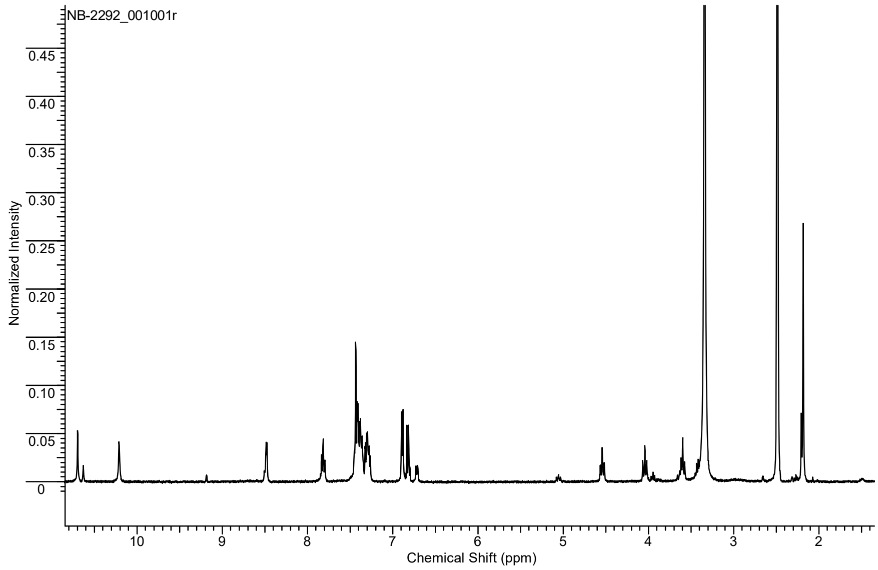

Figure S15. ^{1}H NMR spectra of compound **18**.

1-phenyl-4'-(4-pyridinyl)-1'-methyl--2-thioxodispiro[imidazolidine-4,3'-pyrrolidine-2',3"-indoline]-2",5-dione (19). Yield 87%, white solid. M.p. 222-223 ºC. ^{1}H NMR (400 MHz, DMSO-d6, δ, ppm): 10.69 (s, 1H, NH-indolinone), 10.35 (s, 1H, NH-imidazolidine), 8.54 (d, J=6.5 Hz, 2H, aryl), 7.46-7.28 (m, 6H, aryl), 7.00 (t, J=8.2 Hz, 1H, aryl), 6.84 (d, J=7.46 Hz, 1H, aryl), 6.68-6.63 (m, 3H, aryl), 4.30 (t, J=8.8 Hz, 1H, pyrrolidine), 4.02 (t, J=8.8 Hz, 1H, pyrrolidine), 3.48 (t, J=8.8 Hz, 1H, pyrrolidine), 2.14 (s, 3H, NCH_3). HRMS (ESI, m/z): calculated 456.489 ($C_{25}H_{21}N_5O_2S$, $[M+H]^+$), found 456.1491.

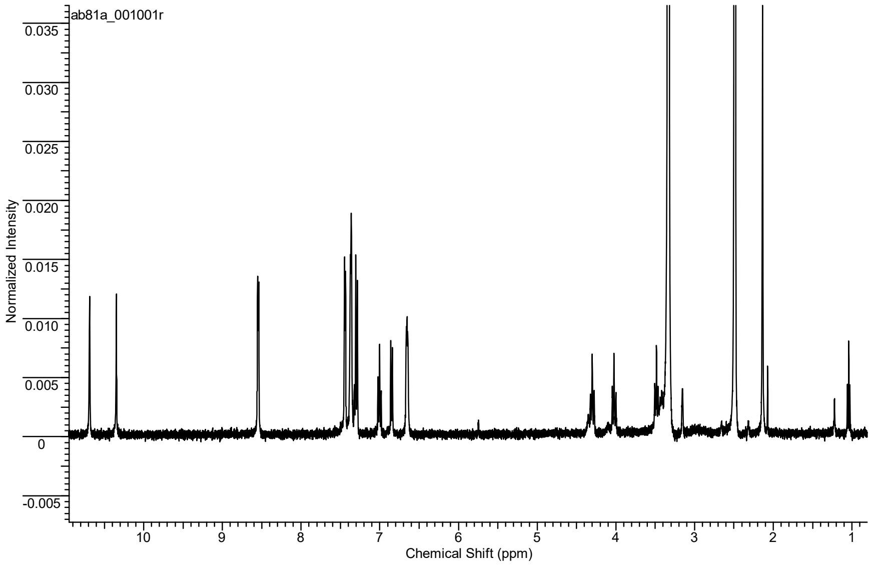

Figure S16. ^{1}H NMR spectra of compound **19**.

1-phenyl-4'-(4-pyridinyl)-1'-methyl-5'''-bromo-2-thiooxodispiro[imidazolidine-4,3'-pyrrolidine-2',3''-indoline]-2'',5-dione (20). Yield 89%, white solid. M.p. 215-216 ºC. ^{1}H NMR (400 MHz, DMSO-d6, δ, ppm): 10.84 (s, 1H, NH-indolinone), 10.56 (s, 1H, NH-imidazolidine), 8.55 (d, J=4.9 Hz, 2H, aryl), 7.54-7.36 (m, 6H, aryl), 6.85-6.67 (m, 4H, aryl), 4.30 (t, J=8.8 Hz, 1H, pyrrolidine), 3.99 (t, J =8.8 Hz, 1H, pyrrolidine), 3.31 (t, J=8.8 Hz, 1H, pyrrolidine), 2.16 (s, 3H, NCH_3). HRMS (ESI, m/z): calculated 534.0594 ($C_{25}H_{20}BrN_5O_2S$, $[M+H]^+$), found 534.0603.

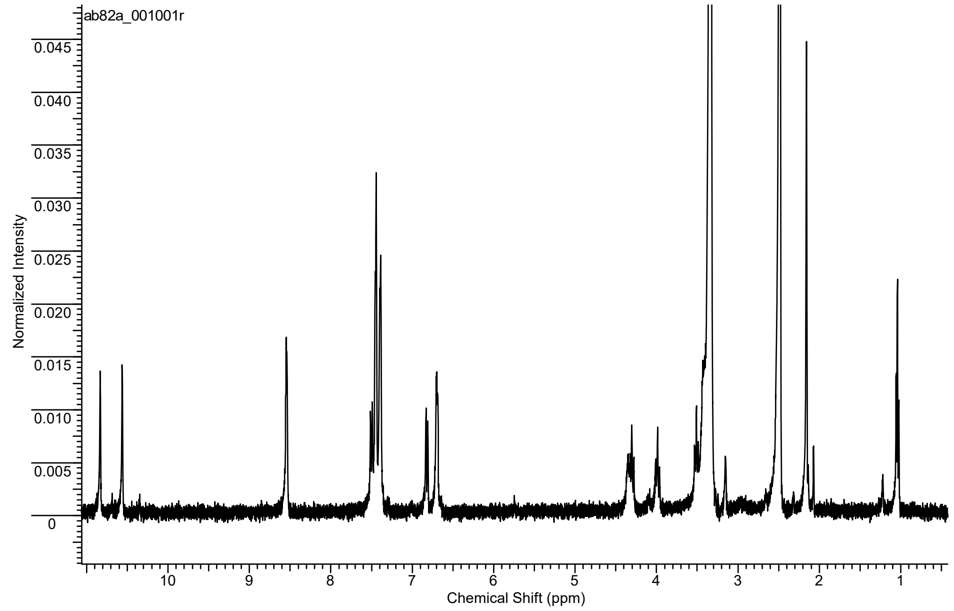

Figure S17. ^{1}H NMR spectra of compound **20**.

1-phenyl-4'-(4-pyridinyl)-1'-methyl-5'''-chloro-2-thiooxodispiro[imidazolidine-4,3'-pyrrolidine-2',3''-indoline]-2'',5-dione (21). Yield 89%, white solid. M.p. 227-228 ºC. ^{1}H NMR (400 MHz, DMSO-d6, δ, ppm): 10.82 (s, 1H, NH-indolinone), 10.58 (s, 1H, NH-imidazolidine), 8.55 (d, J=4.9 Hz, 2H, aryl), 7.47-7.32 (m, 7H, aryl), 6.87 (d, J=8.2 Hz, 1H, aryl), 6.72-6.67 (m, 2H, aryl), 4.32 (t, J=8.9 Hz, 1H, pyrrolidine), 3.99 (t, J=8.9 Hz, 1H, pyrrolidine), 3.51 (t, J=8.9 Hz, 1H, pyrrolidine), 2.16 (s, 1H, NCH_3). HRMS (ESI, m/z): calculated 490.1099 ($C_{25}H_{20}ClN_5O_2S$, $[M+H]^+$), found 490.1105.

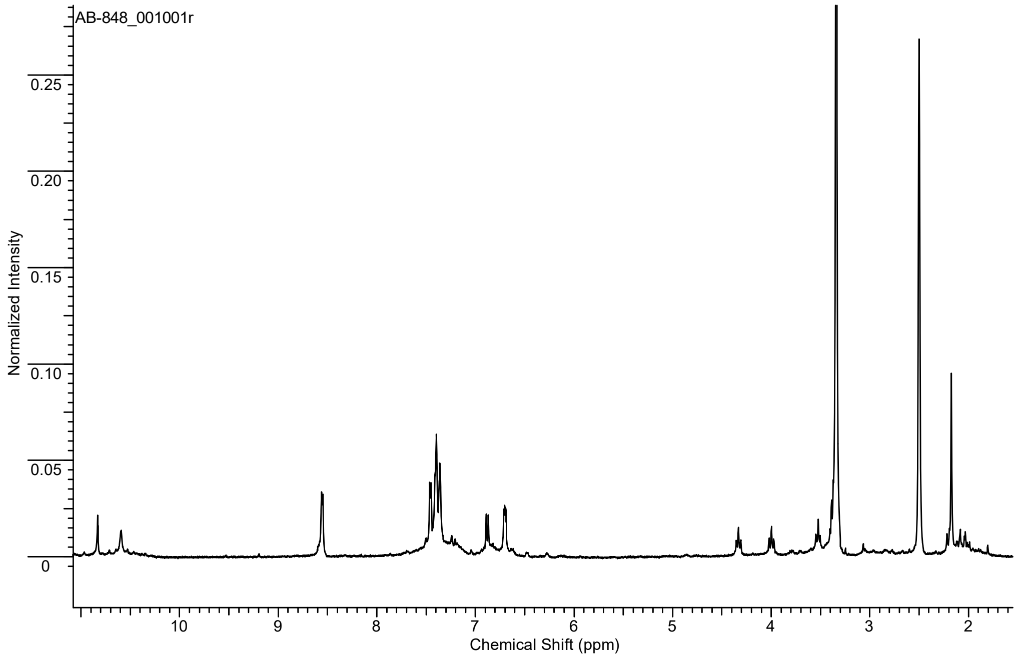

Figure S8. ^{1}H NMR spectra of compound **21**.

1-phenyl-4'-(3,4-dichlorophenyl)-1'-methyl-5'''-chloro-2-thiooxodispiro[imidazolidine-4,3'-pyrrolidine-2',3''-indoline] -2'',5-dione (22). Yield 68%, white solid. M.p. 215-216 ºC. ^{1}H NMR (400 MHz, DMSO-d6, δ, ppm): 10.82 (s, 1H, NH-indolinone), 10.64 (s, 1H, NH-imidazolidine), 7.78 (s, 1H, aryl), 7.59 (d, J=8.0 Hz, 1H, aryl), 7.40-7.37 (m, 5H, aryl), 7.32 (s, 1H, aryl), 6.88 (d, J=8.2, 1H, aryl), 6.70-6.63 (m, 2H, aryl), 4.34 (t, J=9.2 Hz, 1H, pyrrolidine), 3.92 (t, J=9.0 Hz, 1H, pyrrolidine), 3.51 (t, J=8.6 Hz, 1H, pyrrolidine), 2.16 (s, 3H, NCH_3). HRMS (ESI, m/z): calculated 557.0367 ($C_{26}H_{19}Cl_3N_4O_2S$, $[M+H]^+$), found 557.0370.

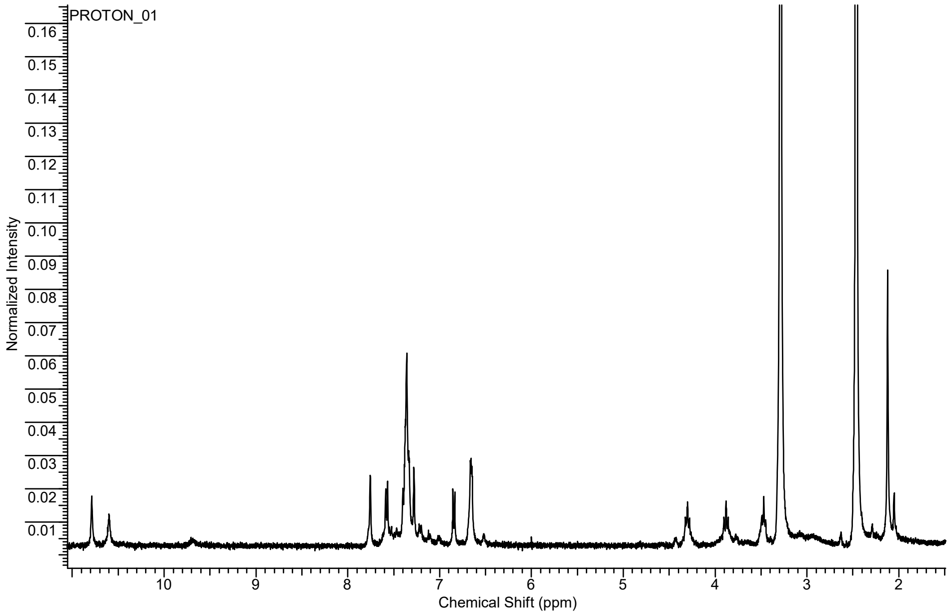

Figure S9. ^{1}H NMR spectra of compound **22**.

1-phenyl-4'-(3-chlorophenyl)-1'-methyl-5'''-chloro-2-thiooxodispiro[imidazolidine-4,3'-pyrrolidine-2',3''-indoline]-2'',5-dione (23). Yield 68%, white solid. M.p. 247-248 °C. ^{1}H NMR (400 MHz, DMSO-d6, δ, ppm): 10.80 (s, 1H, NH-indolinone), 10.60 (s, 1H, NH-imidazolidine), 7.56 (s, 1H, aryl), 7.44-7.34 (m, 8H, aryl), 6.88 (d, J=8.6 Hz, 1H, aryl), 6.70-6.64 (m, 2H, aryl), 4.35 (t, J=9.4 Hz, 1H, pyrrolidine), 3.94 (t, J=9.4 Hz, 1H, pyrrolidine), 3.50 (t, J=9.4 Hz, 1H, pyrrolidine), 2.17 (s, 3H, NCH_3). HRMS (ESI, m/z): calculated 522.0744 ($C_{26}H_{20}Cl_2N_4O_2S$, $[M+H]^+$), found 522.0748.

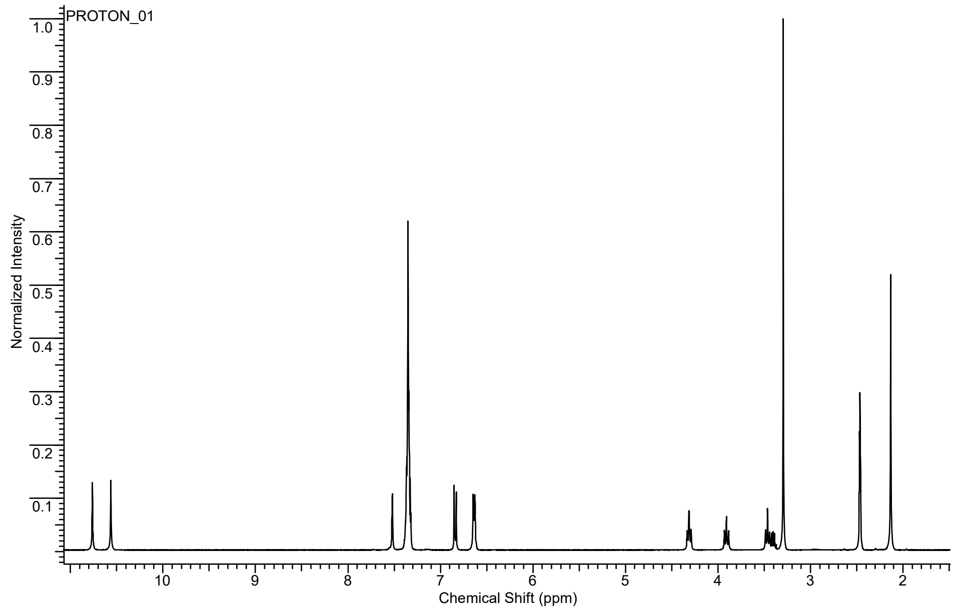

Figure S20. ^{1}H NMR spectra of compound **23**.

1-phenyl-4'-(3-formylphenyl)-1'-methyl-5'''-chloro-2-thiooxodispiro[imi-dazolidine-4,3'-pyrrolidine-2',3''-indoline]-2'',5-dione (24). Yield 71%, white solid. M.p. 245-246 °C. ^{1}H NMR (400 MHz, DMSO-d6, δ, ppm): 10.82 (s, 1H, CHO), 10.54 (s, 1H, NH-indolinone), 10.02 (s, 1H, NH-imidazolidine), 8.02 (s, 1H, aryl), 7.83 (d, J=7.6 Hz, 2H, aryl), 7.59 (t, J=7.5 Hz, 1H, aryl), 7.40-7.34 (m, 6H, aryl), 6.67-6.62 (m, 2H, aryl), 4.44 (t, J=9.3 Hz, 1H, pyrrolidine), 4.02 (t, J=9.3 Hz, 1H, pyrrolidine), 3.54 (t, J =9.3 Hz, 1H, pyrrolidine), 2.18 (s, 3H, NCH_3). HRMS (ESI, m/z): calculated 517.1096 ($C_{27}H_{21}ClN_4O_3S$, $[M+H]^+$), found 517.1102.

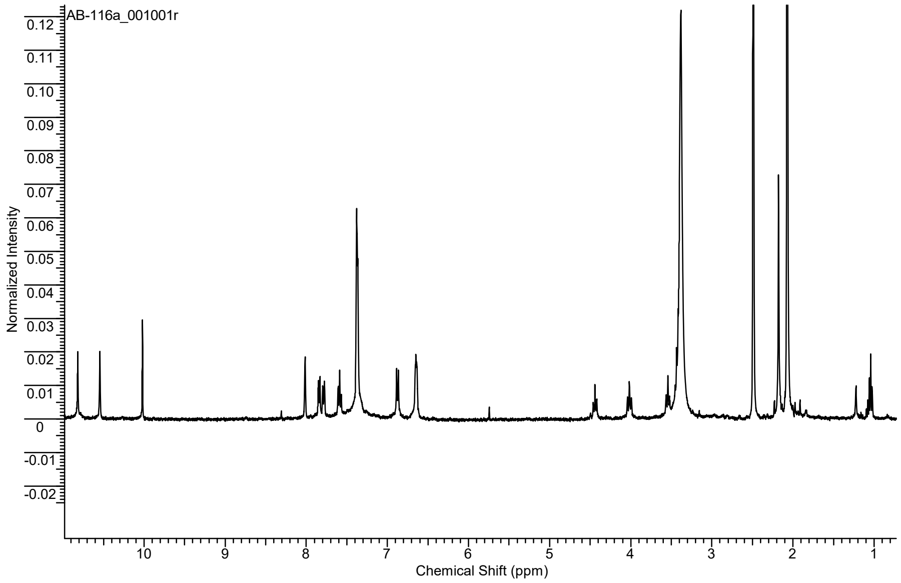

Figure S21. ^{1}H NMR spectra of compound **24**.

1-phenyl-4'-cyclohexyl-1'-methyl-2-thiooxodispiro[imidazolidine-4,3'-pyrrolidine-2',3"-indoline]-2",5-dione (25). Yield 36%, white solid. M.p. 236-237 °C. ^{1}H NMR (400 MHz, DMSO-d6, δ, ppm): 10.69 (s, 1H, NH-indolinone), 10.57 (s, 1H, NH-imidazolidine), 7.41-7.34 (m, 3H, aryl), 7.27 (td, J_1=1.1 Hz, J_2=7.7 Hz, 1H, aryl), 7.13 (d, J=7.4 Hz, 1H, aryl), 6.95 (td, J_1=0.7 Hz, J_2=7.6 Hz, 1H, aryl), 6.81 (dd, J=7.6 Hz, 1H, aryl), 6.64-6.57 (m, 2H, aryl), 3.51 (t, J=9.3 Hz, 1H, pyrrolidine), 3.14 (t, J=8.1 Hz, 1H, pyrrolidine), 2.71 (q, J=10.1 Hz, 1H, pyrrolidine), 2.05 (s, 3H, NCH_3), 1.89 (m, 1H, cyclohexyl), 1.74-1.63 (m, 2H, cyclohexyl), 1.63-1.54 (m, 2H, cyclohexyl), 1.38 (m, 1H, cyclohexyl), 1.31-1.11 (m, 3H, cyclohexyl), 1.05 (m, 1H, cyclohexyl), 0.77 (m, 1H, cyclohexyl). HRMS (ESI, m/z): calculated 483.1825 ($C_{26}H_{28}N_4O_2S$ $[M+Na]^+$), found 483.1804.

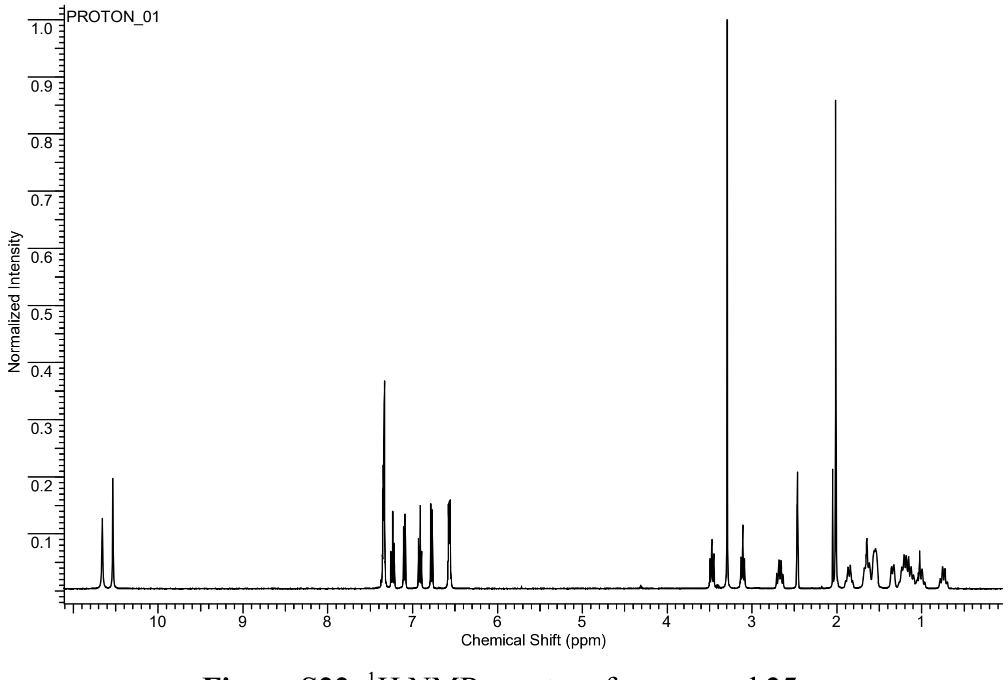

Figure S22. ^{1}H NMR spectra of compound **25**.

1-phenyl-4’-cyclohexyl-1’-methyl-5'''-bromo-2-thiooxodispiro[imidazolidine-4,3’-pyrrolidine-2’,3”-indoline]-2”,5-dione (26). Yield 35%, white solid. M.p. 228-229 °C. ^{1}H NMR (400 MHz, DMSO-d6, δ, ppm): 10.85 (s, 1H, NH-indolinone), 10.75 (s, 1H, NH-imidazolidine), 7.48 (d, J=7.2 Hz, 1H, aryl), 7.45-7.36 (m, 3H, aryl), 7.24 (s, 1H, aryl), 6.79 (d, J=8.2 Hz, 1H, aryl), 6.69-6.63 (m, 2H, aryl), 3.48 (t, J=9.1 Hz, 1H, pyrrolidine), 3.17 (t, J=8.2 Hz, 1H, pyrrolidine), 2.69 (q, J=9.4 Hz,1H, pyrrolidine), 2.07 (s, 3H, NCH_3), 1.87 (m, 1H, cyclohexyl), 1.74-1.62 (m, 2H, cyclohexyl), 1.62-1.53 (m, 2H, cyclohexyl), 1.37 (m, 1H, cyclohexyl), 1.30-1.11 (m, 3H, cyclohexyl), 1.05 (m, 1H, cyclohexyl), 0.78 (m, 1H, cyclohexyl). HRMS (ESI, m/z): calculated 541.1091 ($C_{26}H_{27}BrN_4O_2S$ $[M+H]^+$), found 541.1097.

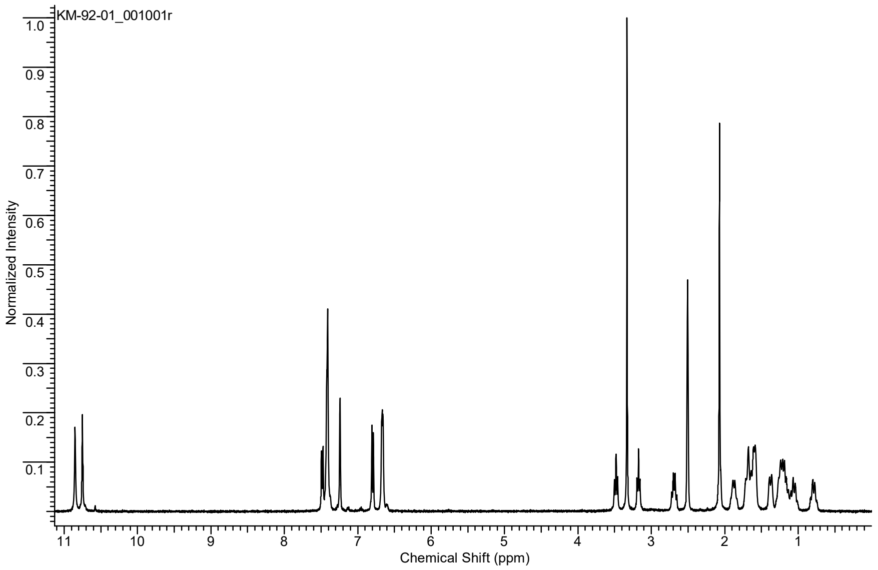

Figure S23. ^{1}H NMR spectra of compound **26**.

1-(4-ethoxyphenyl)-4'-(3,4-dichlorophenyl)-1'-methyl-2-thiooxodispiro[imidazolidine-4,3’-pyrrolidine-2',3”-indoline]-2”,5-dione (27). Yield 79%, white solid. M.p. 273-274 °C. ^{1}H NMR (400 MHz, DMSO-d6, δ, ppm): 10.70 (s, 1H, NH-indolinone), 10.31 (s, 1H, NH-imidazolidine), 7.76 (bs, 1H, aryl), 7.69 (d, J=8.4 Hz, 2H, aryl), 7.39 (d, J=7.8 Hz, 1H, aryl), 7.34-7.22 (m, 2H, aryl), 6.99 (t, J=7.6 Hz, 1H, aryl), 6.92-6.81 (m, 3H, aryl), 6.53 (d, J=8.4 Hz, 1H, aryl), 4.28 (t, J=8.8 Hz, 1H, pyrrolidine), 3.99 (q, J=6.9 Hz, 2H, CH_2CH_3), 3.94 (t, J=8.8 Hz, 1H, pyrrolidine), 3.46 (t, J=8.8 Hz, 1H, pyrrolidine), 2.11 (s, 3H, NCH_3), 1.29 (t, J=6.7 Hz, 3H, CH_2CH_3). HRMS (ESI, m/z): calculated 567.1019 ($C_{28}H_{24}Cl_2N_4O_3S$, $[M+H]^+$), found 567.1021.

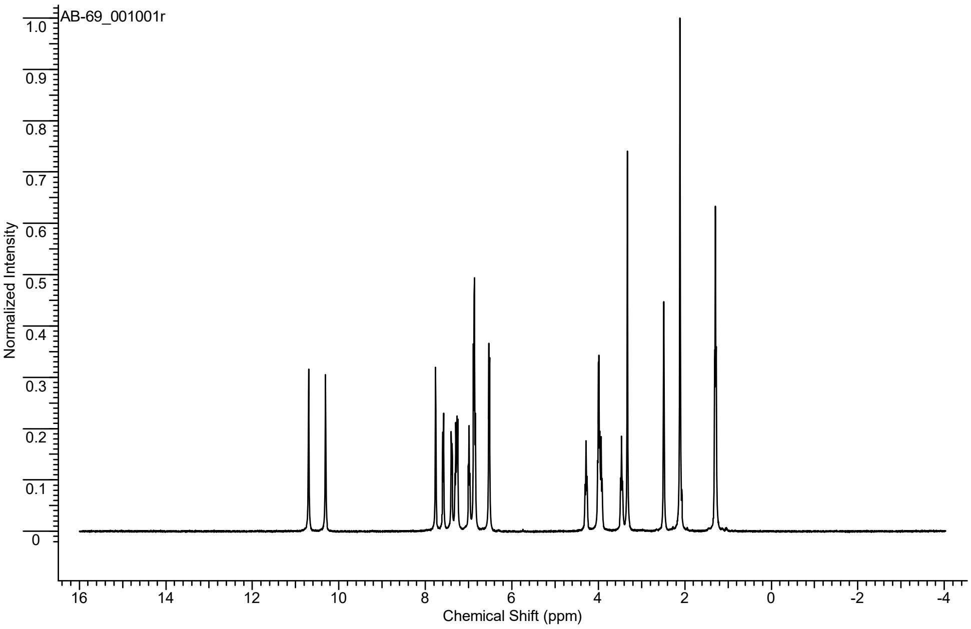

Figure S24. ^{1}H NMR spectra of compound **27**.

1-(4-ethoxyphenyl)-4'-(4-chlorophenyl)-1'-methyl-2-thiooxodispiro[imidazolidine-4,3’-pyrrolidine-2',3”-indoline]-2”,5-dione (28) [2]. Yield 62%, white solid. M.p. 213-215 °C. ^{1}H NMR (400 MHz, DMSO-d6, δ, ppm): 10.68 (s, 1H, NH-indolinone), 10.12 (s, 1H, NH-imidazolidine), 7.46 (d, J=8.6 Hz, 4H, aryl), 7.33-7.29 (m, 2H, aryl), 7.01 (t, J=7.6 Hz, 1H, aryl), 6.90-6.84 (m, 3H, aryl), 6.52 (t, J=8.8 Hz, 2H, aryl), 4.31 (t, J=8.6 Hz, 1H, pyrrolidine), 3.99 (q, J=6.7 Hz, 2H, CH_2CH_3), 3.46 (t, J=8.6 Hz, 1H, pyrrolidine) , 3.05 (t, J=8.6 Hz, 1H, pyrrolidine), 2.14 (s, 3H, NCH_3), 1.31 (t, J=6.7 Hz, 3H, CH_2CH_3).

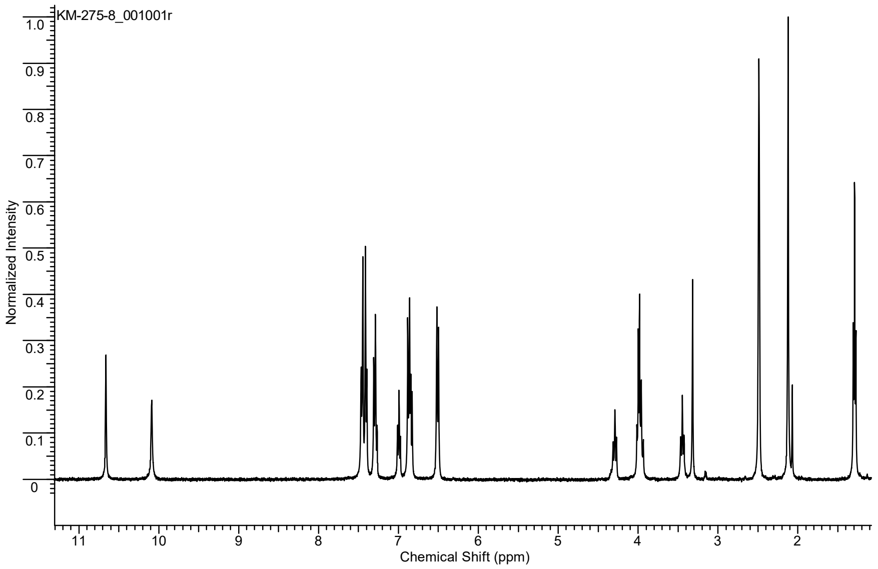

Figure S25. ^{1}H NMR spectra of compound **28**.

1-(4-ethoxyphenyl)-4'-(4-chlorophenyl)-1'-methyl-5'''-bromo-2-thiooxodispiro[imidazolidine-4,3’-pyrrolidine-2',3”-indoline]-2”,5-dione (29) [2]. Yield 89%, white

solid. M.p. 301-302 °C. ^{1}H NMR (400 MHz, DMSO-d6, δ, ppm): 10.82 (s, 1H, NH-indolinone), 10.38 (s, 1H, NH-imidazolidine), 7.54-7.38 (m, 6H, aryl), 6.90 (d, J=8.6 Hz, 2H, aryl), 6.81 (d, J=8.3 Hz, 1H, aryl), 6.56 (t, J=8.6 Hz, 2H, aryl), 4.31 (t, J=9.0 Hz, 1H, pyrrolidine), 4.02 (q, J=6.9 Hz, 2H, CH_2CH_3), 3.49 (t, J=9.0 Hz, 1H, pyrrolidine), 3.02 (t, J=9.0 Hz, 1H, pyrrolidine), 2.16 (s, 3H, NCH_3), 1.31 (t, J=6.9 Hz, 3H, CH_2CH_3).

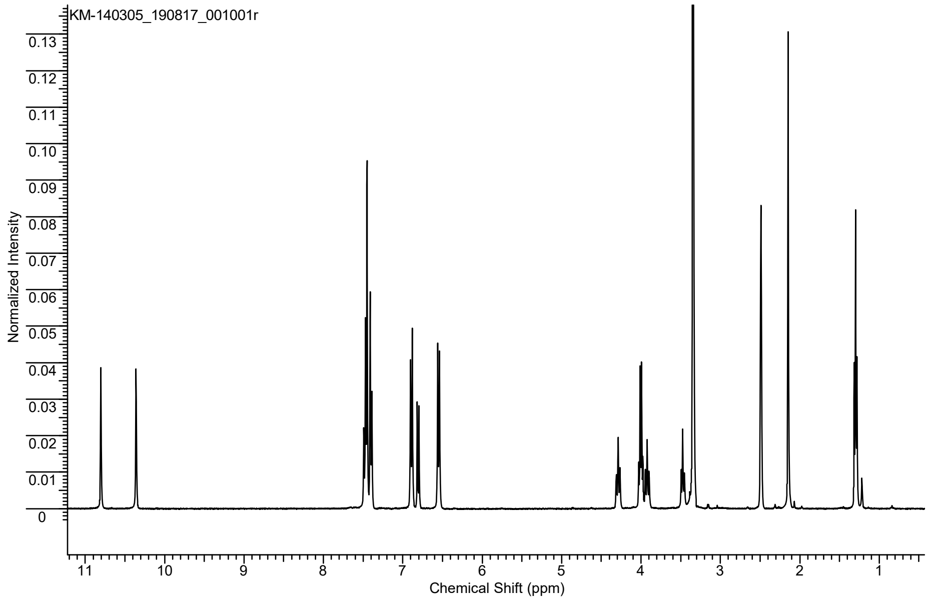

Figure S26. ^{1}H NMR spectra of compound **29**.

1-(4-ethoxyphenyl)-4'-(4-fluorophenyl)-1'-methyl-2-thiooxodispiro[imidazolidine-4,3'-pyrrolidine-2',3"-indoline]-2",5-dione (30). Yield 72%, white solid. M.p. 232-233 °C. ^{1}H NMR (400 MHz, DMSO-d6, δ, ppm): 10.66 (s, 1H, NH-indolinone), 10.06 (s, 1H, NH-imidazolidine), 7.49 (dd, J_1=6.6 Hz, J_2=8.6 Hz, 2H, aryl), 7.33 (d, J=7.6 Hz, 1H, aryl), 7.29 (d, J=7.6 Hz, 1H, aryl), 7.19(t, J=8.6 Hz, 2H, aryl), 7.01 (t, J=7.6 Hz, 1H, aryl), 6.88 (d, J=8.6 Hz, 2H, aryl), 6.85 (d, J=7.9Hz, 1H, aryl), 6.51 (d, J=8.9 Hz, 2H, aryl), 4.32 (t, J=9.2 Hz, 1H, pyrrolidine), 4.04-3.92 (m, 3H, pyrrolidine + CH_2CH_3), 3.46 (t, J=8.9 Hz, 1H, pyrrolidine), 2.14 (s, 3H, NCH_3), 1.30 (t, J=7.0 Hz, 3H, CH_2CH_3). HRMS (ESI, m/z): calculated 517.1704 ($C_{28}H_{25}FN_4O_3S$, $[M+H]^+$), found 517.1707.

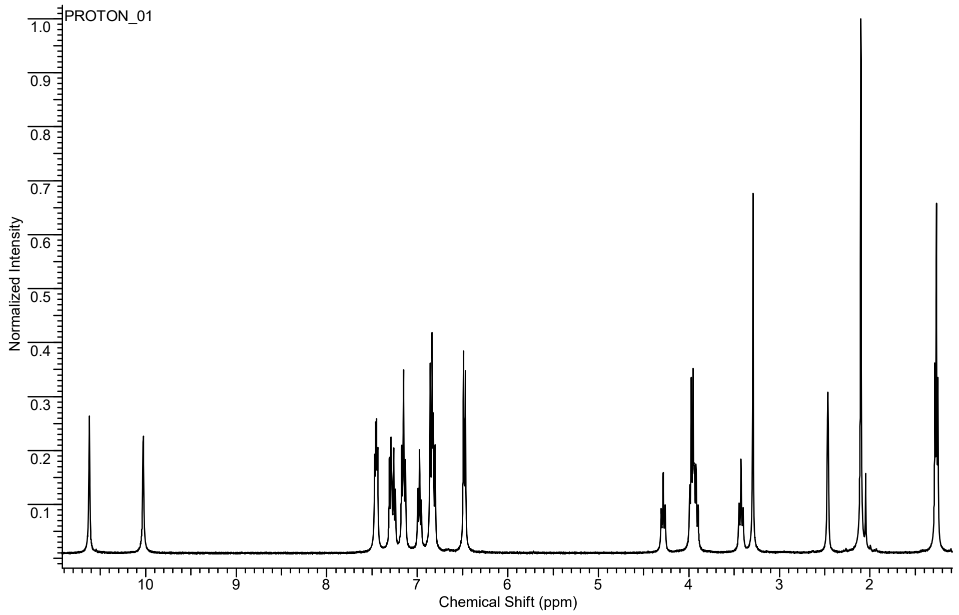

Figure S27. ^{1}H NMR spectra of compound **30**.

1-(4-ethoxyphenyl)-4'-(4-fluorophenyl)-1'-methyl-5'''-bromo-2-thiooxodispiro[imidazolidine-4,3'-pyrrolidine-2',3''-indoline]-2'',5-dione (31). Yield 68%, white solid. M.p. 220-221 °C. ^{1}H NMR (400 MHz, DMSO-d6, δ, ppm): 10.79 (s, 1H, NH-indolinone), 10.33 (s, 1H, NH-imidazolidine), 7.53-7.46 (m, 4H, aryl), 7.15 (t, J=8.8 Hz, 2H, aryl), 6.90 (d, J=8.7 Hz, 2H, aryl), 6.82 (d, J=8.9 Hz, 1H, aryl), 6.55 (d, J=8.9 Hz, 2H, aryl), 4.31 (t, J=9.2 Hz, 1H, pyrrolidine), 4.01 (q, J=7.0 Hz, 2H, CH_2CH_3), 3.92 (t, J=9.2 Hz, 1H, pyrrolidine), 3.49 (t, J=9.2 Hz, 1H, pyrrolidine), 2.16 (s, 3H, NCH_3), 1.27 (t, J=7.0 Hz, 3H, CH_2CH_3). HRMS (ESI, m/z): calculated 597.0790 ($C_{28}H_{24}BrFN_4O_3S$, $[M+H]^+$), found 597.0778.

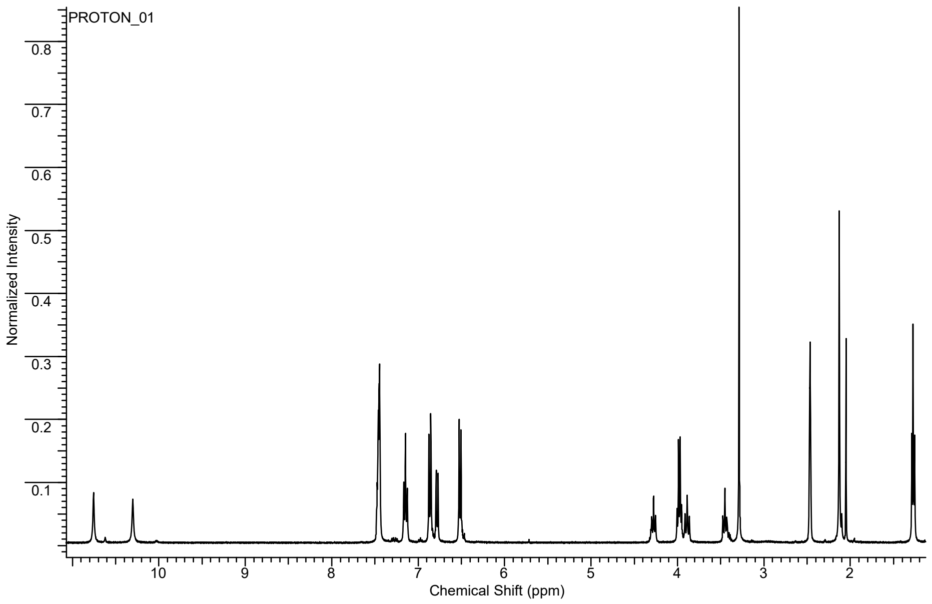

Figure S28. ^{1}H NMR spectra of compound **31**.

1-(4-methoxyphenyl)-4’-(3-chlorophenyl)-1’-methyl-2-thiooxodispiro[imidazolidine-4,3’-pyrrolidine-2’,3”-indoline]-2”,5-dione (32). Yield 59%, white solid. M.p. 230-231 °C. ^{1}H NMR (400 MHz, DMSO-d6, δ, ppm): 10.65 (s, 1H, NH-indolinone), 10.18 (s, 1H, NH-imidazolidine), 7.54 (s, 1H, aryl), 7.38 (s, 3H, aryl), 7.30 (m, 2H, aryl), 7.01 (t, J=7.5 Hz, 1H, aryl), 6.91 (d, J=8.8 Hz, 2H, aryl), 6.86 (d, J=7.8 Hz, 1H, aryl), 6.53 (d, J=8.8 Hz, 2H, aryl), 4.31 (t, J=9.1 Hz, 1H, aryl), 3.98 (t, J=9.3 Hz, 1H, pyrrolidine), 3.74 (s, 3H, OCH_3), 3.47 (t, J=8.5 Hz, 1H, pyrrolidine), 2.14 (s, 3H, NCH_3). HRMS (ESI, m/z): calculated 541.1072 ($C_{27}H_{23}ClN_4O_3S$ $[M+Na]^+$), found 541.1067.

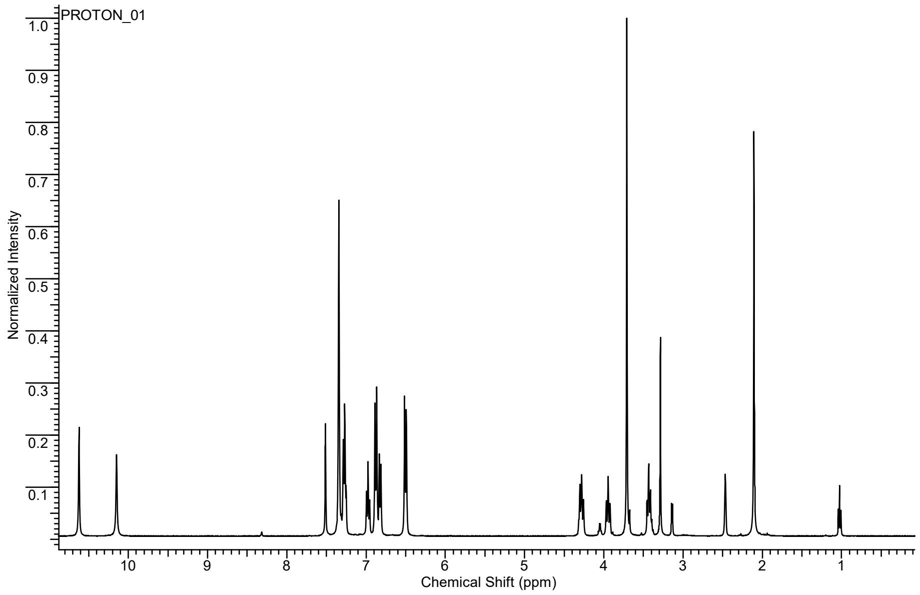

Figure S29. ^{1}H NMR spectra of compound **33**.

1-(4-methoxyphenyl)-4’-(3-chlorophenyl)-1’-methyl-5'''-bromo-2-thiooxodispiro[imidazolidine-4,3’-pyrrolidine-2’,3”-indoline]-2”,5-dione (33). Yield 70%, white solid. M.p. 229-230 °C. ^{1}H NMR (400 MHz, DMSO-d6, δ, ppm): 10.79 (s, 1H, NH-indolinone), 10.45 (s, 1H, NH-imidazolidine), 7.55 (s, 1H, aryl), 7.50 (dd, J_1=1.7 Hz, J_2=8.3 Hz, 1H, aryl), 7.46 (m, 1H, aryl), 7.38 (s, J=7.5 Hz, 1H, aryl), 6.93 (d, J=8.8 Hz, 2H, aryl), 6.82 (d, J=7.8 Hz, 1H, aryl), 6.58 (d, J=8.8 Hz, 2H, aryl), 4.31 (t, J=9.2 Hz, 1H, aryl), 3.98 (t, J=9.4 Hz, 1H, pyrrolidine), 3.75 (s, 3H, OCH_3), 3.47 (t, J=8.6 Hz, 1H, pyrrolidine), 2.16 (s, 3H, NCH_3). HRMS (ESI, m/z): calculated 599.0337 ($C_{27}H_{22}BrClN_4O_3S$ $[M+H]^+$), found 599.0353.

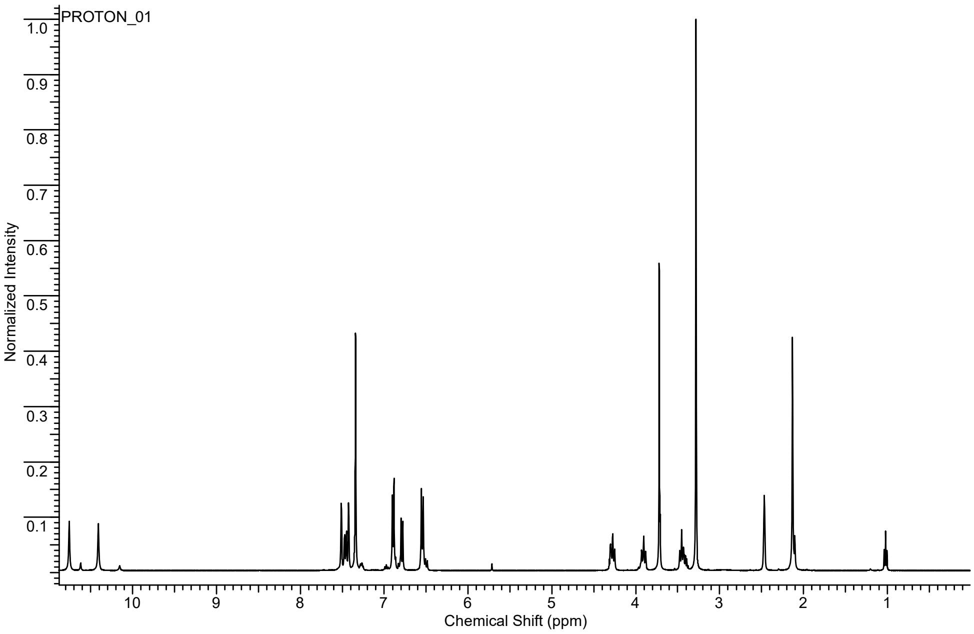

Figure S30. ^{1}H NMR spectra of compound **33**.

1-(4-ethoxyphenyl)-4'-(4-(prop-2-yn-1-yl)phenyl)-1'-methyl-5'''-bromo-2-thiooxodispiro[imidazolidine-4,3'-pyrrolidine-2',3''-indoline]-2'',5-dione (34). Yield 59%, white solid. M.p. 218-220 °C. ^{1}H NMR (400 MHz, DMSO-d6, δ, ppm): 10.78 (s, 1H, NH-indolinone), 10.28 (s, 1H, NH-imidazolidine), 7.51-7.45 (m, 2H, aryl), 7.38 (d, J=8.7 Hz, 2H, aryl), 6.96 (d, J=8.7 Hz, 2H, aryl), 6.89 (d, J=8.9 Hz, 2H, aryl), 6.81 (d, J=8.8 Hz, 1H, aryl), 6.54 (d, J=8.9 Hz, 2H), 4.79 (d, J=2.2 Hz, 2H, CH_2), 4.25 (t, J=9.2 Hz, 1H, pyrrolidine), 4.01 (q, J=7.0 Hz, 2H, CH_2CH_3), 3.90 (t, J=9.3 Hz, 1H, pyrrolidine), 3.57 (t, J=2.2 Hz, 1H, CCH), 3.45 (t, J=8.7 Hz, 1H, pyrrolidine), 2.16 (s, 3H, NCH_3), 1.31 (t, J=7.1 Hz, 3H, CH_2CH_3). HRMS (ESI, m/z): calculated 631.1009 ($C_{31}H_{27}BrClN_4O_4S$, $[M+H]^+$), found 631.1011.

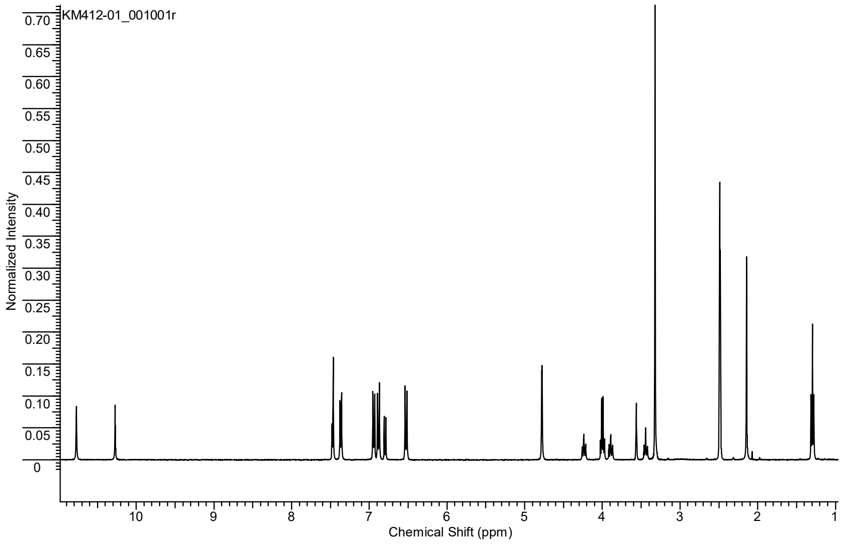

Figure S31. ^{1}H NMR spectra of compound **34**.

1-(4-(prop-2-yn-1-yl)phenyl)-4'-(4-chlorophenyl)-1'-methyl-5'''-bromo-2-thiooxodispiro[imidazolidine-4,3'-pyrrolidine-2',3''-indoline]-2'',5-dione (35). Yield 94%, white solid. M.p. 238-239 °C. ^{1}H NMR (400 MHz, DMSO-d6, δ, ppm): 10.85 (s, 1H, NH-indolinone), 10.41 (s, 1H, NH-imidazolidine), 7.51-7.38 (m, 6H, aryl), 6.97 (d, J=8.7 Hz, 2H, aryl), 6.82 (d, J=8.2 Hz, 2H, aryl), 6.59 (d, J=8.7 Hz, 2H, aryl), 4.80 (d, J=2.0 Hz, 2H, CH_2), 4.29 (t, J=9.0 Hz, 1H, pyrrolidine), 3.92 (t, J=10.0 Hz, 1H, pyrrolidine), 3.59 (t, J=2.0 Hz, 1H, CCH), 3.48 (t, J=8.8 Hz, 1H, pyrrolidine), 2.15 (s, 3H, NCH_3). HRMS (ESI, m/z): calculated 621.0357 ($C_{29}H_{22}BrClN_4O_3S$ $[M+H]^+$), found 621.0358.

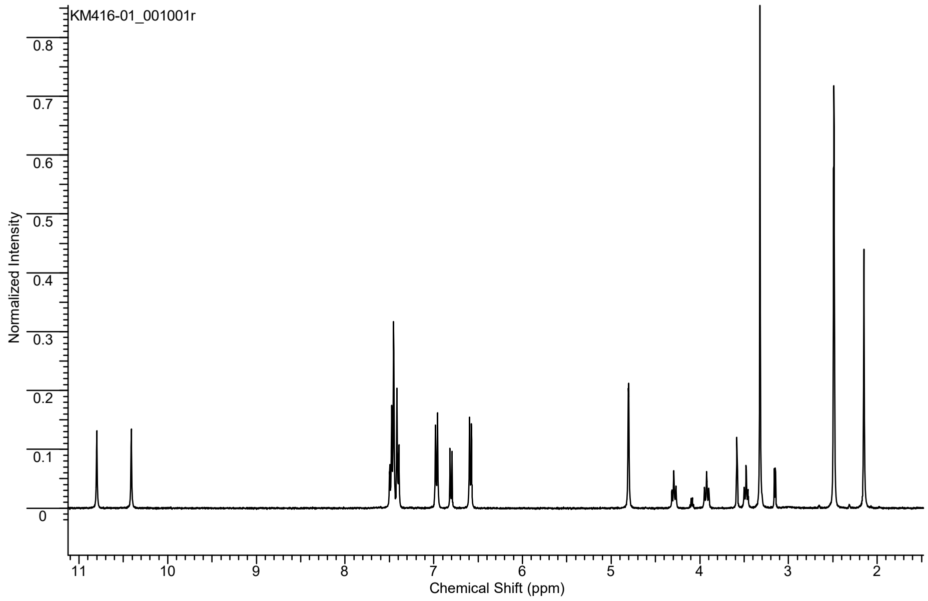

Figure S32. ^{1}H NMR spectra of compound **35**.

1-(4-chlorophenyl)-4'-(2-chlorophenyl)-1'-methyl-5'''-chloro-2-thiooxodispiro[imidazolidine-4,3'-pyrrolidine-2',3''-indoline]-2'',5-dione (36). Yield 83%, white solid. M.p. 203-204 °C. ^{1}H NMR (400 MHz, DMSO-d6, δ, ppm): 10.86 (s, 1H, NH-indolinone), 10.17 (s, 1H, NH-imidazolidine), 7.92 (d, J=7.7 Hz, 1H, aryl), 7.60-7.29 (m, 6H, aryl), 7.10 (s, 1H, aryl), 6.87 (d, J=8.3 Hz, 1H, aryl), 6.67 (d, J=8.4 Hz, 2H, aryl), 4.56 (t, J=8.8 Hz, 1H, pyrrolidine), 4.25 (t, J=9.2 Hz, 1H, pyrrolidine), 3.47 (t, J=8.6 Hz, 1H, pyrrolidine), 2.16 (s, 3H, NCH_3). HRMS (ESI, m/z): calculated 557.0367 ($C_{26}H_{19}Cl_3N_4O_2S$ $[M+H]^+$), found 557.0384.

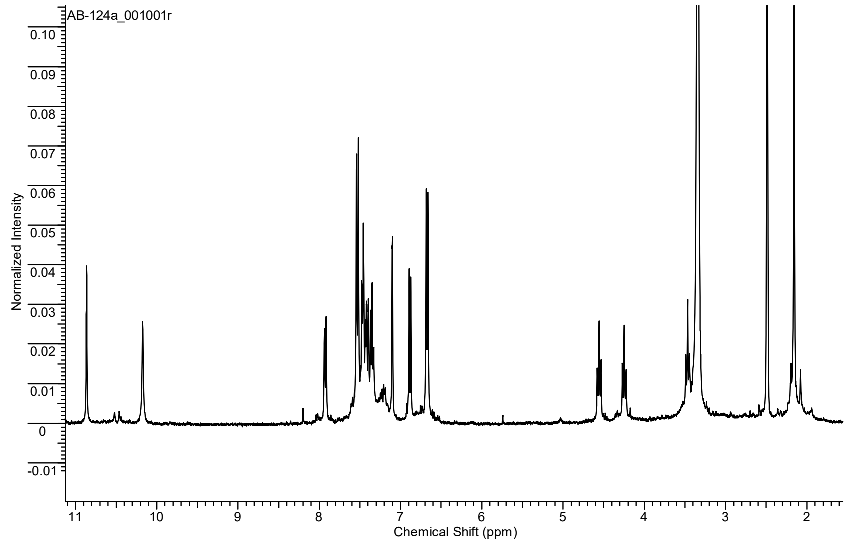

Figure S33. ^{1}H NMR spectra of compound **36**.

1-(4-chlorophenyl)-4'-(4-chlorophenyl)-1'-methyl-5'''-chloro-2-thiooxodispiro[imidazolidine-4,3'-pyrrolidine-2',3''-indoline]-2'',5-dione (37). Yield 85%, white solid. M.p. 211-212 ºC. ^{1}H NMR (400 MHz, DMSO-d6, δ, ppm): 10.82 (s, 1H, NH-indolinone), 10.64 (s, 1H, NH-imidazolidine), 7.54-7.33 (m, 8H, aryl), 6.87 (d, J=8.35 Hz, 1H, aryl), 6.72 (d, J=8.4 Hz, 2H, aryl), 4.34 (t, J=8.8 Hz, 1H, pyrrolidine), 3.93 (t, J=9.4 Hz, 1H, pyrrolidine), 3.50 (t, J=8.8 Hz, 1H, pyrrolidine), 2.16 (s, 3H, NCH_3). HRMS (ESI, m/z): calculated 557.0367 ($C_{26}H_{19}Cl_3N_4O_2S$ $[M+H]^+$), found 557.0383.

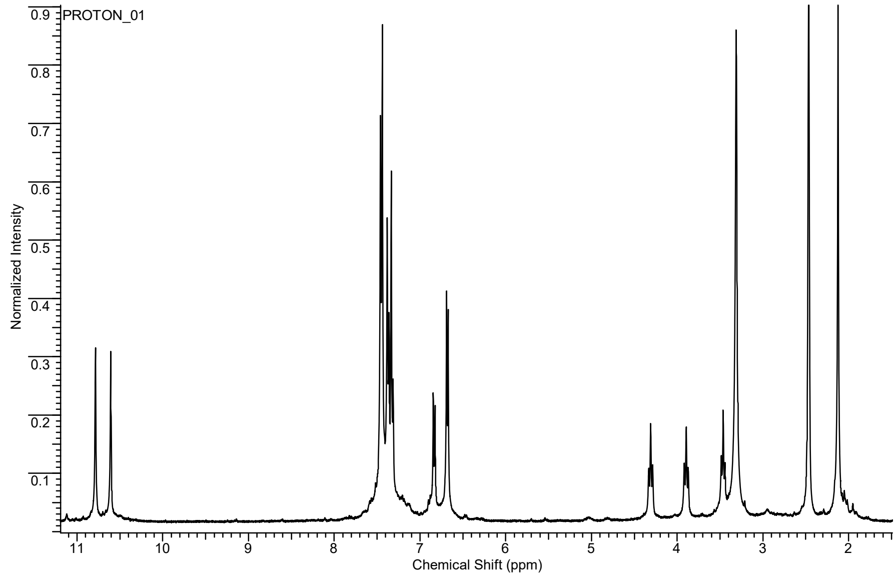

Figure S34. ^{1}H NMR spectra of compound **37**.

1-(4-chlorophenyl)-4'-(4-fluorophenyl)-1'-methyl-2-thiooxodispiro[imidazolidine-4,3'-pyrrolidine-2',3''-indoline]-2'',5-dione (38). Yield 85%, white solid. M.p. 225-226 ºC. ^{1}H NMR (400 MHz, DMSO-d6, δ, ppm): 10.66 (s, 1H, NH-indolinone), 10.37 (s, 1H, NH-imidazolidine), 7.46-7.42

(m, 4H, aryl), 7.33-7.28 (m, 2H, aryl), 7.18 (t, J=7.7 Hz, 2H, aryl), 7.01 (t, J=7.7 Hz, 1H, aryl), 6.85 (d, J=6.9 Hz, 1H, aryl), 6.67 (t, J=7.2 Hz, 2H, aryl), 4.32 (t, J=8.5 Hz, 1H, pyrrolidine), 3.94 (t, J=9.3 Hz, 1H, pyrrolidine), 3.48 (t, J=10.0 Hz, 1H, pyrrolidine), 2.12 (s, 3H, NCH_3). HRMS (ESI, m/z): calculated 507.1048 ($C_{26}H_{20}ClFN_4O_2S$ $[M+H]^+$), found 507.1044.

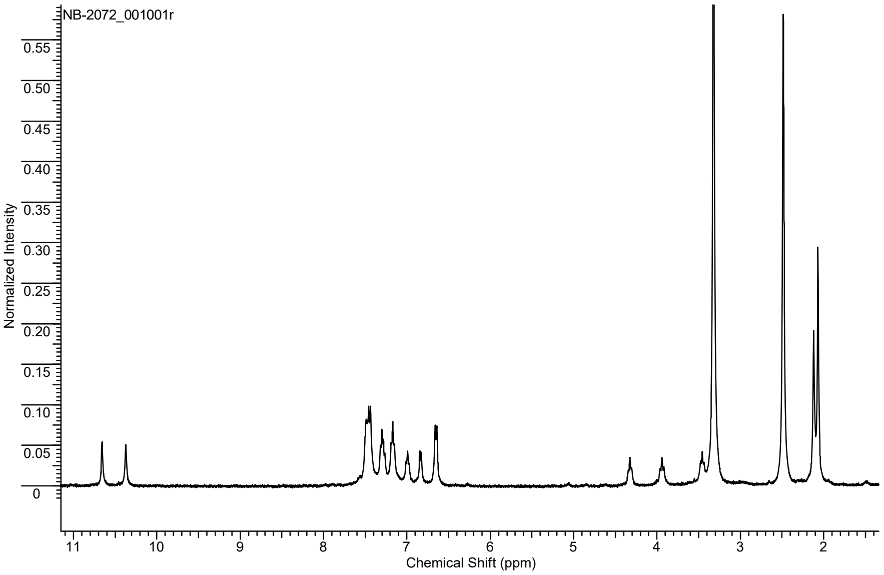

Figure S35. ^{1}H NMR spectra of compound **38**.

1-(4-chlorophenyl)-4’-(3,4-dimethoxyphenyl)-1’-methyl-5’’’-chloro-2-thiooxodispiro[imidazolidine-4,3’-pyrrolidine-2’,3”-indoline]-2”,5-dione (39). Yield 81%, white solid. M.p. 198-199 °C. ^{1}H NMR (400 MHz, DMSO-d6, δ, ppm): 10.75 (s, 1H, NH-indolinone), 10.67 (s, 1H, NH-imidazolidine), 7.47 (d, J=8.6 Hz, 2H, aryl), 7.40 (s, 1H, aryl), 7.37-7.31 (m, 1H, aryl), 7.26 (s, 1H, aryl), 6.89-6.76 (m, 3H, aryl), 6.67 (d, J=8.6 Hz, 2H, aryl), 4.25 (t, J=9.05 Hz, 1H, pyrrolidine), 3.89 (t, J=9.41 Hz, 1H, pyrrolidine), 3.80 (s, 3H, OCH_3), 3.72 (s, 3H, OCH_3), 3.47 (t, J=9.05 Hz, 1H, pyrrolidine), 2.15 (s, 3H, NCH_3). HRMS (ESI, m/z): calculated 583.0968 ($C_{28}H_{24}Cl_2N_4O_4S$ $[M+H]^+$): found 583.0976.

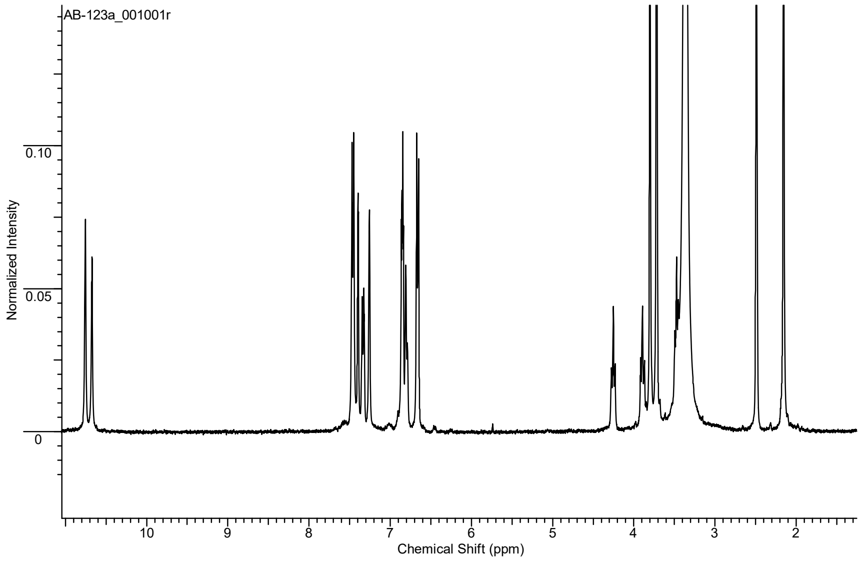

Figure S36. ^{1}H NMR spectra of compound **439**.

1-(4-fluorophenyl)-4'-(4-chlorophenyl)-1'-methyl-5'''-bromo-2-thiooxodispiro[imidazolidine-4,3'-pyrrolidine-2',3''-indoline]-2'',5-dione (40). Yield 68%, white solid. M.p. 206-207 °C. ^{1}H NMR (400 MHz, DMSO-d6, δ, ppm): 10.81 (s, 1H, NH-indolinone), 10.56 (s, 1H, NH-imidazolidine), 7.49-7.45 (m, 4H, aryl), 7.41-7.39 (m, 2H, aryl), 7.24 (t, J=8.2 Hz, 2H, aryl), 6.81 (d, J=8.2 Hz, 1H, aryl), 6.73 (bs, 2H, aryl), 4.31 (t, J=10.5 Hz, 1H, pyrrolidine), 3.93 (t, J=9.9 Hz, 1H, pyrrolidine), 3.49 (t, J=8.2 Hz, 1H, pyrrolidine), 2.15 (s, 3H, NCH_3). HRMS (ESI, m/z): calculated 585.0157 ($C_{26}H_{19}BrClFN_4O_2S$ $[M+H]^+$): found 585.0166.

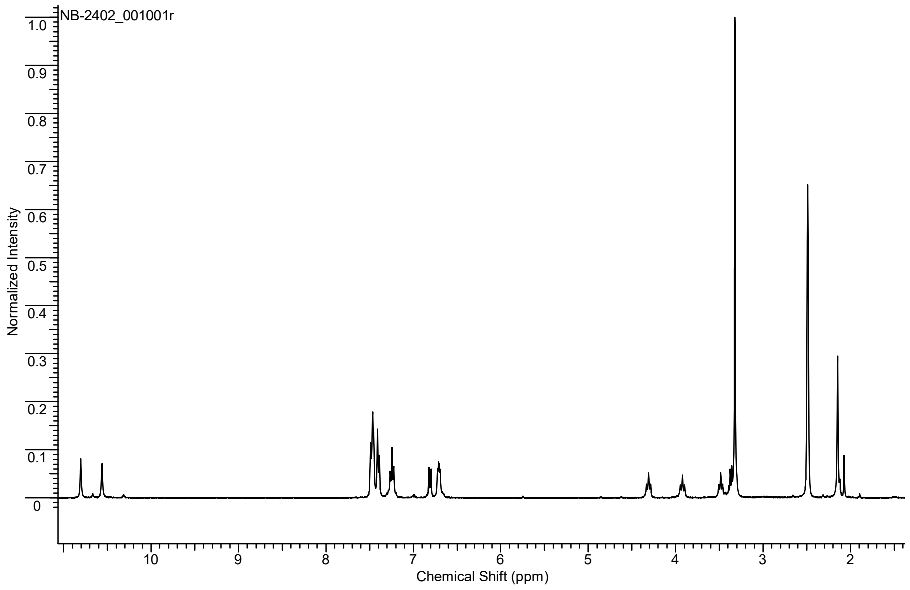

Figure S37. ^{1}H NMR spectra of compound **40**.

1-(3-chloro-4-fluorophenyl)-4'-(4-methoxyphenyl)-1'-methyl-2-thiooxodispiro[imidazolidine-4,3'-pyrrolidine-2',3''-indoline]-2'',5-dione (41). Yield 69%, white solid. M.p. 211-212 °C. ^{1}H NMR (400 MHz, DMSO-d6, δ, ppm): 10.65 (s, 1H, NH-indolinone), 10.33 (s, 1H, NH-imidazolidine), 7.46 (t,

J=9.0 Hz, 1H, aryl), 7.39 (d, J=8.7 Hz, 2H, aryl), 7.33-7.27 (m, 2H, aryl), 7.00 (t, J=7.6 Hz, 1H, aryl), 6.91 (d, J=8.7 Hz, 2H, aryl), 6.86 (d, J=8.2 Hz, 1H, aryl), 6.79 (dd, J=2.5, 6.6 Hz, 1H, aryl), 6.67 (m, 1H, aryl), 4.28 (t, J=9.2 Hz, 1H, pyrrolidine), 3.96 (t, J=9.4 Hz, 1H, pyrrolidine), 3.75 (s, 3H, OCH_3), 3.44 (t, J=8.9 Hz, 1H, pyrrolidine), 2.14 (s, 3H, NCH_3). HRMS (ESI, m/z): calculated 537.1158 ($C_{27}H_{22}ClFN_4O_3S$ $[M+H]^+$), found 537.1155.

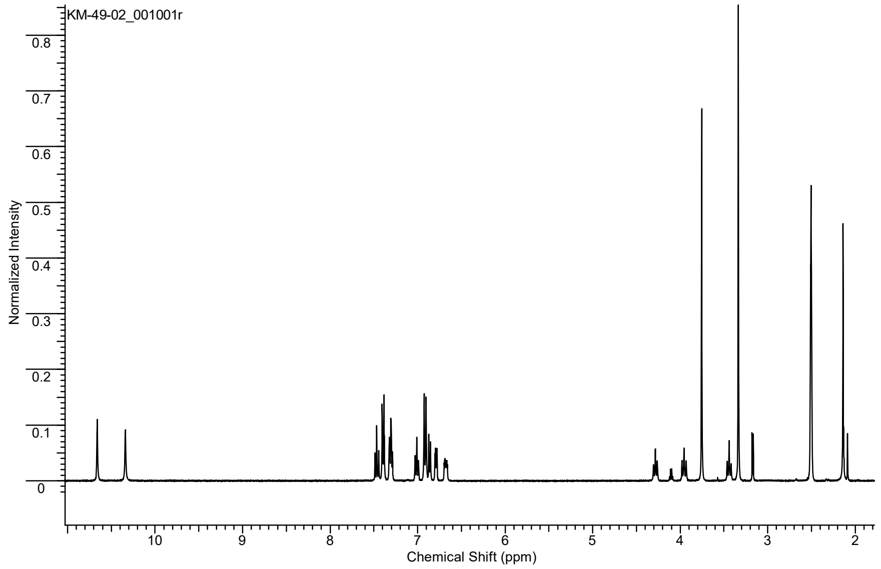

Figure S38. ^{1}H NMR spectra of compound **41**.

1-(3-chloro-4-fluorophenyl)-4'-(4-chlorophenyl)-1'-methyl-5'''-chloro-2-thiooxodispiro[imidazolidine-4,3'-pyrrolidine-2',3''-indoline]-2'',5-dione (42). Yield 73%, white solid. M.p. 215-216 °C. ^{1}H NMR (400 MHz, DMSO-d6, δ, ppm): 10.82 (s, 1H, NH-indolinone), 10.67 (s, 1H, NH-imidazolidine), 7.50 (t, J=8.6 Hz, 3H, aryl), 7.42-7.37 (m, 3H, aryl), 7.31 (s, 1H, aryl), 6.90-6.85 (m, 2H, aryl), 6.78-6.74 (m, 1H, aryl), 4.33 (t, J=9.7 Hz, 1H, pyrrolidine), 3.92 (t, J=9.2 Hz, 1H, pyrrolidine), 3.51 (t, J=9.7 Hz, 1H, pyrrolidine), 2.15 (s, 3H, NCH_3). HRMS (ESI, m/z): calculated 575.0273 ($C_{26}H_{18}Cl_3FN_4O_2S$ $[M+H]^+$), found 575.0278.

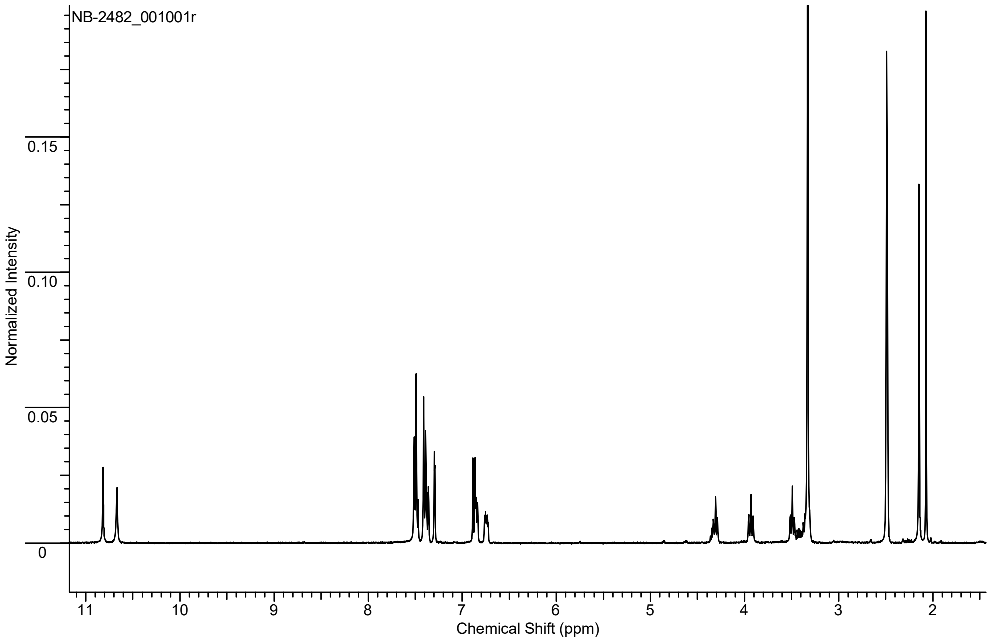

Figure S39. ^{1}H NMR spectra of compound **42**.

1-(3-chloro-4-fluorophenyl)-4’-(3-chlorophenyl)-1’-methyl-5’’’-chloro-2-thiooxodispiro[imidazolidine-4,3’-pyrrolidine-2’,3”-indoline]-2”,5-dione (43) [3]. Yield 79%, white solid. M.p. 224-225 ºC. ^{1}H NMR (400 MHz, DMSO-d6, δ, ppm): 10.81 (s, 1H, NH-indolinone), 10.48 (s, 1H, NH-imidazolidine), 7.56 (s, 1H, aryl), 7.36-7.40 (m, 6H, aryl), 6.82 (d, J=10.6 Hz, 1H, aryl), 6.67 (bs, 2H, aryl), 4.31 (t, J=9.2 Hz, 1H, pyrrolidine), 3.93 (t, J=9.9 Hz, 1H, pyrrolidine), 3.48 (t, J=8.5 Hz, 1H, pyrrolidine), 2.15 (s, 3H, NCH_3). 575.0273 HRMS (ESI, m/z): calculated 575.0273 ($C_{26}H_{18}Cl_3FN_4O_2S$ $[M+H]^+$), found 575.0276.

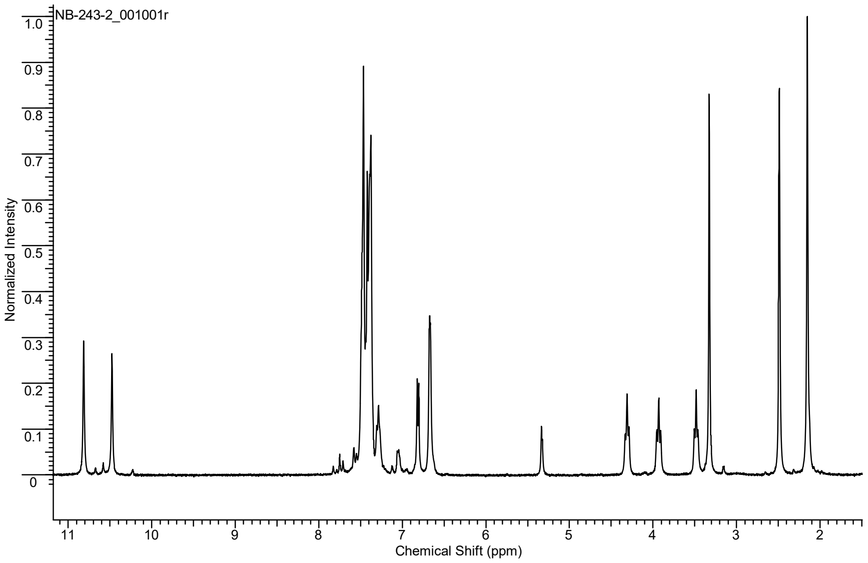

Figure S40. ^{1}H NMR spectra of compound **43**.

1-(3-chloro-4-fluorophenyl)-4’-(3,4-dimethoxyphenyl)-1’-methyl- -2-thiooxodispiro[imidazolidine-4,3’-pyrrolidine-2’,3”-indoline]-2”,5-dione (44). Yield 68%, white

solid. M.p. 196-197 ºC. ^{1}H NMR (400 MHz, DMSO-d6, δ, ppm): 10.63 (s, 1H, NH-indolinone), 10.48 (s, 1H, NH-imidazolidine), 7.46 (t, J=9.0 Hz, 1H, aryl), 7.35-7.25 (m, 3H, aryl), 7.00 (t, J=7.6 Hz, 1H, aryl), 6.90-6.81 (m, 3H, aryl), 6.75 (dd, J=2.5, 6.6 Hz, 1H, aryl), 6.68 (m, 1H, aryl), 4.25 (t, J=9.1 Hz, 1H, pyrrolidine), 3.95 (t, J=9.3 Hz, 1H, pyrrolidine), 3.83 (s, 3H, OCH_3), 3.74 (s, 3H, OCH_3), 3.46 (t, J=8.8 Hz, 1H, pyrrolidine), 2.14 (s, 3H, NCH_3). HRMS (ESI, m/z): calculated 598.1083 ($C_{28}H_{24}ClFN_4O_4S$ $[M+Na]^+$), found 598.1085.

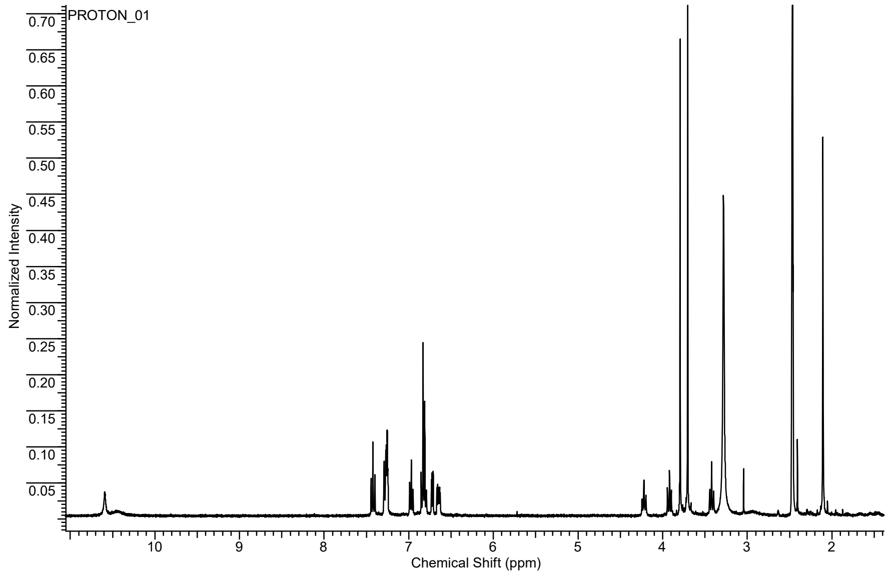

Figure S41. ^{1}H NMR spectra of compound **44**.

1-(3-chloro-4-fluorophenyl)-4'-(3,4-dimethoxyphenyl)-1'-methyl-5'''-bromo-2-thioxodispiro[imidazolidine-4,3'-pyrrolidine-2',3''-indoline]-2'',5-dione (45). Yield 41%, white solid. M.p. 210-212 ºC. ^{1}H NMR (400 MHz, DMSO-d6, δ, ppm): 10.78 (s, 1H, NH-indolinone), 10.68 (s, 1H, NH-imidazolidine), 7.52-7.45 (m, 3H, aryl), 6.89-6.81 (m, 3H, aryl), 6.78-6.73 (m, 2H, aryl), 4.24 (t, J=9.3 Hz, 1H, pyrrolidine), 3.92 (t, J=9.4 Hz, 1H, pyrrolidine), 3.83 (s, 3H, OCH_3), 3.74 (s, 3H, OCH_3), 3.49 (t, J=8.9 Hz, 1H, pyrrolidine), 2.17 (s, 3H, NCH_3). HRMS (ESI, m/z): calculated 555.0087 ($C_{23}H_{20}BrFN_4O_2S$ $[M+K]^+$), found 555.0086.

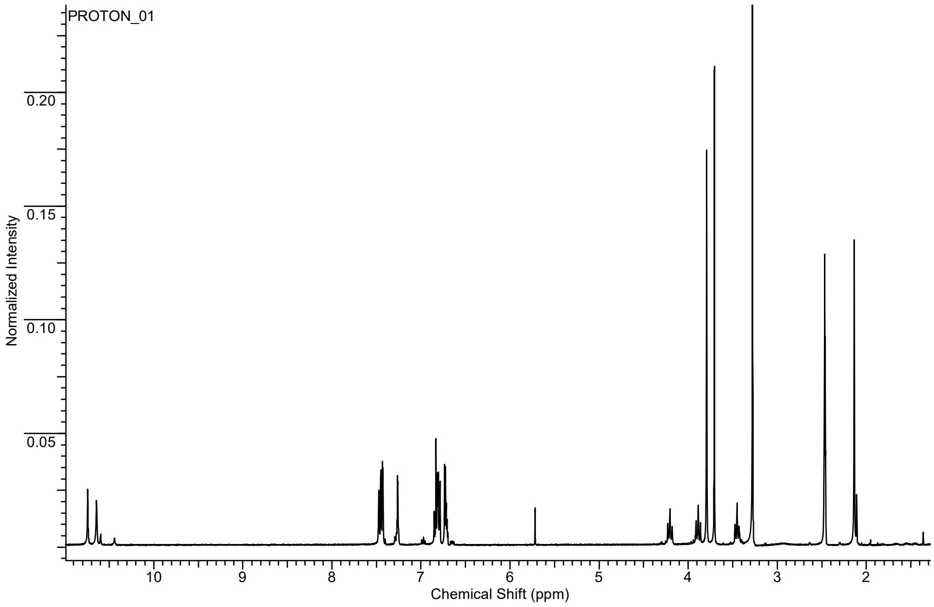

Figure S42. ^{1}H NMR spectra of compound **45**.

1-(3-chloro-4-fluorophenyl)-4'-(4-ethylphenyl)-1'-methyl-2-thiooxodispiro[imidazolidine-4,3'-pyrrolidine-2',3"-indoline]-2",5-dione (46) [2]. Yield 49%, white solid. M.p. 314-315 ^{o}C. ^{1}H NMR (400 MHz, DMSO-d6, δ, ppm): 10.68 (s, 1H, NH-indolinone), 10.35 (s, 1H, NH-imidazolidine), 7.51-7.19 (m, 7H, aryl), 7.15-6.80 (m, 4H, aryl), 4.29 (t, J=9.2 Hz, 1H, pyrrolidine), 3.99 (t, J=9.4 Hz, 1H, pyrrolidine), 3.44 (t, J=8.8 Hz, 1H, pyrrolidine), 2.61 (m, 2H, CH_2CH_3), 2.14 (s, 3H, NCH_3), 1.18 (m, 3H, CH_2CH_3). HRMS (ESI, m/z): calculated 535.1365 ($C_{28}H_{24}ClFN_4O_2S$ $[M+H]^+$), found 535.1370.

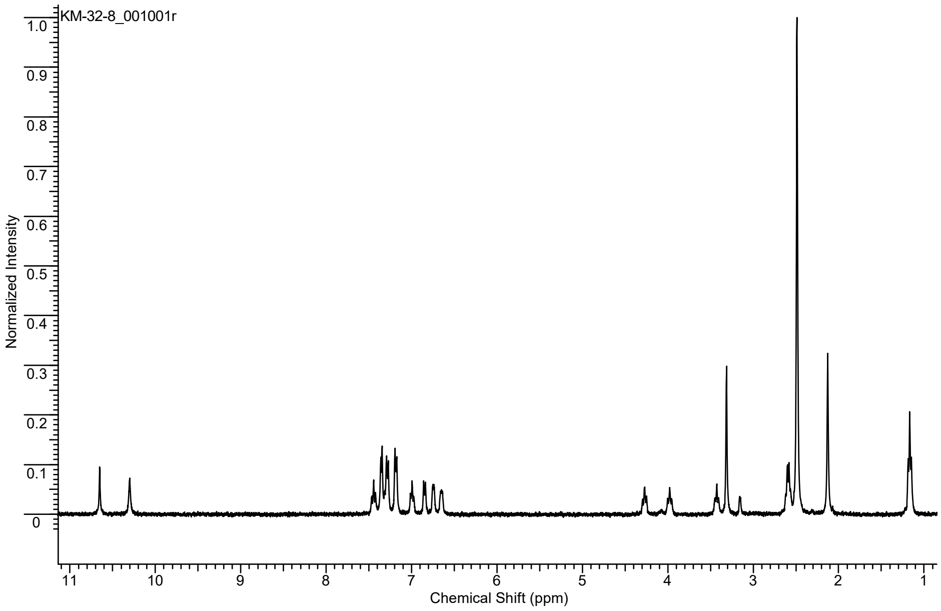

Figure S43. ^{1}H NMR spectra of compound **46**.

1-(3-chloro-4-fluorophenyl)-4’-(4-ethylphenyl)-1’-methyl-5’’’-bromo-2-thiooxodispiro[imidazolidine-4,3’-pyrrolidine-2’,3”-indoline]-2”,5-dione (47) [2]. Yield 48%, white solid. M.p. 210-211 °C. ^{1}H NMR (400 MHz, DMSO-d6, δ, ppm): 10.81 (s, 1H, NH-indolinone), 10.56 (s, 1H, NH-imidazolidine), 7.54-7.42 (m, 3H, aryl), 7.38 (d, J=8.0 Hz, 2H, aryl), 7.20 (d, J=8.0 Hz, 2H, aryl), 6.84 (d, J=8.3 Hz, 1H, aryl), 6.80-6.72 (m, 2H, aryl), 4.27 (t, J=9.2 Hz, 1H, pyrrolidine), 3.96 (t, J=9.4 Hz, 1H, pyrrolidine), 3.47 (t, J=8.7 Hz, 1H, CH, pyrrolidine), 2.62 (m, 2H, CH_2CH_3), 2.17 (s, 3H, NCH_3), 1.18 (m, 3H, CH_2CH_3). HRMS (ESI, m/z): calculated 613.0470 ($C_{28}H_{23}BrClFN_4O_2S$ $[M+H]^+$), found 613.0479.

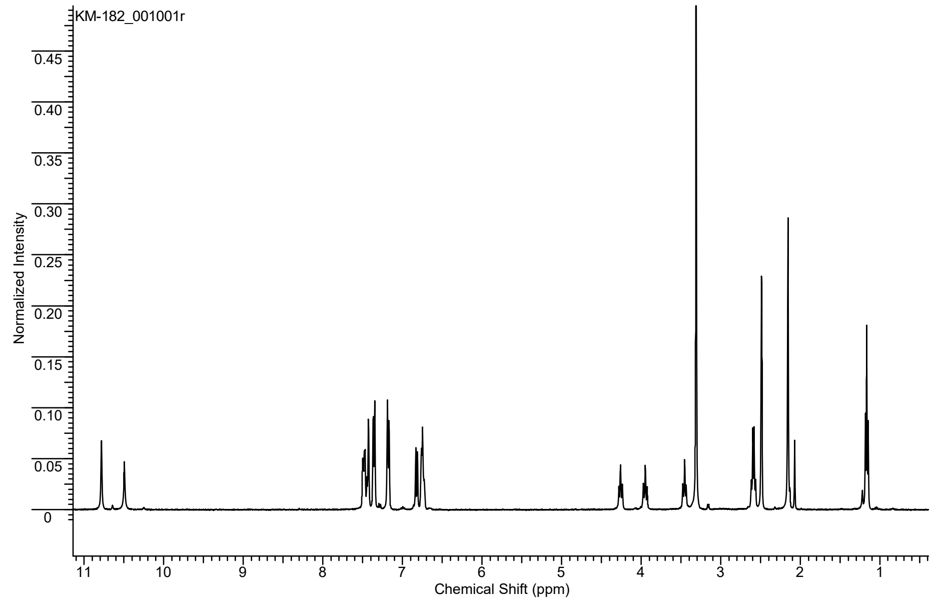

Figure S44. ^{1}H NMR spectra of compound **47**.

1-(3-chloro-4-fluorophenyl)-4’-(3,4-dimethoxyphenyl)-1’-methyl-5’’’-bromo-2-thiooxodispiro[imidazolidine-4,3’-pyrrolidine-2’,3”-indoline]-2”,5-dione (48). Yield 68%, white solid. M.p. 208-209 °C. ^{1}H NMR (400 MHz, DMSO-d6, δ, ppm): 10.78 (s, 1H, NH-indolinone), 10.68 (s, 1H, NH-imidazolidine), 7.52-7.45 (m, 3H, aryl), 6.89-6.81 (m, 3H, aryl), 6.78-6.73 (m, 2H, aryl), 4.24 (t, J=9.3 Hz, 1H, pyrrolidine), 3.92 (t, J=9.4 Hz, 1H, pyrrolidine), 3.83 (s, 3H OCH_3), 3.74 (s, 3H, OCH_3), 3.49 (t, J=8.9 Hz, 1H pyrrolidine), 2.17 (s, 3H, NCH_3). HRMS (ESI, m/z): calculated 555.0087 ($C_{23}H_{20}BrFN_4O_2S$ $[M+K]^+$), found 555.0086.

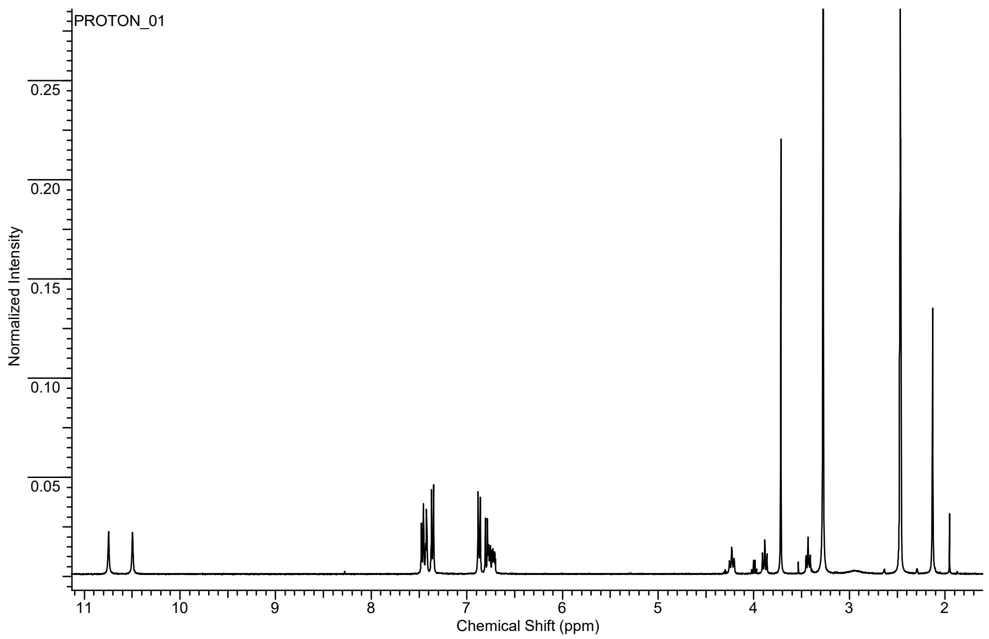

Figure S45. ^{1}H NMR spectra of compound **48**.

1-(3-chloro-4-fluorophenyl)-4’-(4-cyclopentyloxyphenyl)-1’-methyl-5'''-bromo-2-thioxodispiro[imidazolidine-4,3’-pyrrolidine-2’,3’’-indoline]-2’’,5-dione (49). Yield 34%, white solid. M.p. 224-225 °C. ^{1}H NMR (400 MHz, DMSO-d6, δ, ppm): 10.79 (s, 1H, NH-indolinone), 10.57 (s, 1H, NH-imidazolidine), 7.49 (d, J=8.1 Hz, 2H, aryl), 7.45 (s, 1H, aryl), 7.36 (d, J=8.1 Hz, 2H, aryl), 6.90-6.80 (m, 3H, aryl), 6.80-6.72 (m, 2H, aryl), 4.80 (m, 1H, cyclopentyl), 4.25 (t, J=9.3 Hz, 1H, pyrrolidine), 3.90 (t, J=9.1 Hz, 1H, pyrrolidine), 3.45 (t, J=8.9 Hz, 1H, pyrrolidine), 2.16 (s, 3H, NCH_3), 1.96-1.83 (m, 2H, cyclopentyl), 1.76-1.62 (m, 4H, cyclopentyl), 1.62-1.51 (m, 2H, cyclopentyl). HRMS (ESI, m/z): calculated 691.0552 ($C_{31}H_{27}BrClFN_4O_3S$ $[M+Na]^+$), found 691.0547.

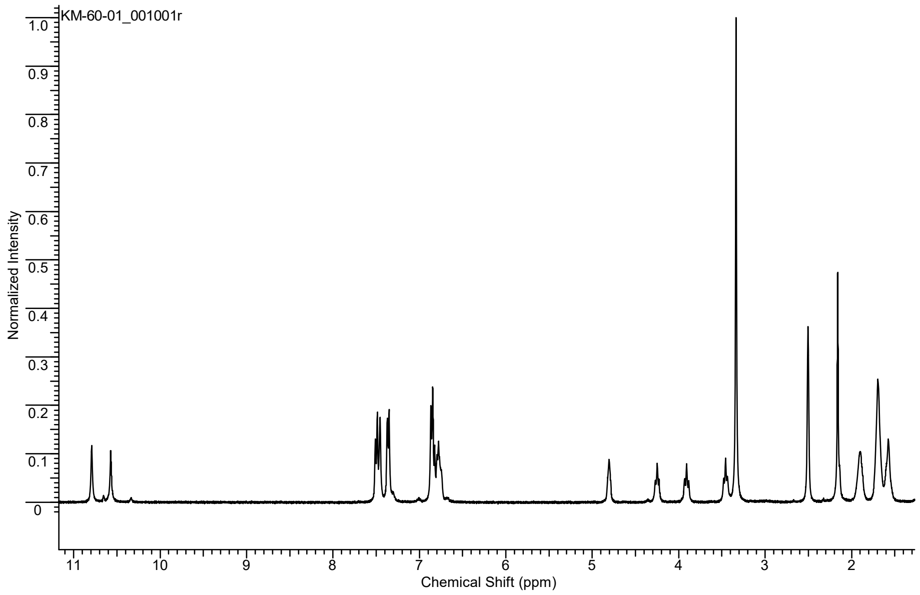

Figure S46. ^{1}H NMR spectra of compound **49**.

1-(4-hydroxyphenyl)-4'-(4-chlorophenyl)-1'-methyl-2-thiooxodispiro[imidazolidine-4,3'-pyrrolidine-2',3''-indoline]-2'',5-dione (50). Yield 94%, white solid. M.p. 195-196 °C. ^{1}H NMR (400 MHz, DMSO-d6, δ, ppm): 10.65 (s, 1H, NH-indolinone), 10.02 (s, 1H, NH-imidazolidine), 9.74 (s, 1H, OH), 7.45 (d, J=8.4 Hz, 2H, aryl), 7.40 (d, J=8.4 Hz, 2H, aryl), 7.32-7.25 (m, 2H, aryl), 6.99 (t, J=7.5 Hz, 1H, aryl), 6.83 (d, J=7.7 Hz, 1H, aryl), 6.68 (d, J=8.7 Hz, 2H, aryl), 6.39 (d, J=8.7 Hz, 2H, aryl), 4.28 (t, J=9.7 Hz, 1H, pyrrolidine), 3.95 (t, J=9.3 Hz, 1H, pyrrolidine), 3.44 (t, J=8.7 Hz, 1H, pyrrolidine), 2.11 (s, 3H, NCH_3). HRMS (ESI, m/z): calculated 505.1096 ($C_{26}H_{21}ClN_4O_3S$ $[M+H]^+$), found 505.1091.

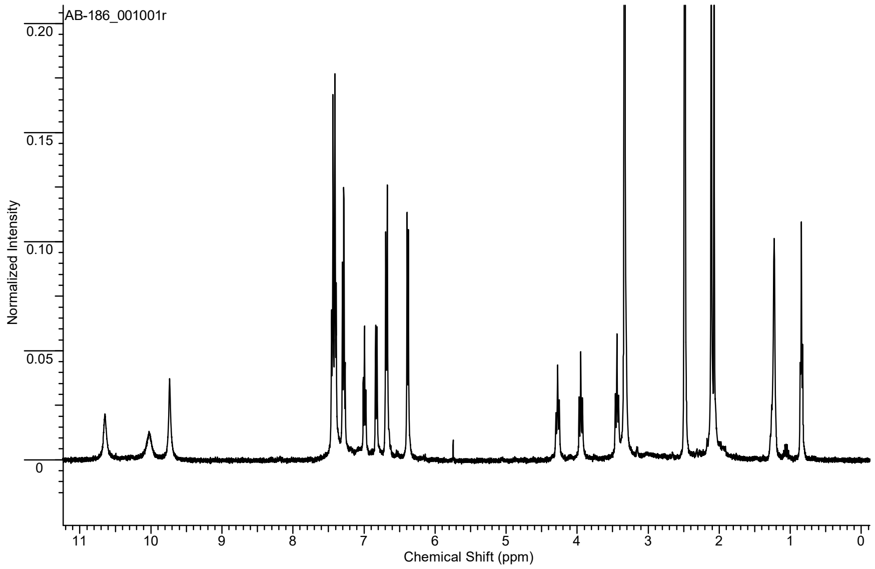

Figure S47. ^{1}H NMR spectra of compound **50**.

1-(4-hydroxyphenyl)-4'-(4-chlorophenyl)-1'-methyl-5'''-bromo-2-thiooxodispiro[imidazolidine-4,3'-pyrrolidine-2',3''-indoline]-2'',5-dione (51). Yield 91%, white solid. M.p. 228-229 °C. ^{1}H NMR (400 MHz, DMSO-d6, δ, ppm): 10.79 (s, 1H, NH-indolinone), 10.30 (s, 1H, NH-imidazolidine), 9.77 (s, 1H, OH), 7.48-7.36 (m, 6H, aryl), 6.80 (d, J=8.3 Hz, 1H, aryl), 6.71 (d, J=8.4 Hz, 2H, aryl), 6.43 (d, J=8.6 Hz, 2H, aryl), 4.27 (t, J=9.3 Hz, 1H, pyrrolidine), 3.92 (t, J=3.9 Hz, 1H, pyrrolidine), 3.47 (t, J=8.6 Hz, 1H, pyrrolidine), 2.14 (s, 3H, NCH_3). HRMS (ESI, m/z): calculated 583.0201 ($C_{26}H_{20}BrClN_4O_3S$ $[M+H]^+$), found 583.0203.

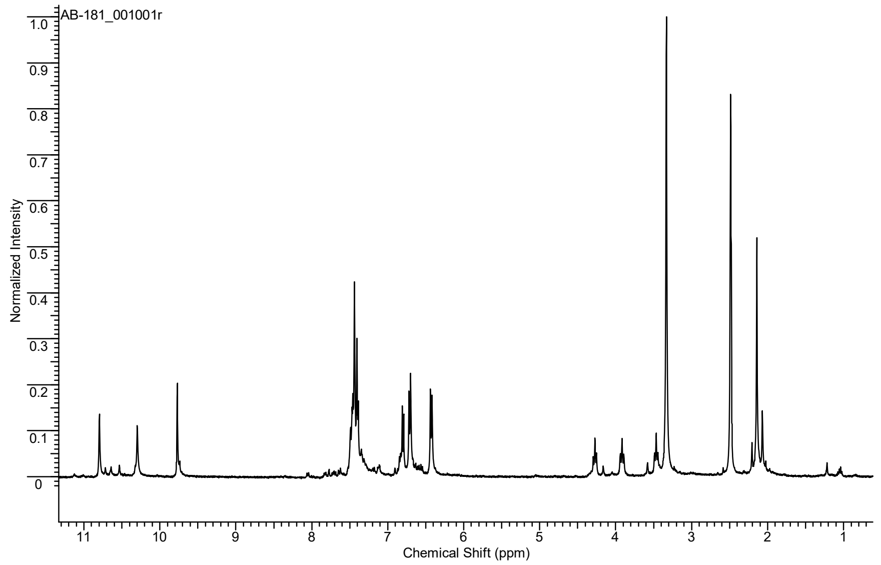

Figure S48. ^{1}H NMR spectra of compound **51**.

1-(4-hydroxyphenyl)-4’-(4-chlorophenyl)-1’-methyl-5’’’-chloro-2-thiooxodispiro[imidazolidine-4,3’-pyrrolidine-2’,3”-indoline]-2”,5-dione (52). Yield 88%, white solid. M.p. 216-217 °C. ^{1}H NMR (400 MHz, DMSO-d6, δ, ppm): 10.82 (s, 1H, NH-indolinone), 10.31 (s, 1H, NH-imidazolidine), 9.80 (s, 1H, OH), 7.47-7.32 (m, 6H, aryl), 6.85 (d, J=7.8 Hz, 1H, aryl), 6.71 (d, J=7.8 Hz, 2H, aryl), 6.42 (d, J=8.4 Hz, 2H, aryl), 4.29 (t, J=8.8 Hz, 1H, pyrrolidine), 3.91 (t, J=9.8 Hz, 1H, pyrrolidine), 3.47 (t, J=7.6 Hz, 1H, pyrrolidine), 2.14 (s, 3H, NCH_3). HRMS (ESI, m/z): calculated 539.0706 ($C_{26}H_{20}Cl_2N_4O_3S$ $[M+H]^+$), found 539.0707.

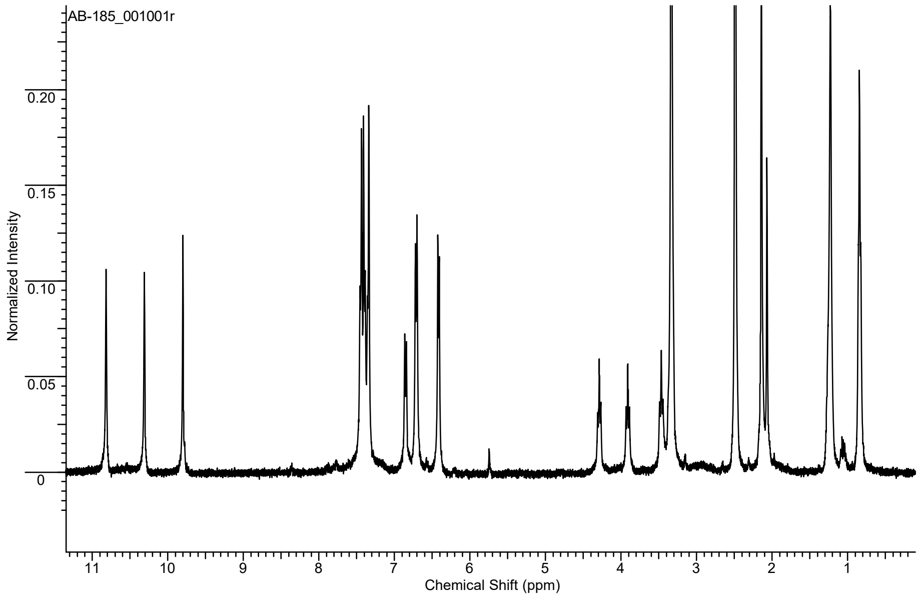

Figure S49. ^{1}H NMR spectra of compound **52**.

1-(4-hydroxyphenyl)-4’-(2-chlorophenyl)-1’-methyl-2-thiooxodispiro[imidazolidine-4,3’-pyrrolidine-2’,3”-indoline]-2”,5-dione (53). Yield 75%, white solid. M.p. 198-199 °C. ^{1}H NMR (400

MHz, DMSO-d6, δ, ppm): 10.67 (s, 1H, NH-indolinone), 9.75 (s, 1H, NH-imidazolidine), 9.55 (s, 1H, OH), 7.95 (d, J=7.7 Hz, 1H, aryl), 7.45 (t, J=8.3 Hz, 2H, aryl), 7.34 (d, J=7.7 Hz, 2H, aryl), 7.17 (d, J=7.6 Hz, 1H, aryl), 7.00 (t, J=7.7 Hz, 1H, aryl), 6.83 (d, J=7.7 Hz, 1H, aryl), 6.72 (d, J=8.3 Hz, 2H, aryl), 6.39 (d, J=8.3 Hz, 2H, aryl), 4.55 (d, J=8.3 Hz, 1H, pyrrolidine), 4.23 (d, J=9.4 Hz, 1H, pyrrolidine), 3.43 (t, J=7.8 Hz, 1H, pyrrolidine), 2.12 (s, 3H, NCH_3). HRMS (ESI, m/z): calculated 505.1096 ($C_{26}H_{21}ClN_4O_3S$ $[M+H]^+$), found 505.1098.

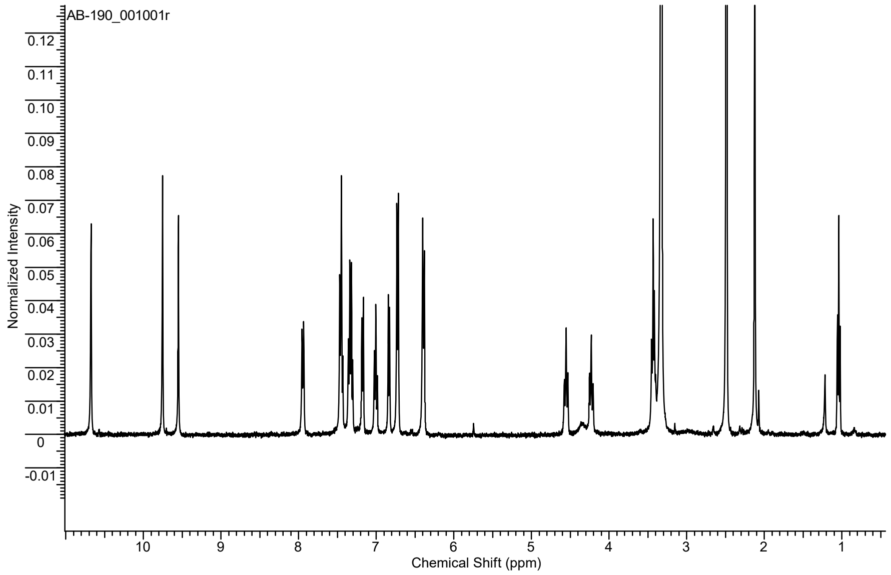

Figure S50. ^1H NMR spectra of compound **53**.

1-(4-hydroxyphenyl)-4’-(2-chlorophenyl)-1’-methyl-5’’’-bromo-2-thiooxodispiro[imidazolidine-4,3’-pyrrolidine-2’,3”-indoline]-2”,5-dione (54). Yield 76%, white solid. M.p. 241-242 °C. ^1H NMR (400 MHz, DMSO-d6, δ, ppm): 10.85 (s, 1H, NH-indolinone), 9.84 (s, 1H, NH-imidazolidine), 9.52 (s, 1H, OH), 7.92 (d, J=7.7 Hz, 1H, aryl), 7.54 (d, J=8.3 Hz, 1H, aryl), 7.49-7.42 (m, 2H, aryl), 7.34 (t, J=7.7 Hz, 1H, aryl), 7.25 (s, 1H, aryl), 6.82 (d, J=8.2 Hz, 1H, aryl), 6.76 (d, J=7.8 Hz, 2H, aryl), 6.44 (d, J=8.4 Hz, 2H, aryl), 4.53 (t, J=8.3 Hz, 1H, pyrrolidine), 4.22 (t, J=8.8 Hz, 1H, pyrrolidine), 3.45 (t, J=6.5 Hz, 1H, pyrrolidine), 2.15 (s, 3H, NCH_3). HRMS (ESI, m/z): calculated 583.0201 ($C_{26}H_{20}BrClN_4O_3S$ $[M+H]^+$), found 583.0201.

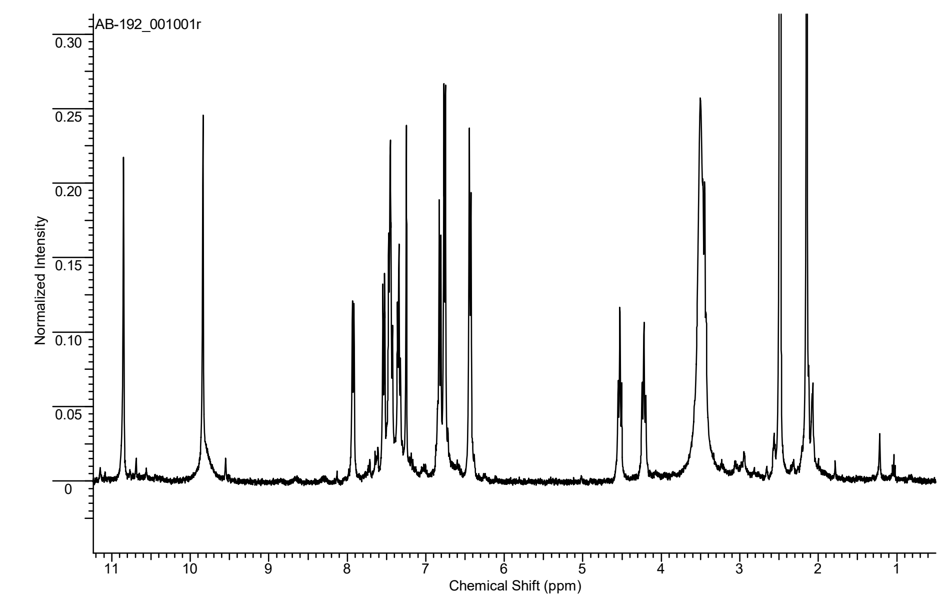

Figure S51. ^{1}H NMR spectra of compound **54**.

1-(4-hydroxyphenyl)-4'-(2-chlorophenyl)-1'-methyl-5'''-chloro-2-thiooxodispiro[imidazolidine-4,3'-pyrrolidine-2',3''-indoline]-2'',5-dione (55). Yield 75%, white solid. M.p. 205-206 ºC. ^{1}H NMR (400 MHz, DMSO-d6, δ, ppm): 10.84 (s, 1H, NH-indolinone), 9.83 (s, 1H, NH-imidazolidine), 9.54 (s, 1H, OH), 7.91 (d, J=7.7 Hz, 1H, aryl), 7.50-7.31 (m, 4H, aryl), 7.12 (s, 1H, aryl), 6.86 (d, J=8.3 Hz, 1H, aryl), 6.75 (d, J=7.9 Hz, 2H, aryl), 6.42 (d, J=8.1 Hz, 2H, aryl), 4.54 (t, J=8.3 Hz, 1H, pyrrolidine), 4.22 (t, J=9.2 Hz, 1H, pyrrolidine), 3.45 (t, J=8.3 Hz, 1H, pyrrolidine), 2.15 (s, 3H, NCH_3). HRMS (ESI, m/z): calculated 539.0706 ($C_{26}H_{20}Cl_2N_4O_3S$ $[M+H]^+$), found 539.0709.

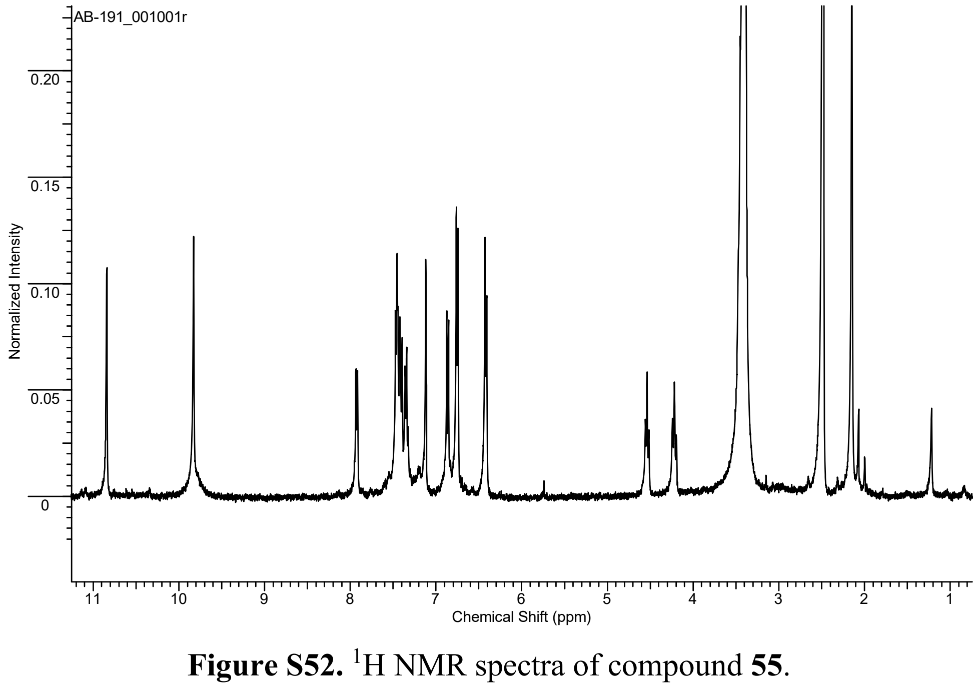

Figure S52. ^{1}H NMR spectra of compound **55**.

1-(4-hydroxyphenyl)-4’-(3,4-dimethoxyphenyl)-1’-methyl-5’’’-bromo-2-thiooxodispiro[imidazolidine-4,3’-pyrrolidine-2’,3”-indoline]-2”,5-dione (56). Yield 69%, white solid. M.p. 197-198 °C. ^{1}H NMR (400 MHz, DMSO-d6, δ, ppm): 10.77 (s, 1H, NH-indolinone), 10.27 (s, 1H, NH-imidazolidine), 9.55 (s, 1H, OH), 7.50-7.44 (m, 2H, aryl), 7.22 (s, 1H, aryl), 6.89-6.75 (m, 3H, aryl), 6.72-6.66 (m, 2H, aryl), 6.39 (d, J=8.7 Hz, 2H, aryl), 4.51 (t, J=8.2 Hz, 1H, pyrrolidine), 4.19 (t, J=8.7 Hz, 1H, pyrrolidine), 3.90 (t, J=9.4 Hz, 1H, pyrrolidine), 3.80 (s, 3H, OCH_3), 3.72 (s, 3H, OCH_3), 2.14 (s, 3H, NCH_3). HRMS (ESI, m/z): calculated 609.0802 ($C_{28}H_{25}BrN_4O_5S$ $[M+H]^+$), found 609.0803.

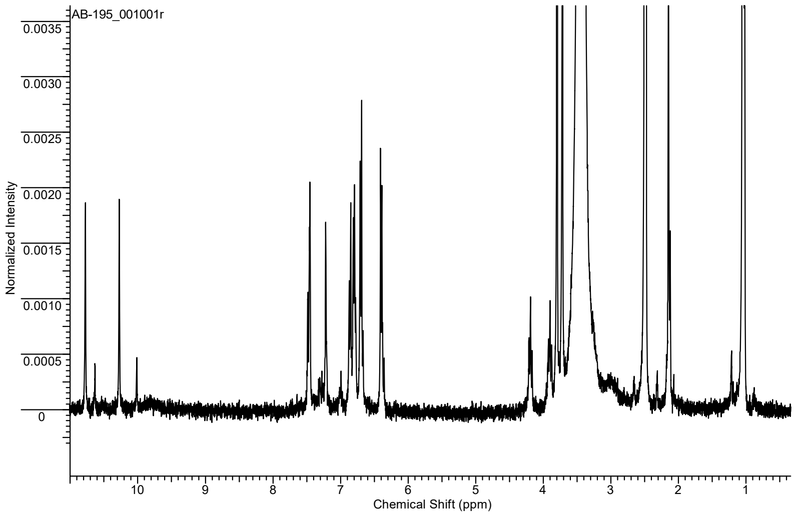

Figure S53. ^{1}H NMR spectra of compound **56**.

1-(4-hydroxyphenyl)-4’-(3,4-dimethoxyphenyl)-1’-methyl-5’’’-chloro-2-thiooxodispiro[imidazolidine-4,3’-pyrrolidine-2’,3”-indoline]-2”,5-dione (57). Yield 53%, white solid. M.p. 205-206 °C. ^{1}H NMR (400 MHz, DMSO-d6, δ, ppm): 10.75 (s, 1H, NH-indolinone), 10.32 (s, 1H, NH-imidazolidine), 9.52 (s, 1H, OH), 7.32-7.38 (m, 2H, aryl), 7.22 (s, 1H, aryl), 6.85 (t, J=8.20 Hz, 2H, aryl), 6.81-6.77 (m, 1H, aryl), 6.68 (d, J=7.5 Hz, 2H, aryl), 6.38 (d, J=7.5 Hz, 2H, aryl), 4.49 (t, J=8.2 Hz, 1H, pyrrolidine), 4.21 (t, J=9.1 Hz, 1H, pyrrolidine), 3.90 (t, J=8.1 Hz, 1H, pyrrolidine), 3.80 (s, 3H, OCH_3), 3.72 (s, 3H, OCH_3), 2.15 (s, 3H, NCH_3). HRMS (ESI, m/z): calculated 565.1307 ($C_{28}H_{25}ClN_4O_5S$ $[M+H]^+$), found 565.1307.

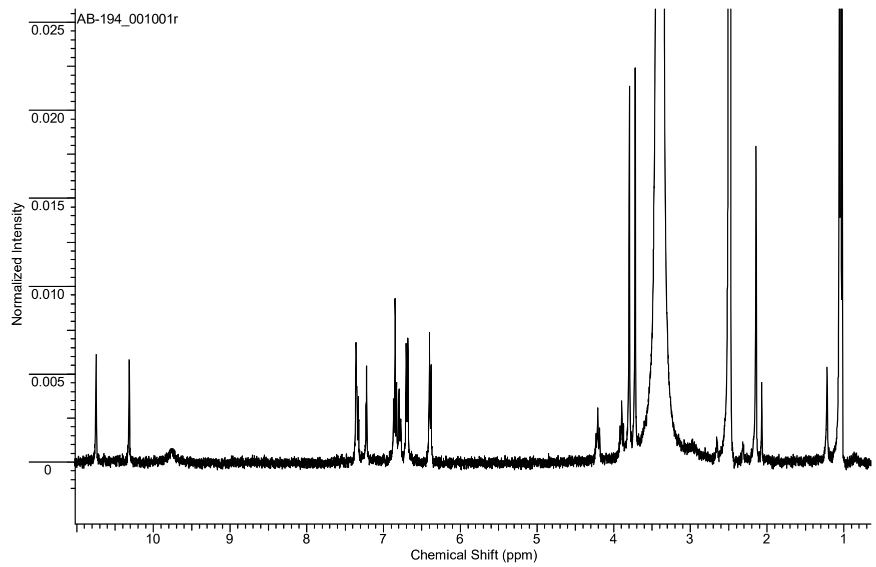

Figure S54. ^{1}H NMR spectra of compound **57**.

1-(3-chloro-4-methoxyphenyl)-4'-(3-chlorophenyl)-1'-methyl-2-thioxodispiro[imidazolidine-4,3'-pyrrolidine-2',3''-indoline]-2'',5-dione (58). Yield 54%, white solid. M.p. 217-218 °C. ^{1}H NMR (400 MHz, DMSO-d6, δ, ppm): 10.67 (s, 1H, NH-indolinone), 10.38 (s, 1H, NH-imidazolidine), 7.57 (s, 1H, aryl), 7.43-7.35 (m, 3H, aryl), 7.32 (t, J=7.8 Hz, 1H, aryl), 7.27 (d, J=7.5 Hz, 1H, aryl), 7.14 (d, J=8.8 Hz, 1H, aryl), 7.00 (t, J=7.5 Hz, 1H, aryl), 6.87 (d, J=7.7 Hz, 1H, aryl), 6.62 (d, J=2.1 Hz, 1H, aryl), 6.59 (dd, J_1=2.3 Hz, J_2=8.7 Hz, 1H, aryl), 4.31 (t, J=9.3 Hz, 1H, pyrrolidine),3.98 (t, J=9.4 Hz, 1H, pyrrolidine), 3.85 (s, 3H, OCH_3), 3.48 (t, J=8.4 Hz, 1H, pyrrolidine), 2.14 (s, 3H, NCH_3). HRMS (ESI, m/z): calculated 575.0682 ($C_{27}H_{22}Cl_2N_4O_3S$ $[M+Na]^+$), found 575.0677.

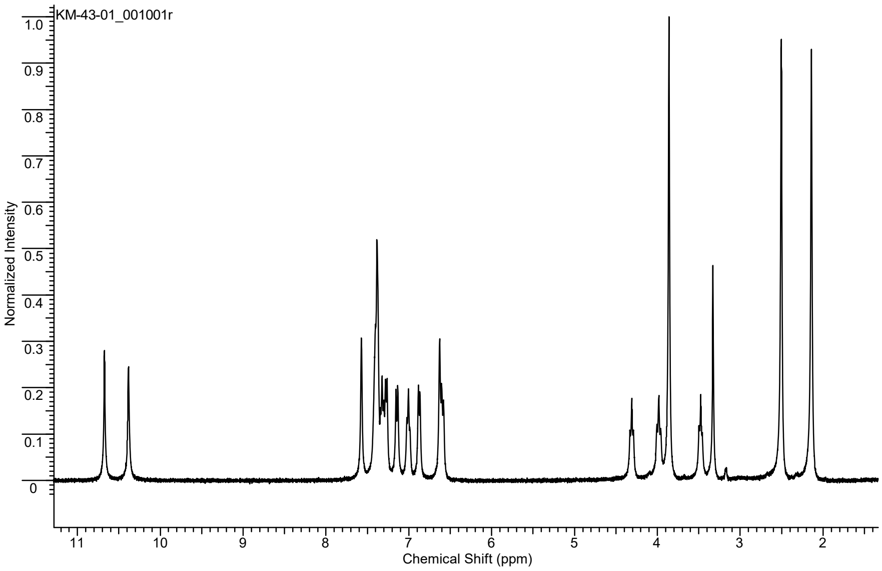

Figure S55. ^{1}H NMR spectra of compound **58**.

1-(3-chloro-4-methoxyphenyl)-4'-(3-chlorophenyl)-1'-methyl-5'''-bromo-2-thioxodispiro[imidazolidine-4,3'-pyrrolidine-2',3''-indoline]-2'',5-dione (59). Yield 51%, white solid. M.p. 215-216 °C. ^{1}H NMR (400 MHz, DMSO-d6, δ, ppm): 10.81 (s, 1H, NH-indolinone), 10.57 (s, 1H, NH-imidazolidine), 7.57 (s, 1H, aryl), 7.51 (dd, J_1=2.2 Hz, J_2=8.3 Hz, 1H, aryl), 7.40-7.42 (m, 2H, aryl), 7.39-7.36 (m, 2H, aryl),7.16 (d, J=9.0 Hz, 1H, aryl), 6.84 (d, J=8.3 Hz, 1H, aryl), 6.68 (dd, J_1=2.5 Hz, J_2=8.8 Hz, 1H, aryl), 6.63 (d, J=2.6 Hz, 1H, aryl), 4.30 (t, J=9.1 Hz, 1H, pyrrolidine), 3.94 (t, J=9.4 Hz, 1H, pyrrolidine), 3.87 (s, 3H OCH_3), 3.49 (t, J=8.6 Hz, 1H, pyrrolidine), 2.16 (s, 3H, NCH_3). HRMS (ESI, m/z): calculated 652.9787 ($C_{27}H_{21}BrCl_2N_4O_3S$ $[M+Na]^+$), found 652.9782.

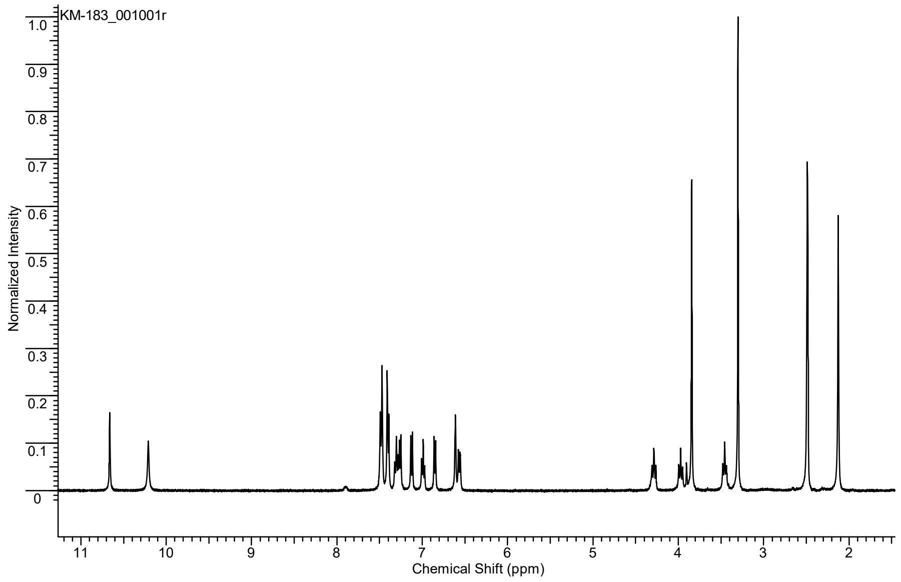

Figure 56. ^{1}H NMR spectra of compound **59**.

1-(4-fluorophenyl)-4'-(4-ethoxyphenyl)-1'-methyl-2-thioxodispiro[imidazolidine-4,3'-pyrrolidine-2',3''-indoline]-2'',5-dione (60). Yield 38%, white solid. M.p. 225-226 °C. ^{1}H NMR (400 MHz, DMSO-d6, δ, ppm): 10.64 (s, 1H, NH-indolinone), 10.08 (s, 1H, NH-imidazolidine), 7.38-7.32 (m, 3H, aryl), 7.29 (t, J=7.6 Hz, 1H, aryl), 7.22 (t, J=8.7 Hz, 2H, aryl), 7.01 (t, J=7.5 Hz, 1H, aryl), 6.89 (d, J=8.6 Hz, 2H, aryl), 6.85 (d, J=7.6 Hz, 2H, aryl), 6.66 (dd, J_1=4.9 Hz, J_2=8.8 Hz, 2H, aryl), 4.28 (t, J=9.1 Hz, 1H, pyrrolidine), 4.01 (q, J=7.0 Hz, CH_2CH_3), 3.94 (t, J=9.5 Hz, 1H, pyrrolidine), 3.43 (t, J=8.0 Hz, 1H, pyrrolidine), 2.14 (s, 3H, NCH_3), 1.32 (t, J=7.0 Hz, 3H, CH_2CH_3). HRMS (ESI, m/z): calculated 517.1704 ($C_{28}H_{25}FN_4O_3S$ $[M+H]^+$), found 517.1698.

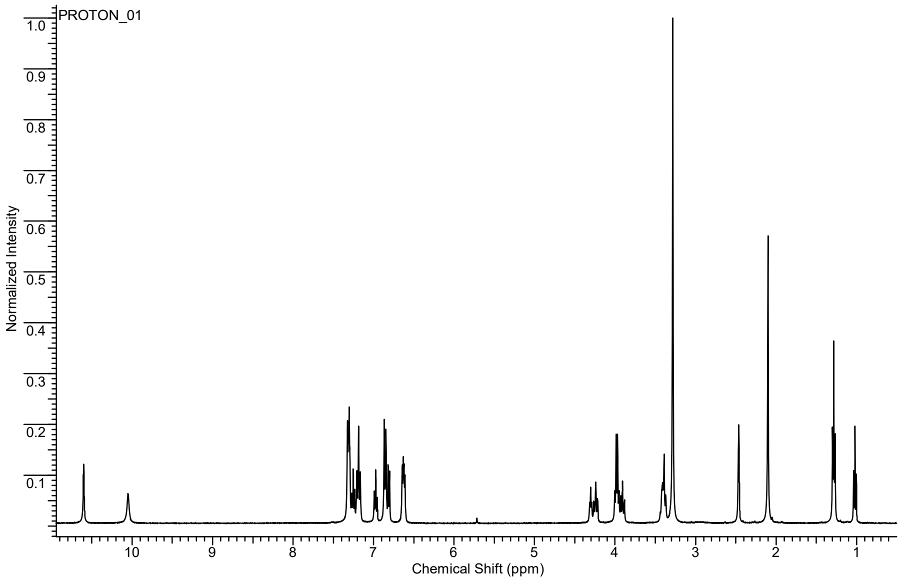

Figure S57. ^{1}H NMR spectra of compound **60**.

1-(4-fluorophenyl)-4'-(4-ethoxyphenyl)-1'-methyl-5'''-bromo-2-thioxodispiro[imidazolidine-4,3'-pyrrolidine-2',3''-indoline]-2'',5-dione (61). Yield 36%, white solid. M.p. 210-211 °C. ^{1}H NMR (400 MHz, DMSO-d6, δ, ppm): 10.77 (s, 1H, NH-indolinone), 10.42 (s, 1H, NH-imidazolidine), 7.52-7.46 (m, 2H, aryl), 7.35 (d, J=8.7 Hz, 1H, aryl), 7.24 (t, J=8.7 Hz, 2H, aryl), 6.89 (d, J=8.7 Hz, 2H, aryl), 6.81 (d, J=8.1 Hz, 1H, aryl), 6.70 (dd, J_1=4.9 Hz, J_2=8.9 Hz, 2H, aryl), 4.27 (t, J=9.4 Hz, 1H, pyrrolidine), 4.01 (q, J=6.8 Hz, 2H, CH_2CH_3), 3.90 (t, J=9.4 Hz, 1H, pyrrolidine), 3.46 (t, J=8.7 Hz, 1H, pyrrolidine), 2.16 (s, 3H, NCH_3), 1.31 (t, J=7.0 Hz, 3H, CH_2CH_3). HRMS (ESI, m/z): calculated 597.0790 ($C_{28}H_{24}BrFN_4O_3S$ $[M+H]^+$), found 597.0781.

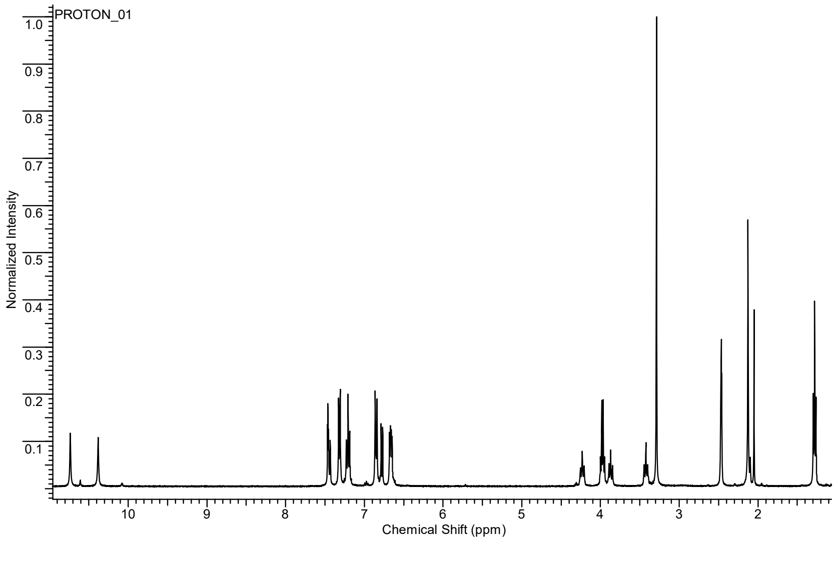

Figure S58. ^{1}H NMR spectra of compound **61**.

1-(1-phenylethyl)-4’-(2-chlorophenyl)-1’-methyl-5’’’-bromo-2-thiooxodispiro[imidazolidine-4,3’-pyrrolidine-2’,3”-indoline]-2”,5-dione (62). Yield 89%, white solid. M.p. 209-210 °C. ^{1}H NMR (400 MHz, DMSO-d6, δ, ppm): 10.79 (s, 1H, NH-indolinone), 9.74 (s, 1H, NH-imidazolidine), 7.99 (d, J=7.8 Hz, 1H, aryl), 7.52-7.17 (m, 10H, aryl), 6.79 (d, J=8.3 Hz, 1H, aryl), 5.63 (q, J=7.3 Hz, 1H, CH-CH_3), 4.37 (t, J=8.7 Hz, 1H, pyrrolidine), 4.05 (m, 1H, pyrrolidine), 3.37 (t, J=8.6 Hz, 1H, pyrrolidine), 2.11 (s, 3H, NCH_3), 1.40 (d, J=7.2 Hz, 3H, CH(Ph)-CH_3) HRMS (ESI, m/z): calculated 595.0565 ($C_{28}H_{24}BrClN_4O_2S$ $[M+H]^+$), found 595.0566.

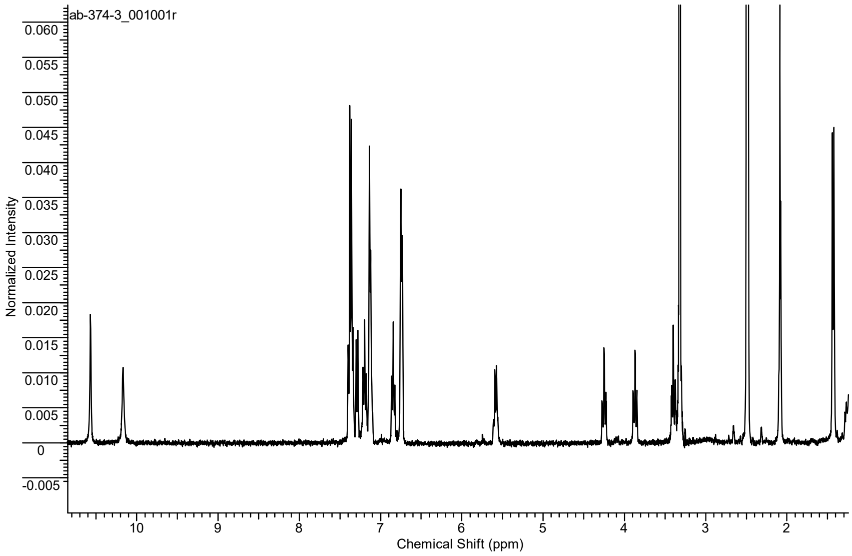

Figure S59. ^{1}H NMR spectra of compound **62**.

1-(1-phenylethyl)-4’-(3-chlorophenyl)-1’-methyl-5’’’-bromo-2-thiooxodispiro[imidazolidine-4,3’-pyrrolidine-2’,3”-indoline]-2”,5-dione (63). Yield 53%, white solid. M.p. 221-222 °C. ^{1}H NMR (400 MHz, DMSO-d6, δ, ppm): 10.66 (s, 1H, NH-indolinone), 10.29 (s, 1H, NH-imidazolidine), 7.55 (s, 1H, aryl), 7.41-7.35 (m, 6H, aryl), 7.30 (m, 1H, aryl), 7.01 (d, J=7.5 Hz, 1H, aryl), 6.86 (d, J=7.6 Hz, 1H, aryl), 6.67-6.62 (m, 2H, aryl), 5.59 (q, J=7.2 Hz, 1H, CH(Ph)), 4.33 (t, J=9.0 Hz, 1H, pyrrolidine), 3.98 (t, J=9.4 Hz, 1H, pyrrolidine), 3.47 (t, J=8.6 Hz, 1H, pyrrolidine), 2.14 (s, 3H, NCH_3), 1.31 (d, J=7.1 Hz, 3H, CH(Ph)-CH3). HRMS (ESI, m/z): calculated 569.0229 ($C_{26}H_{20}BrClN_4O_2S$ $[M+H]^+$), found 569.0231.

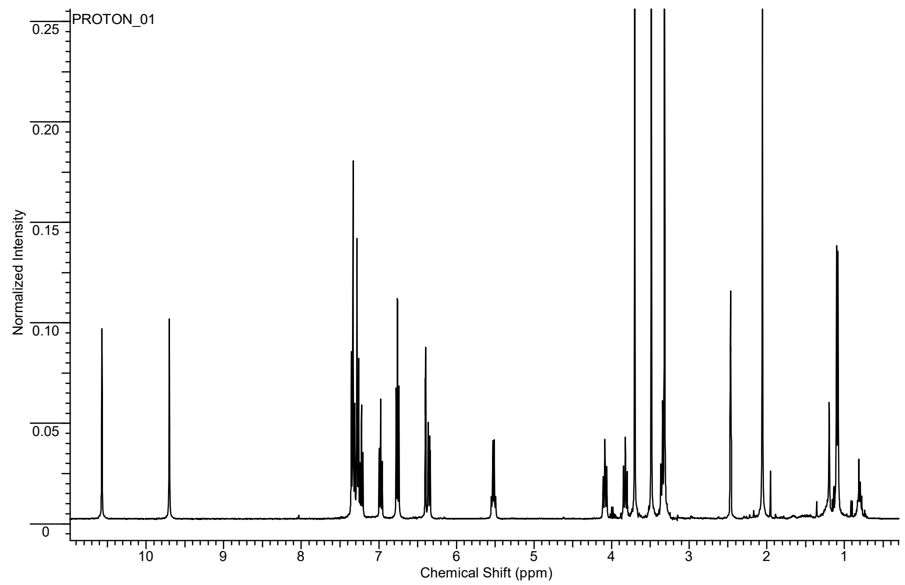

Figure S60. ^{1}H NMR spectra of compound **63**.

1-(1-phenylethyl)-4'-(2-chlorophenyl)-1'-methyl-5'''-chloro-2-thiooxodispiro[imidazolidine-4,3'-pyrrolidine-2',3''-indoline]-2'',5-dione (64). Yield 83%, white solid. M.p. 208-209 °C. ^{1}H NMR (400 MHz, DMSO-d6, δ, ppm): 10.86 (s, 1H, NH-indolinone), 10.17 (s, 1H, NH-imidazolidine), 7.92 (d, J=7.7 Hz, 1H, aryl), 7.60-7.29 (m, 6H, aryl), 7.10 (s, 1H, aryl), 6.87 (d, J=8.3 Hz, 1H, aryl), 6.67 (d, J=8.4 Hz, 2H, aryl), 5.60 (q, J=7.2 Hz, 1H, CH(Ph)), 4.56 (t, J=8.8 Hz, 1H, pyrrolidine), 4.25 (t, J=9.2 Hz, 1H, pyrrolidine), 3.47 (t, J=8.6 Hz, 1H, pyrrolidine), 2.16 (s, 3H, NCH_3), 1.32 (d, J=7.1 Hz, 3H, CH(Ph)-CH3). HRMS (ESI, m/z): calculated 557.0367 ($C_{26}H_{19}Cl_3N_4O_2S$ $[M+H]^+$), found 557.0364.

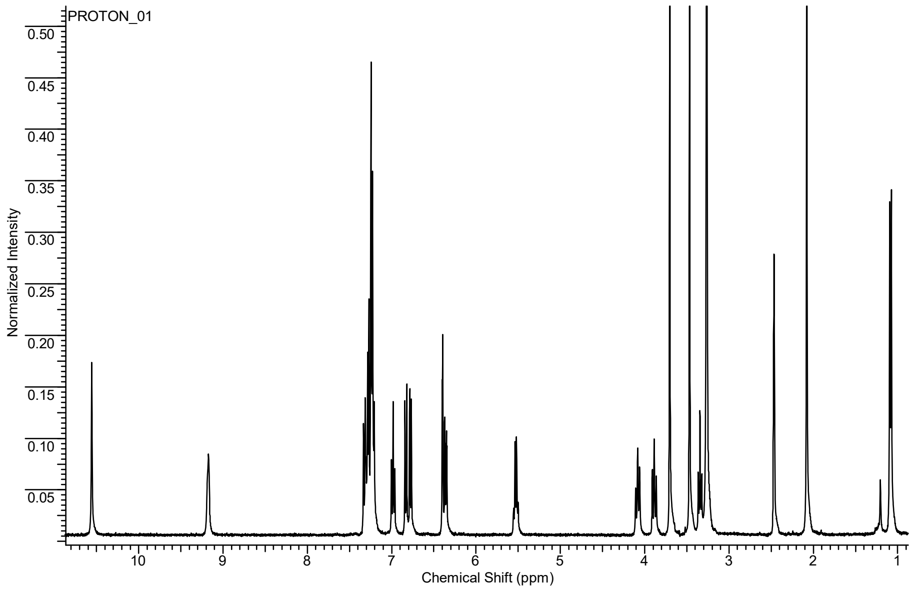

Figure S61. ^{1}H NMR spectra of compound **64**.

1-(1-phenylethyl)-4'-(4-chlorophenyl)-1'-methyl-5'''-bromo-2-thiooxodispiro[imidazolidine-4,3'-pyrrolidine-2',3''-indoline]-2'',5-dione (65). Yield 65%, white solid. M.p. 210-211 °C. ^{1}H NMR

(400 MHz, DMSO-d6, δ, ppm): 10.75 (s, 1H, NH-indolinone), 10.38 (s, 1H, NH-imidazolidine), 7.49-7.42 (m, 7H, aryl), 7.34 (d, J=8.4 Hz, 2H, aryl), 6.91-6.84 (m, 2H, aryl), 6.77 (d, J=8.1 Hz, 1H, aryl), 5.60 (q, J=7.2 Hz, 1H, CH(Ph)), 4.08 (t, J=9.05 Hz, 1H, pyrrolidine), 3.86 (t, J=9.7 Hz, 1H, pyrrolidine), 3.40 (t, J=8.4 Hz, 1H, pyrrolidine), 2.12 (s, 3H, NCH_3), 1.33 (d, J=7.1 Hz, 3H, CH(Ph)-CH3). HRMS (ESI, m/z): calculated 595.0565 ($C_{28}H_{24}BrClN_4O_2S$ $[M+H]^+$), found 595.0573.

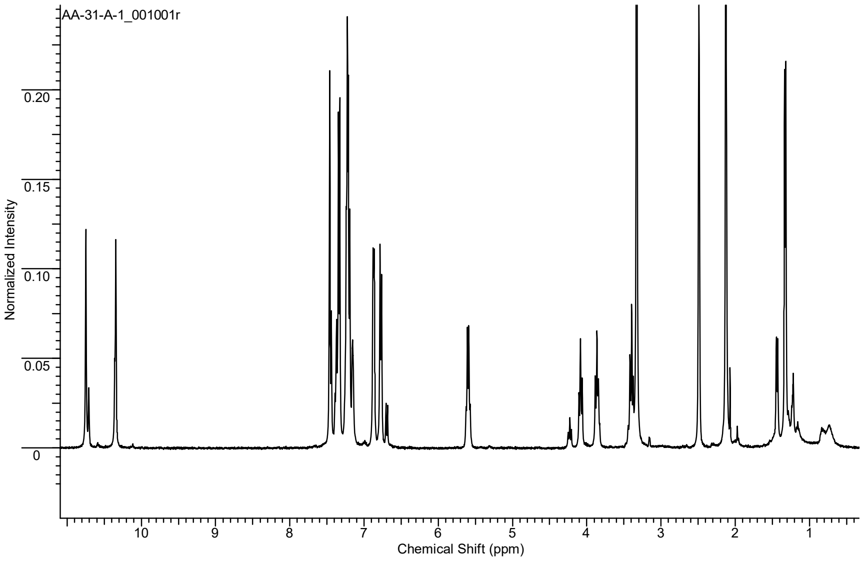

Figure S62. ^{1}H NMR spectra of compound **65**.

1-(1-phenylethyl)-4'-(4-chlorophenyl)-1'-methyl-5'''-chloro-2-thiooxodispiro[imidazolidine-4,3'-pyrrolidine-2',3''-indoline]-2'',5-dione (66). Yield 62%, white solid. M.p. 217-218 °C. ^{1}H NMR (400 MHz, DMSO-d6, δ, ppm): 10.73 (s, 1H, NH-indolinone), 10.42 (s, 1H, NH-imidazolidine), 7.42-7.13 (m, 9H, aryl), 6.90-6.74 (m, 3H, aryl), 5.59 (q, J=7.6 Hz, 1H, CH(Ph)), 4.10 (t, J=9.2 Hz, 1H, pyrrolidine), 3.85 (t, J=9.8 Hz, 1H, pyrrolidine), 3.40 (t, J=8.3 Hz, 1H, pyrrolidine), 2.12 (s, 3H, NCH_3), 1.31 (d, J=7.1 Hz, 3H, CH(Ph)-CH3). HRMS (ESI, m/z): calculated 551.1070 ($C_{28}H_{24}Cl_2N_4OS$ $[M+H]^+$), found 551.1081.

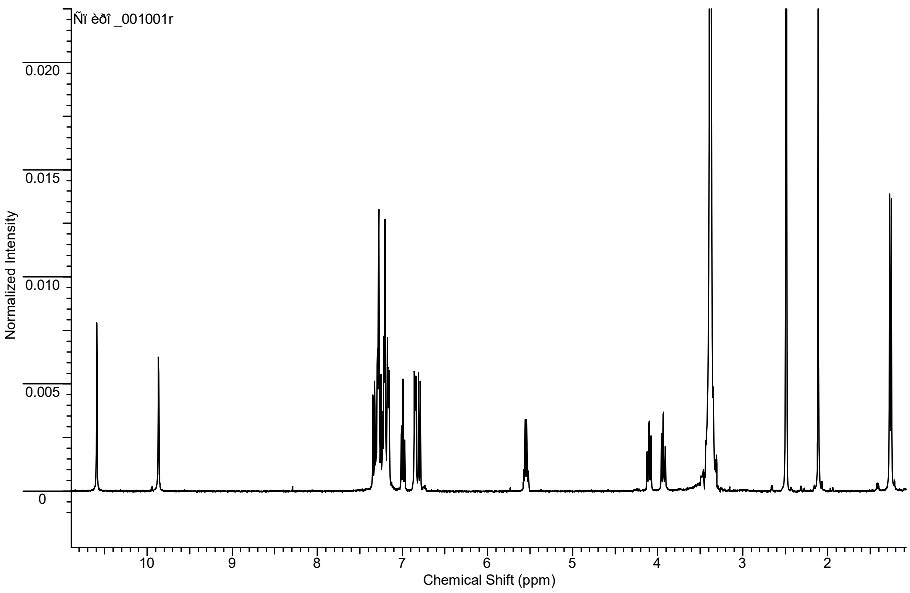

Figure S63. ^{1}H NMR spectra of compound **66**.

1-allyl-4'-(pyridin-2-yl)-1'-methyl-2-thioxodispiro[imidazolidine-4,3'-pyrrolidine-2',3''-indoline]-2'',5-dione (67). Yield 12%, white solid. M.p. 185-186 ºC. ^{1}H NMR (400 MHz, $CDCl_3$, δ, ppm): δ 8.78 (s, 1H, NH-indolinone), 8.70 (s, 1H, NH-imidazolidine), 8.61 (d, J=4.5 Hz, 1H, H_{α}-Py), 7.77 (td, J_1=1.5 Hz, J_2=7.7 Hz, 1H, H_{β}-Py), 7.49 (d, J=7.7, 2H, $H_{\beta'}$-Py), 7.25-7.19 (m, 2H, aryl), 7.03 (td, J_1=0.7 Hz, J_2=7.6 Hz, 1H, aryl), 6.73 (d, J=7.7 Hz, 1H, aryl), 5.42 (m, 1H, CH), 4.85 (d, J=10.4 Hz, 1H, CH_2), 4.60-4.49 (m, 2H, CH_2), 4.35 (dd, J_1=8.8 Hz, J_2=10.6 Hz, 1H, pyrrolidine), 4.24 (d, J=5.0 Hz, 2H, pyrrolidine), 3.58 (t, J=8.1 Hz, 1H, pyrrolidine), 2.25 (s, 3H, NCH_3). HRMS (ESI, m/z): calculated 420.1489 ($C_{22}H_{21}N_5O_2S$ $[M+H]^+$), found 420.1479.

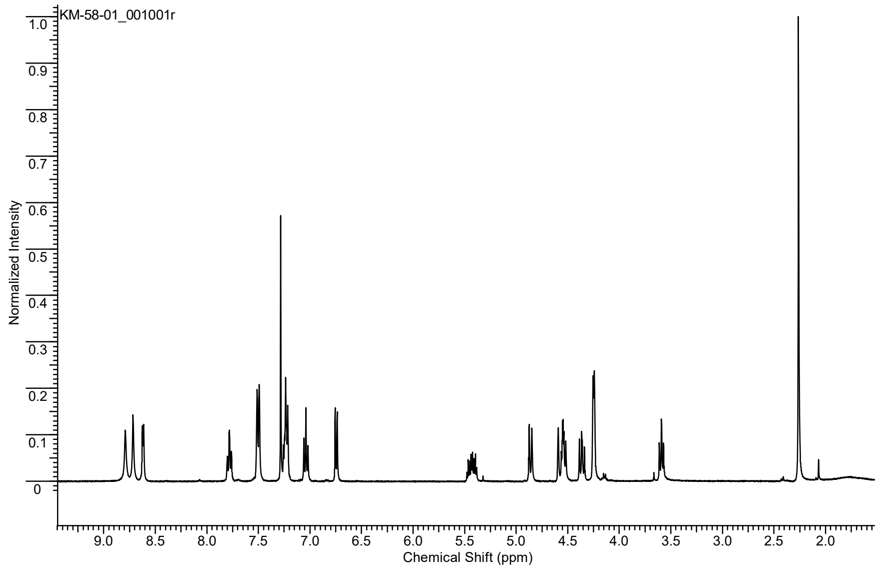

Figure S64. ^{1}H NMR spectra of compound **67**.

1-allyl-4'-(3-chlorophenyl)-1'-methyl-2-thioxodispiro[imidazolidine-4,3'-pyrrolidine-2',3''-indoline]-2'',5-dione (68). Yield 12%, white solid. M.p. 179-180 °C. ^{1}H NMR (400 MHz, DMSO-d6, δ, ppm): 10.56 (s, 1H, NH-indolinone), 10.07 (s, 1H, NH-imidazolidine), 7.43 (s, 1H, aryl), 7.37 (d, J=7.4 Hz, 1H, aryl), 7.34-7.28 (m, 3H, aryl), 7.24 (td, J_1=0.8 Hz, J_2=7.6 Hz, 1H, aryl), 6.98 (td, J_1=0.5 Hz, J_2=7.6 Hz, 1H, aryl), 6.79 (d, J=7.4 Hz, 1H, aryl), 5.35 (m, 1H, CH), 4.78 (dd, J_1=1.0 Hz, J_2=10.6, 1H, CH_2), 4.34 (dd, J_1=1.2 Hz, J_2=17.0 Hz, 1H, CH_2),4.25 (t, J=9.2 Hz, 1H, pyrrolidine), 4.03-3.99 (m, 2H, CH_2), 3.89 (t, J=9.5 Hz, 1H, pyrrolidine), 3.42 (t, J=8.7 Hz, 1H, pyrrolidine), 2.10 (s, 3H, NCH_3). HRMS (ESI, m/z): calculated 453.1147 ($C_{23}H_{21}ClN_4O_2S$ $[M+H]^+$), found 453.1146.

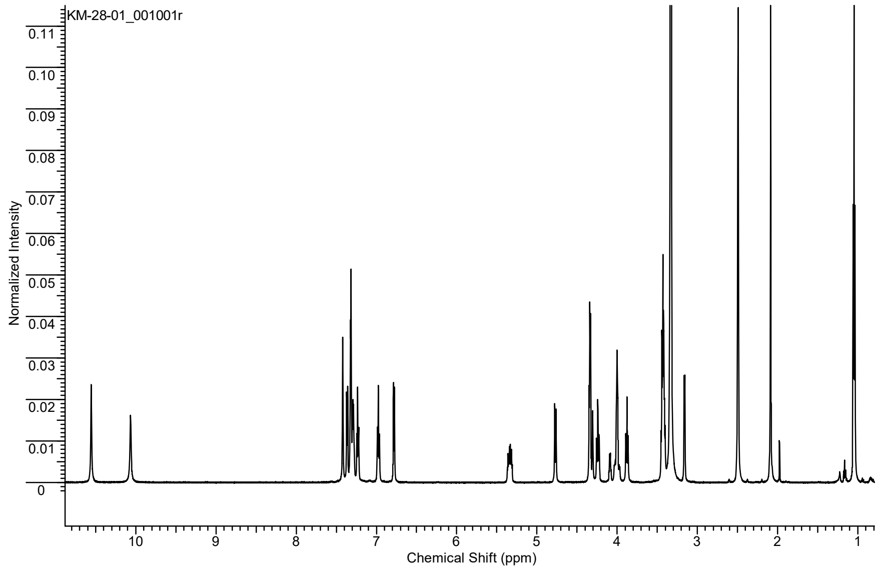

Figure S65. ^{1}H NMR spectra of compound **68**.

1-(3-chloro-4-methoxyphenyl)-4'-(3-chlorophenyl)-1'-methyl-1'''-(prop-2-yn-1-yl)-2-thioxodispiro[imidazolidine-4,3'-pyrrolidine-2',3''-indoline]-2'',5-dione (69). Yield 36%, white solid. M.p. 199-200 °C. ^{1}H NMR (400 MHz, DMSO-d6, δ, ppm): 10.64 (s, 1H, NH-imidazolidine), 7.57 (s, 1H, aryl), 7.48-7.36 (m, 5H, aryl), 7.18-7.10 (m, 3H, aryl), 6.64 (d, J=2.4 Hz, 1H, aryl), 6.60 (dd, J_1=2.4 Hz, J_2=8.7 Hz, 1H, aryl), 4.62-4.50 (m, 2H, CH2C≡CH), 4.39 (t, J=9.2 Hz, 1H, pyrrolidine), 3.96 (t, J=9.3 Hz, 1H, pyrrolidine), 3.85 (s, 3H, OCH_3), 3.52 (t, J=9.0 Hz, 1H, pyrrolidine), 3.24 (t, J=2.2 Hz, 1H, C≡CH), 2.10 (s, 3H, NCH_3). HRMS (ESI, m/z): calculated ($C_{30}H_{25}Cl_2N_4O_3S$ $[M+H]^+$), 591.1019, found 591.1022.

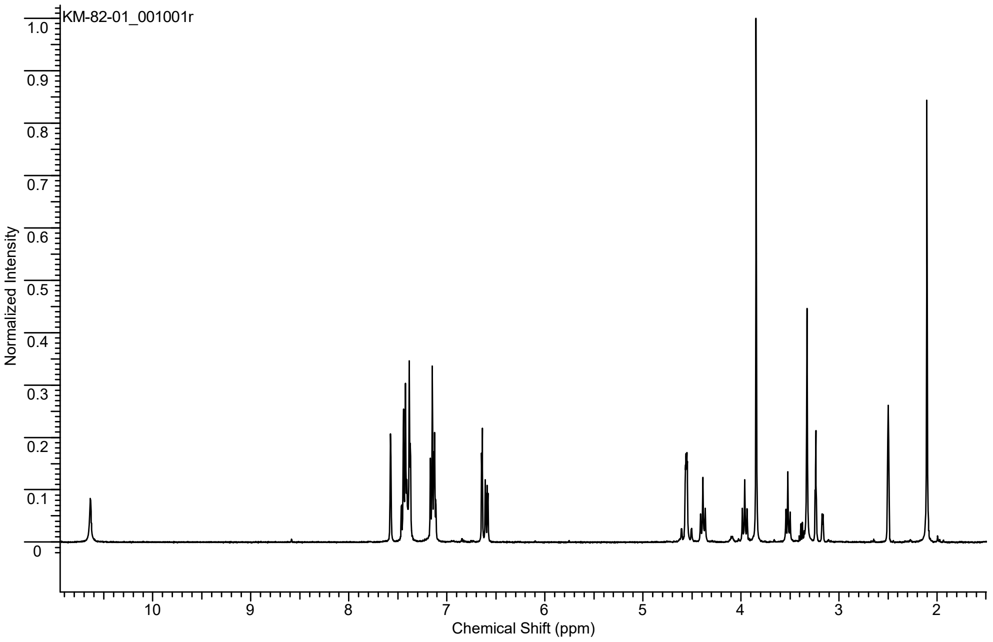

Figure S66. ^{1}H NMR spectra of compound **69**.

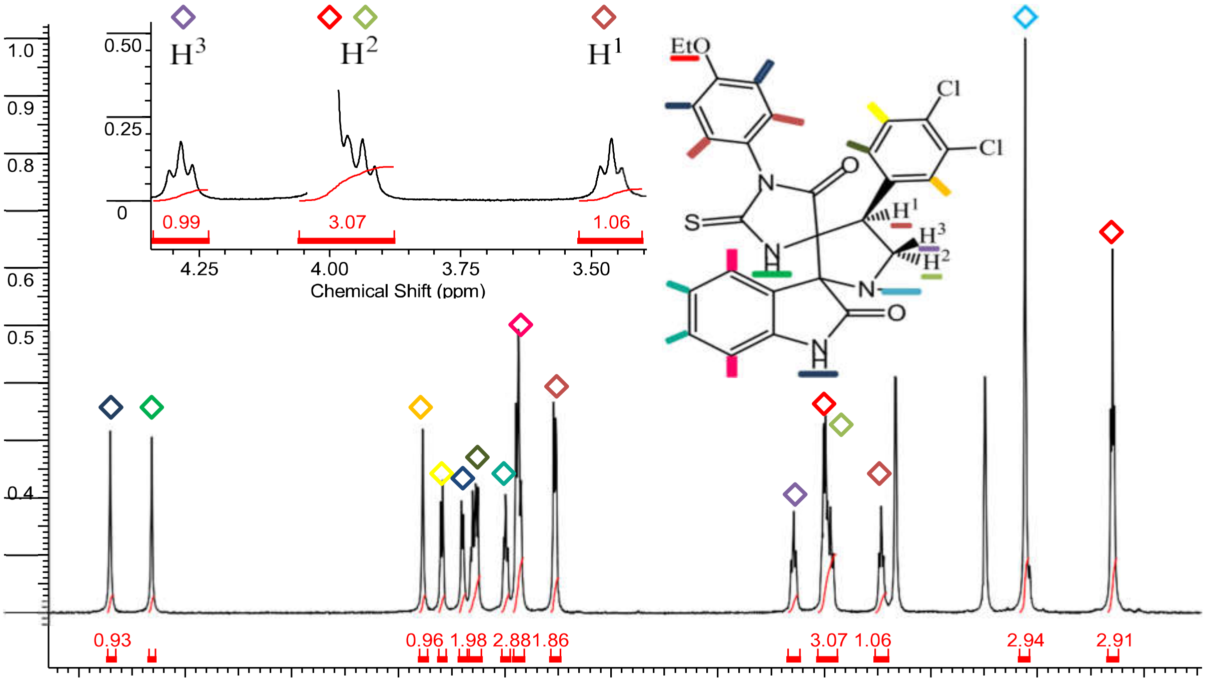

Figure S70. ^{1}H NMR spectrum of dispiro-indolinone **27**

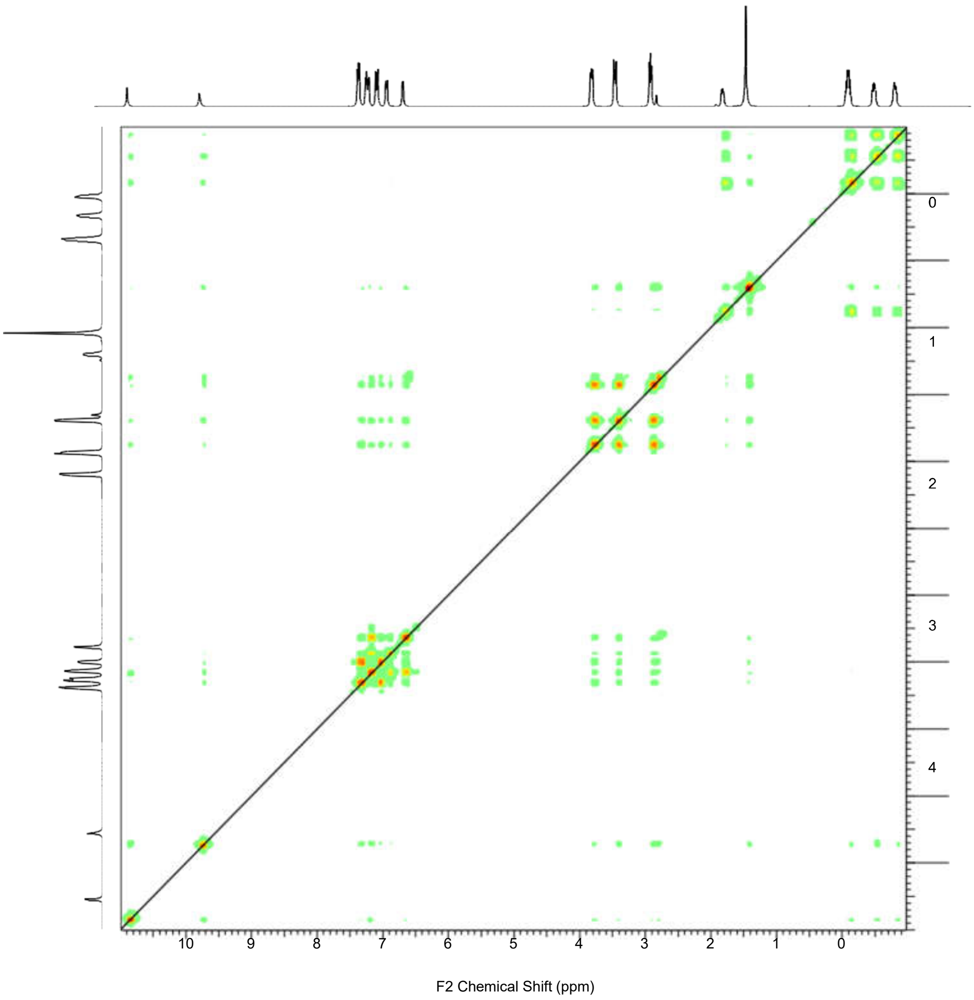

Figure S71. ^{1}H-^{1}H COSY NMR spectrum of *dispiro*-indolinone **27**

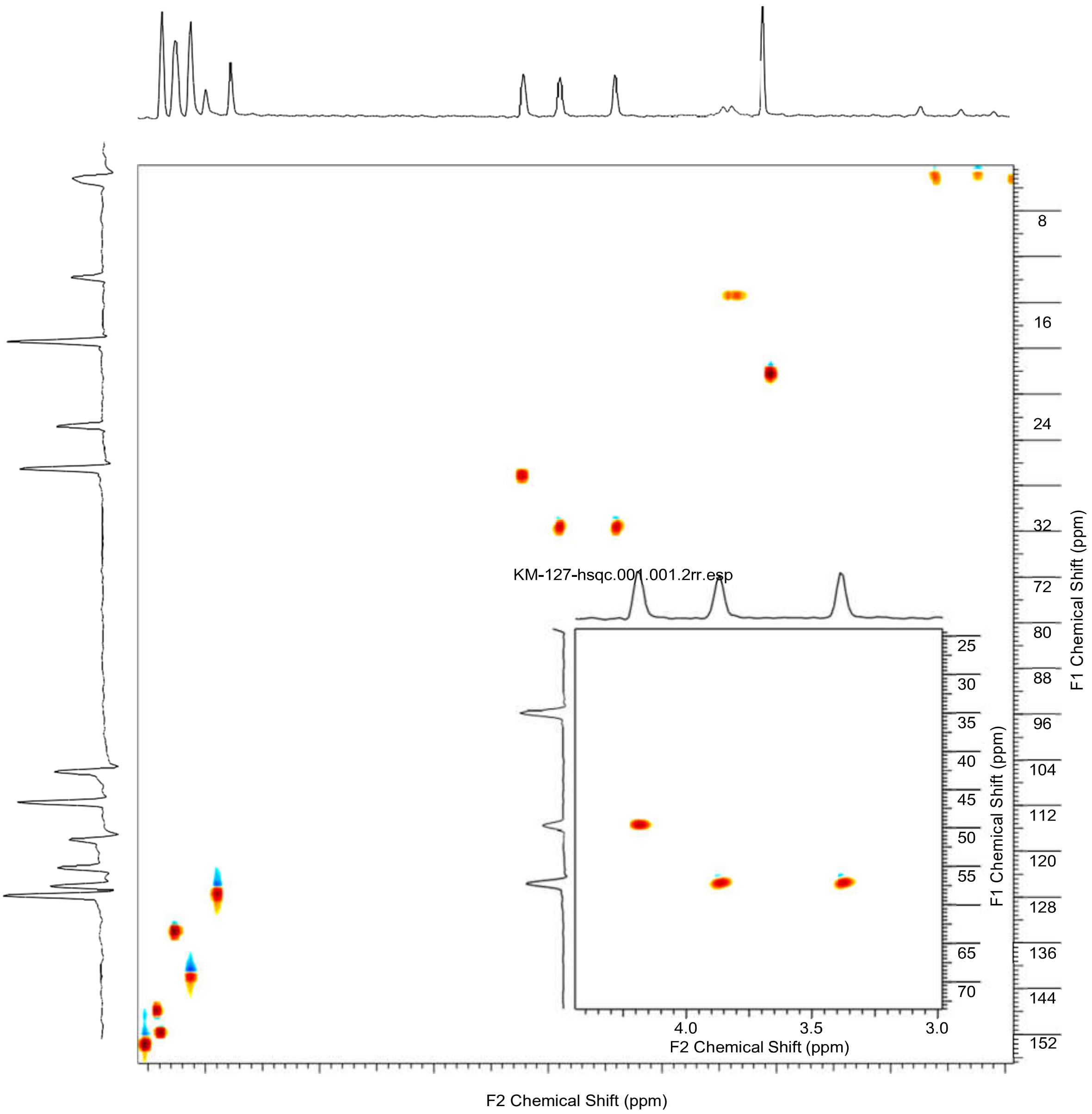

Figure S72. ^{1}H-^{13}C HSQC spectrum of *dispiro*-indolinone **27**

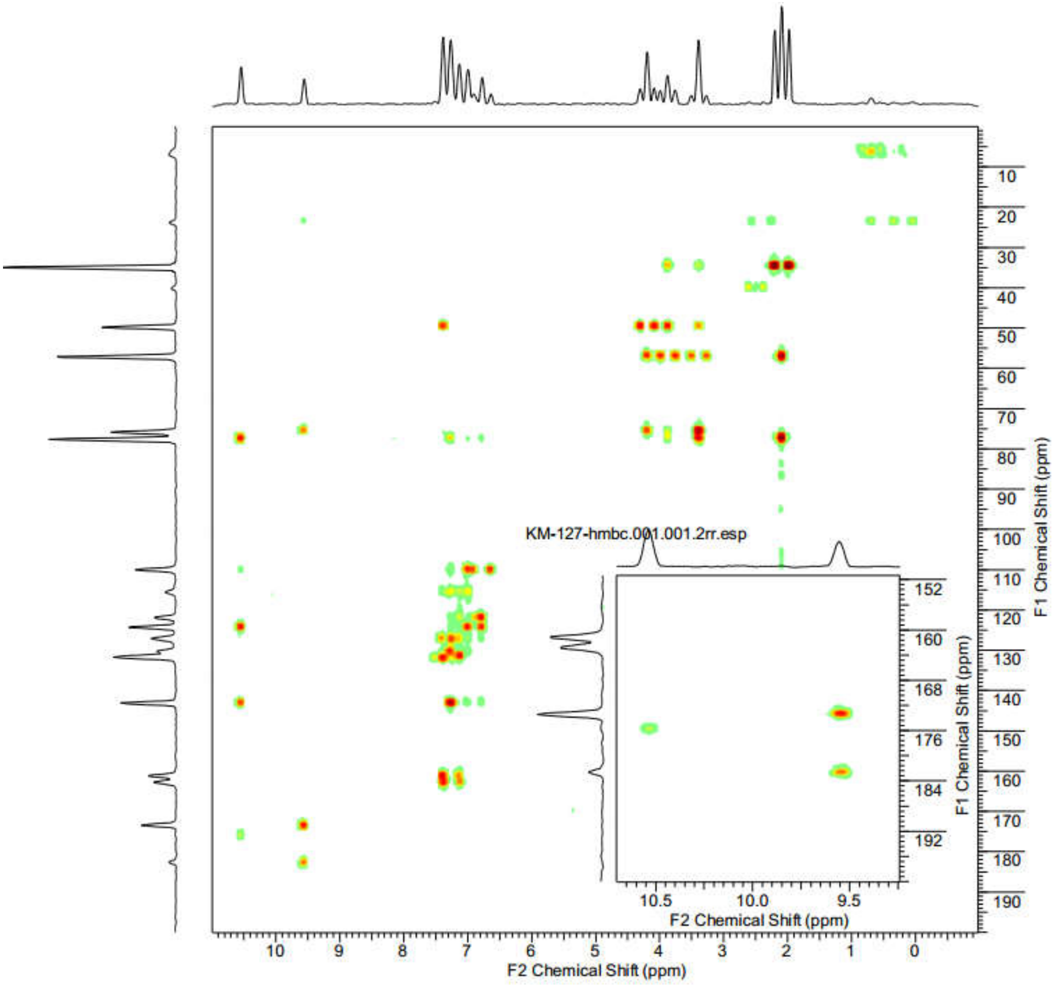

Figure S73. ^{1}H-^{13}C HMBC spectrum of *dispiro*-indolinone **27**

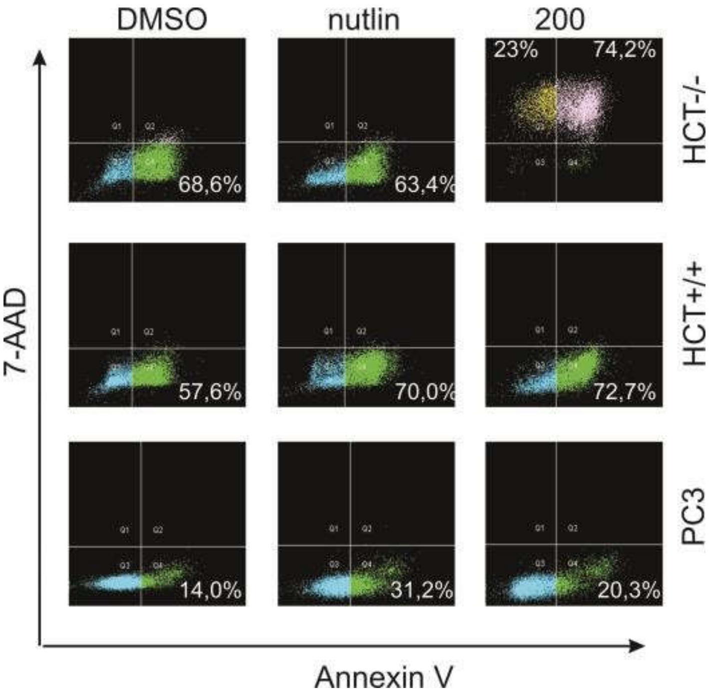

Figure S74. The mechanism of cell death (necrosis/apoptosis) caused by compound 12 by flow cytometry staining with 7AAD/AnnexinV. HCT116p53-/- mainly has necrosis/late apoptosis, HCT116wt has predominantly early apoptosis, and PC3 also has early apoptosis.

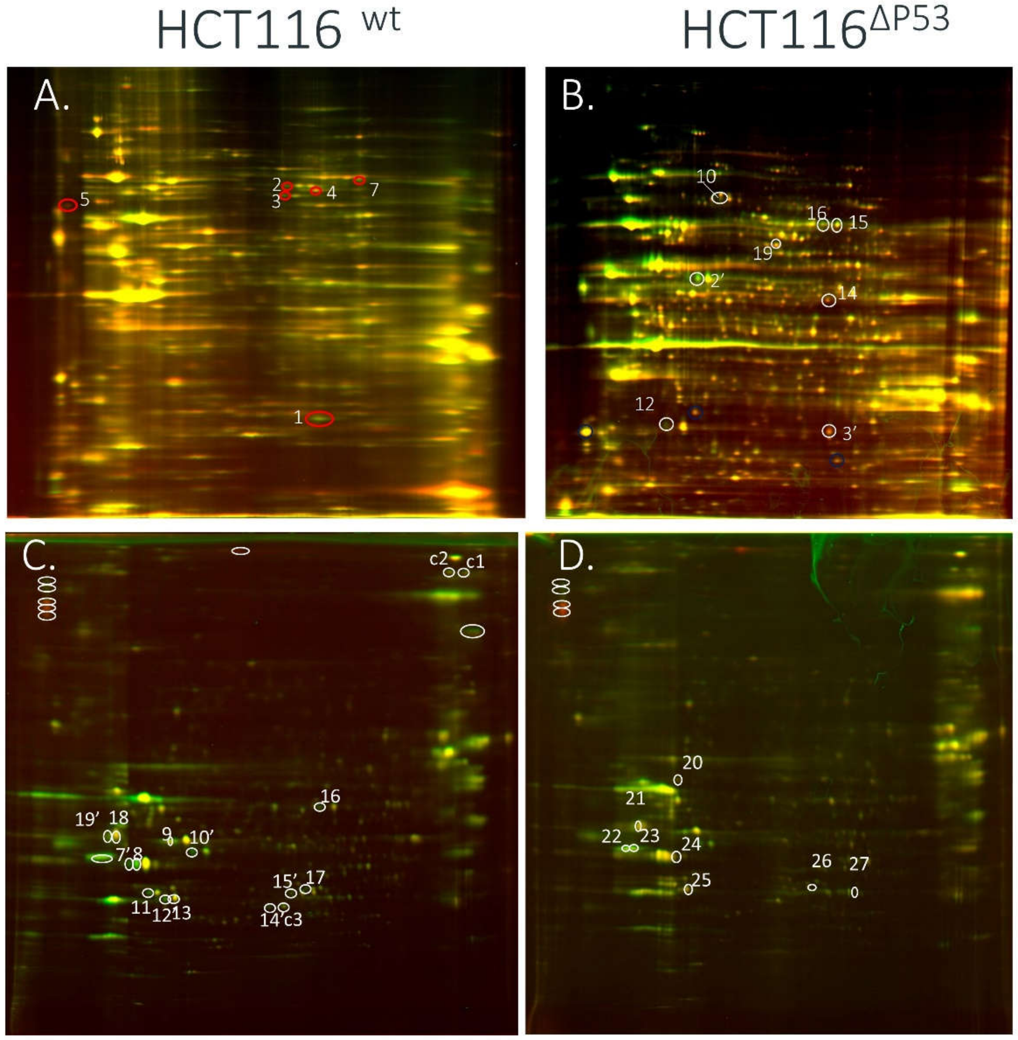

Figure S75. Representative protein gels after 2D electrophoresis are shown. HCT116 sample are proteins from wild type cell line, HCT116ΔTP53 sample was prepared from cells with deleted both alleles of p53 gene after treatment. Protein points for analysis from the gel were selected based on three replicates of 2D gels with inverted sample labeling schemes (correspondingly, one of each three gels is shown). The numbers show points which were trypsinised and identified by LC-MS.

Table S1. Proteins with changed level, determined from 2D-difference gel electrophoresis

Sample	Point on gel	ID	Protein expression	Protein
HCT116 wt	1	UQCRFS1	down	Cytochrome b-c1 complex subunit rieske
HCT116 wt	3	SDHA	down	Succinate dehydrogenase [ubiquinone] flavoprotein subunit
HCT116 wt	4	GPD2	up	Glycerol-3-phosphate dehydrogenase
HCT116 wt	5	MTFP1	up	Mitochondrial fission process protein 1
HCT116 wt	7	LMNA	down	Prelamin-A/C
HCT116 p53 -/-	3'	ABHD10	up	Mycophenolic acid acyl-glucuronide esterase, mitochondrial

HCT116 p53 -/-	14	ALDH7A 1	up	Alpha-aminoadipic semialdehyde dehydrogenase
HCT116 p53 -/-	15	LMNA	down	Prelamin-A/C
HCT116 p53 -/-	10	IMMT	down	MICOS complex subunit MIC60
HCT116 p53 -/-	2'	PDIA3	down	Protein disulfide-isomerase A3
HCT116 p53 -/-	12	PHB	down	Prohibitin 1
HCT116 p53 -/-	19	SDHA	up	Succinate dehydrogenase [ubiquinone] flavoprotein subunit
HCT116 wt	7'	HSPD1	up	Heat Shock Protein Family D (Hsp60) Member 1
HCT116 wt	8	HSPD1	up	Heat Shock Protein Family D (Hsp60) Member 1
HCT116 wt	10'	PDIA3	up	Protein disulfide-isomerase A3
HCT116 wt	11	HSPA8	up	Heat shock cognate 71 kDa protein
HCT116 wt	12'	HSPA9	up	Stress-70 protein, mitochondrial
HCT116 wt	13	HSPA9	up	Stress-70 protein, mitochondrial
HCT116 wt	14'	EZR	up	Ezrin
HCT116 wt	15'	TRAP1	up	Heat shock protein 75 kDa, mitochondrial
HCT116 wt	17	GPD2	up	Glycerol-3-phosphate dehydrogenase
HCT116 wt	16	TUFM	up	Tu Translation Elongation Factor, Mitochondrial
HCT116 wt	18	ATP5F1B	up	ATP synthase subunit beta, mitochondrial
HCT116 wt	19'	ATP5F1B	up	ATP synthase subunit beta, mitochondrial
HCT116 p53 -/-	20	STOML2	up	Stomatin-like protein 2, mitochondrial
HCT116 p53 -/-	21	ATP5F1B	up	ATP synthase subunit beta, mitochondrial
HCT116 p53 -/-	22	P4HB	up	Protein disulfide-isomerase
HCT116 p53 -/-	23	P4HB	up	Protein disulfide-isomerase
HCT116 p53 -/-	24	HNRPK	up	Heterogeneous nuclear ribonucleoprotein K
HCT116 p53 -/-	25	HSPA9	up	Stress-70 protein, mitochondrial
HCT116 p53 -/-	26	TRAP1	up	Heat shock protein 75 kDa, mitochondrial
HCT116 p53 -/-	24	HSPD1	up	Heat Shock Protein Family D (Hsp60) Member 1

Table S2. Anticancer effect of compound **29** achieved after p.o. dose of 170 mg/kg (o.d., 10 days) in P388-inoculated mice

**№**	Tumor volume (mm^3)			
	Control group		Compound **29**	
	day			
	**12**	**15**	**12**	**15**
**1**	305	743	581	1482
**2**	1155	1646	282	778
**3**	444	482	326	1131
**4**	421	695	716	
**5**	527	454	498	1004
***av.* volume**	570	804	481	1099
**SD**	336	488	180	294
**T/C%**	100	100	84	63
**T^{test}**	-	-	0,613	0,326
**P**	-		>0.05	>0.05

Table S3. Acute toxicity study design for compound **29**

**Group**	**Route of administration**	**№ of group**	**Animal gender**	**Number of animals in a group**	**Dose, mg/kg**	**Euthanasia day**
Control	---	1	♂	5	---	29th day of the experiment
Compound **29**	intraperitoneally	2		5	2500	
Control	---	1	♀	5	---	
Compound **29**	intraperitoneally	2		5	2500	

Table S4. Dynamics of body weight of mice in the study of acute toxicity of compound **29** (grams, M ± m)

**Term, days**	**Groups**			
	♂		♀	
	**№ 1 control**	**№ 2 experiment**	**№ 1 control**	**№ 2 experiment**
0	24,5±0,42	24,6±0,53	23,8±0,54	23,8±0,54
7	25,1±1,32	25,2±0,49	23,7±0,62	24,0±1,25
14	26,3±2,05	26,9±0,58	24,5±0,91	24,5±0,91
21	26,2±2,07	26,0±0,63	23,9±0,97	24,0±1,13
29	24,8±1,65	24,1±0,32	25,7±1,12	25,0±1,18

Table S5. Average group indicators of the relative mass of the internal organs of mice in the study of acute toxicity of the compound **29**, %

**Bodies**	**Group**			
	♂		♀	
	**№ 1 control**	**№ 2 experiment**	**№ 1 control**	**№ 2 experiment**
**Liver**	4,87±0,149	5,79±0,320*	5,29±0,178	6,25±0,384*
**Kidneys**	1,48±0,098	1,40±0,060	1,14±0,073	1,14±0,069
**Spleen**	0,33±0,011	0,45±0,032*	0,56±0,007	0,69±0,056
**Heart**	0,53±0,087	0,49±0,013	0,47±0,015	0,43±0,014
**Thymus**	0,05±0,007	0,08±0,021	0,18±0,041	0,24±0,048
**Gonads**	0,68±0,044	0,80±0,033	0,065±0,0075	0,062±0,054
**Lungs**	0,88±0,129	0,76±0,089	0,90±0,114	1,00±0,061
**drenal glands**	0,026±0,0012	0,029±0,0028	0,031±0,0091	0,025±0,0034
**Brain**	1,54±0,066	1,55±0,065	1,51±0,107	1,52±0,074

*— difference compared to control is significant by Student's t-test ($p<0,05$)

[1] Beloglazkina, E. K.; Vatsadze, S. Z.; Majouga, A. G.; Frolova, N. A.; Romashkina, R. B.; Zyk, N. V.; Moiseeva, A. A.; Butin, K. P. Synthesis and electrochemical study of complexes of 2-methylthio-5-(pyridylmethylidene)-3,5-dihydro-4H-imidazol-4-ones with transition metals (Co, Ni, and Cu). Molecular structures of CuIIL1Cl2 (L1 is (5Z)-2-methylthio-3-phenyl-5-(α-pyridylmethylidene)-3,5-dihydro-4H-imidazol-4-one) and CoIIL2Cl2 (L2 is (5Z)-3-methyl-2-methylthio-5-(α-pyridylmethylidene)-3,5-dihydro-4H-imidazol-4-one). Russ. Chem. Bull., 2005, 54, 2771-2782, doi: 10.1007/s11172-006-0190-6

[2] Ivanenkov, Y.A.; Vasilevski, S.V.; Beloglazkina, E.K.; Kukushkin, M.E.; Machulkin, A.E.; Veselov, M.S.; Chufarova, N.V.; Chernyagina, E.S.; Vanzcool, A.S.; Zyk, N.V.; Skvortsov, D.A.; Khutornenko, A.A.; Rusanov, A.L.; Tonevitsky, A.G.; Dontsova, O.A.; Majouga, A.G. Design, synthesis and biological evaluation of novel potent MDM2/p53 small-molecule inhibitors. Bioorg. Med. Chem. Lett. 2015, 25, 404-409, doi: 10.1016/j.bmcl.2014.09.070

[3] Ivanenkov, Y. A.; Majouga, A. G.; Beloglazkina, E. K.; Beloglazkina, A. A.; Veselov, M. S.; Kukushkin, M. E. New dispiro-indolinones, MDM2/p53 interaction inhibitors, method for production and application. RU 2015113026, April 9, 2015.
